# Redox-induced controllable engineering of MnO_2_-Mn_*x*_Co_3-*x*_O_4_ interface to boost catalytic oxidation of ethane

**DOI:** 10.1038/s41467-024-48120-8

**Published:** 2024-05-15

**Authors:** Haiyan Wang, Shuang Wang, Shida Liu, Yiling Dai, Zhenghao Jia, Xuejing Li, Shuhe Liu, Feixiong Dang, Kevin J. Smith, Xiaowa Nie, Shuandi Hou, Xinwen Guo

**Affiliations:** 1 State Key Laboratory of Fine Chemicals, Frontiers Science Center for Smart Materials, School of Chemical Engineering, Dalian University of Technology, Dalian, 116024 P.R. China; 2 SINOPEC Dalian (Fushun) Research Institute of Petroleum and Petrochemicals, Dalian, 116045 P.R. China; 3 Key Lab of Organic Optoelectronics & Molecular Engineering, Department of Chemistry, Tsinghua University, Beijing, 100084 China; 4 Division of Energy Research Resources, Dalian Institute of Chemical Physics, Chinese Academy of Sciences, 457 Zhongshan Road, Dalian, 116023 China; 5 Department of Chemical and Biological Engineering, University of British Columbia, 2360 East Mall, Vancouver, B.C. V6T 1Z3 Canada

**Keywords:** Chemical engineering, Pollution remediation, Heterogeneous catalysis

## Abstract

Multicomponent oxides are intriguing materials in heterogeneous catalysis, and the interface between various components often plays an essential role in oxidations. However, the underlying principles of how the hetero-interface affects the catalytic process remain largely unexplored. Here we report a unique structure design of MnCoO_*x*_ catalysts by chemical reduction, specifically for ethane oxidation. Part of the Mn ions incorporates with Co oxides to form spinel Mn_*x*_Co_3-*x*_O_4_, while the rests stay as MnO_2_ domains to create the MnO_2_-Mn_*x*_Co_3-*x*_O_4_ interface. MnCoO_*x*_ with Mn/Co ratio of 0.5 exhibits an excellent activity and stability up to 1000 h under humid conditions. The synergistic effects between MnO_2_ and Mn_*x*_Co_3-*x*_O_4_ are elucidated, in which the C_2_H_6_ tends to be adsorbed on the interfacial Co sites and subsequently break the C-H bonds on the reactive lattice O of MnO_2_ layer. Findings from this study provide valuable insights for the rational design of efficient catalysts for alkane combustion.

## Abstract (web summary)

The exploration of heterogeneous interfaces between metal oxides has received limited attention. Here the authors demonstrated the creation of MnO_2_-Mn_*x*_Co_3-*x*_O_4_ interfaces through controlled chemical reduction processes, effectively altering electron distribution and yielding a superior catalyst for ethane oxidation.

## Introduction

Low-chain alkanes (C_1_-C_3_) that released from industrial processes, have gained considerable attention because of the growing concerns regarding air quality and human health^1,2^. Of particular interest in ethane, a representative non-methane volatile organic compounds (NMVOCs), has become the focus of regulatory scrutiny due to stringent standards on flue gas emissions^3^. As a result, various technologies have been developed to mitigate their emission. Catalytic oxidation is shown to be an effective approach for eliminating alkanes and their derivatives^4^. However, the development of high-performance catalysts, particularly for low temperature application, remains challenging due to the inherently strong C-H bonds. Additionally, ethane that vastly exists in nature gas (1–9 mol%), must be taken into account during the catalytic nature gas combustion to examine its energetic performance on combustion process as well as its impact on the employed materials. Noble metal-based catalysts (such as Pt or Pd) typically exhibit excellent catalytic activity toward low-chain alkane combustion at low temperature^5^. However, the high cost and limited availability have driven people to find alternatives. With this goal in mind, a substantial amount of work was undertaken to develop efficient non-noble metal-based catalysts^6^.

Special attention has been given to transition metal spinel-type oxides (AB_2_O_4_) because of their remarkable activities and durability in oxidation reactions^7–9^. In a typical AB_2_O_4_ structure, the tetrahedral and octahedral sites are occupied by A^2+^ and B^3+^ cations, respectively, offering a unique atomic arrangement that allows the facile tuning of the redox property^10,11^. Additionally, the interaction between A and B in spinels was identified, which accelerates the generation of reactive oxygen^12^. Moreover, the electron configuration of metal ions in AB_2_O_4_ spinels can be readily tuned by metal doping, thereby alternating the adsorption strength of reactants^13^. However, it should be noted that the obtained spinel oxides may not always present in an ideal AB_2_O_4_ structure, because various factors are involved in the synthesis process^14^. In some cases, certain amount of metal ions may become isolated from their parent spinel grains during crystal growing or post-synthesis process, resulting in the formation of multi-phase oxides. The properties of mixed oxides are more complex than that of the pure spinel because of the involved various interfaces and their coordination environments. Therefore, further investigation is required to elucidate the inherent properties of these interfacial sites as well as optimize the synthesis parameters, to achieve better control over the microstructure of synthesized catalysts.

Interfacial engineering has emerged as an important approach in the design of first catalytic materials, enabling the facilitation of diverse chemical reactions, such as dehydrogenation^15^, CO oxidation^16,17^, and water-gas shift reaction^18,19^. As stimulated by the growing interests in interface catalysis, extensive investigations have been conducted to understand the properties of active sites located within the heterojunction region of mixed oxides. Notably, it has been observed that the interface of multicomponent oxides plays a vital role in facilitating the mass transfer of oxygen. Zhu et al.^20^ found that the proximity between MnO_2_ and CeO_2_ increased the mobility of both surface and lattice oxygen around the grain boundary of MnO_2_-CeO_2_ interface, resulting in an enhanced activity in HCHO removal (T_100_ = 100 °C, GHSV = 90 L h^−1^). Similarly, Zhang et al.^21^ optimized the structure of ZnCo_2_O_4_@CeO_2_ catalyst, and discovered that the nanoscale contacts between ZnCo_2_O_4_ and CeO_2_ introduce an enhanced oxygen storage capacity and lattice oxygen mobility. Shan et al.^22^ adopted the acid-etching approach to create MnO_2_-CoMn_2_O_4_ interfacial system and unveiled that the lattice O that located at interfacial sites was activated due to the weakened Mn-O bonds as well as the altered coordination environments of O atoms. Also, Ren et al.^23^ discovered that the concentration of oxygen vacancies of CoMn_2_O_4_ spinel significantly increased after HNO_3_ treatment, therefore generating more active surface O species during O_2_ activation. Likewise, the established CeO_2_-Co_3_O_4_ interface in CeO_2_@Co_3_O_4_ nanofiber catalysts has proved to be effective in propane oxidation^24^. Moreover, the intimate contact between mixed oxides gives rise to a synergistic catalytic effect, allowing for the simultaneous activation of different reactants. Zhu et al.^25^ investigated the dual interfacial effects between PtFe and FeO_*x*_ in each nanowire (NWs) as well as the interaction between NWs and TiO_2_ support on the PtFe-FeO_*x*_/TiO_2_ catalyst, and discovered their interfacial synergy in CO oxidation. Liu et al.^26^ designed a hierarchical MnO_2_@NiCo_2_O_4_@Ni foam catalyst, and found that the three-dimensional core-shell structure maximizes the interaction between NiCo_2_O_4_ and MnO_2_, consequently improving the performance of NH_3_-SCR at low temperature. Zhang et al.^27^ constructed AgO/CeSnO_*x*_ tandem catalysts and studied the synergistic effects between AgO and CeSnO_*x*_ dual sites for selective oxidation of NH_3_. It is noticed that the electrons on CeSnO_x_ support were more easily transferred to AgO NPs, which accelerates the oxidation activity of AgO and the reduction performance of CeSnO_*x*_ support, thus achieving a good match between NH_3_ oxidation and NO_*x*_ reduction. Also, the strong interaction between different metal oxides affects the dispersion and crystallinity of active centers^28,29^. Furthermore, the electronic property at interfacial region could be flexibly altered by tuning the interaction between different components^30^. These examples emphasize the crucial role of interfaces in multicomponent catalysts. Hence, it is imperative to find out how critical the formed interfaces dictate the performance of complex oxides and further obtain a fundamental understanding on the “property-activity” relationship.

Herein, we report our finding on the manipulation of the MnO_2_-Mn_*x*_Co_3-*x*_O_4_ interface through engineering the Mn/Co ratio of MnCoO_*x*_ catalysts or adjusting the annealing conditions. The resulting materials predominantly exhibit as Mn_*x*_Co_3-*x*_O_4_ spinel oxides with MnO_2_ thin layers decorated on the surface. The catalytic performance of the obtained MnCoO_*x*_ catalysts was evaluated in ethane oxidation to build the correlation between their structure and performance. Our results showed that the presence of MnO_2_ and Mn_*x*_Co_3-*x*_O_4_ at the grain boundary of the involved oxides synergistically enhanced both the ethane adsorption/activation and the lattice oxygen mobility. Notably, the strong interaction between MnO_2_ and Mn_*x*_Co_3-*x*_O_4_ induces a charge rearrangement between these components as supported by the in-situ X-ray Photoelectron Spectroscopy (XPS) analysis and Density Functional Theory (DFT) calculations. Elucidating the role of structural heterogeneity in multicomponent oxides enables us to selectively tune the interface properties and oxygen defects in a wide range of complex oxides.

## Results

### Structure and surface states

**Fig. 1:**
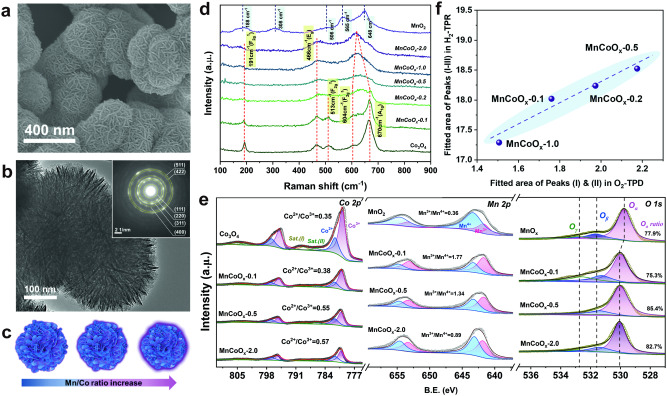
Structural analyses of the as-synthesized MnCoO_*x*_ catalysts. **a** SEM image of MnCoO_*x*_-0.5. **b** TEM image of MnCoO_*x*_-0.5 with an insert showing a corresponding electron diffraction (SAED) pattern. **c** Schematic illustration of the grain boundary of MnCoO_*x*_ with varied Mn/Co ratio. **d** Raman spectra. (Yellow shading area: the vibrational bonds of Co species in MnCoO_*x*_; blue shading area: the vibrational bonds of Mn species in MnO_2_) **e** XPS spectra. **f** A correlation of cumulative area under H_2_ reduction peaks (I & II & III) and O_2_ desorption peaks (I & II) (Dash line: it was drawn to guide the readers’ eyes). (Source Data are provided as a Source Data file).

A series of Mn-substituted cobalt oxides (MnCoO_*x*_-z with varied Mn/Co ratios (z) of 0–2.0) were successfully prepared by chemical reduction method. The obtained MnCoO_*x*_ catalysts present as hierarchical nanospheres with an average diameter of 250-500 nm, which is mainly composed by ultrathin nanosheets with the surface covered by thin layers (Fig. 1a, b; Supplementary Fig. 1). A schematic illustration is presented to show the formed grain boundary layers as a function of Mn/Co ratio (Fig. 1c).

Firstly, the evolution of composition-dependent crystal structure of MnCoO_*x*_ catalysts was examined. Figure 1d presents the Raman spectra of MnCoO_*x*_ catalysts. Note that, the Raman spectra of MnCoO_*x*_-0.1 is similar to that of Co_3_O_4_ reference. While, the main peak of octahedrally coordinated Co sites (CoO_6_: 670 cm^-1^) gradually shifted to lower wavenumber and merged with the shoulder peak (604 cm^-1^) to form a broader peak when Mn/Co ratio is ≥ 0.2, implying the weakened vibration of Co-O bonds. Similar phenomenon was also observed in Ni_*x*_Co_3-*x*_O_4_ spinel^31^. Also, the added Mn ions significant altered the symmetry of CoO_6_, resulting from the lattice replacement induced inhomogeneous distribution of Mn(III) or Co(III/II) ions^32–34^. The induced coordination environmental change further initiates the occurrence of structural defects and lattice distortion on the developed MnCoO_*x*_, which in turn benefits the formation of oxygen vacancies. Besides, the peak position of tetrahedrally coordinated Co (CoO_4_: 191 cm^-1^) was invariant with varied Mn/Co ratio, but their intensity decreased at high Mn/Co ratio due to Mn substitution. Similar result was also obtained from FT-IR analyses (Supplementary Fig. 2). Meanwhile, no active Raman bands belong to Mn-O bonds (as indicated by the blue dash line in Fig. 1d) were observed in the prepared MnCoO_*x*_ catalysts, suggesting that the Mn ions are highly dispersed and/or exist as solid solution in Co_3_O_4_. The bulk structure of MnCoO_*x*_ was further studied by power X-ray diffraction (XRD) (Supplementary Fig. 3, Supplementary Table 1). The results indicate the incorporation of Mn ions into Co_3_O_4_ lattice, leading to the formation of MnCo_2_O_4_ spinel (PDF#23-1237). Also, the selected area electron diffraction (SAED) pattern (the insert of Fig. 1b) is indexed to the cubic lattice typical of MnCo_2_O_4_.

To get more insights of Mn species, X-ray photoelectron spectroscopy (XPS) measurements were performed to investigate the surface states of Mn-O-Co entity (Fig. 1e). Clearly, the surface atomic ratios of Mn/Co measured by XPS (Supplementary Table 2) were higher than that of the corresponding bulk Mn/Co ratio measured by ICP-OES, indicating that part of the Mn ions was dispersed on the surface of MnCoO_x_ catalysts. Notably, the MnCoO_*x*_ presented a high Co^2+^/Co^3+^ ratio of 0.4 ~ 0.6 compared to Co_3_O_4_ (Co^2+^/Co^3+^ = 0.35, Supplementary Table 3), an indicator of Mn substitution into the octahedral sites of Co^3+^. Also, the presence of satellite peaks suggests the partial reduction from Co^3+^ to Co^2+^, which demonstrates the coexistence of Co^2+^ and Co^3+^ on the prepared MnCoO_*x*_ catalysts^35–37^. The Co^2+^/Co^3+^ ratio increases with increased Mn addition and levels off above Mn/Co of 0.5. The formed Mn^3+^ ions increase the anionic defects as Co or Ni does in other spinels, thus benefiting the catalytic oxidation process^38–40^. From Mn 2*p*_3/2_ spectra, we can notice that the MnCoO_*x*_-0.1 catalyst showed the highest Mn^3+^/Mn^4+^ ratio, indicating that more O_v_ are created to maintain the electrostatic balance of the system (4Mn^4+^+O^2-^→2Mn^4+^+2Mn^3+^+□+0.5O_2_)^41–43^. A gradual decrease of Mn^3+^/Mn^4+^ ratio appeared while increasing the Mn/Co ratio due to the diffusion of MnO_2_ onto the surface of Mn_*x*_Co_3-*x*_O_4_ substrate. The average oxidation state (AOS) of Mn 3 s increased with increasing the Mn/Co ratio, which is consistent with Mn 2*p* results (Supplementary Fig. 4). Therefore, we can infer that the added Mn mainly remain in two states, in which part of the Mn is incorporated into the bulk structure of Co_3_O_4_ to form Mn_*x*_Co_3-*x*_O_4_ spinel, and the rest contributes to the formation of MnO_2_ layer or aggregates as determined by the amount of added Mn.

Moreover, the O 1 *s* spectra were fitted into three peaks, which attributed to lattice oxygen (O_α_), surface adsorbed oxygen (or defects, O_β_), and chemisorbed water (O_γ_) with B.E.s of 530.1, 531.3, and 532.7 eV, respectively^44,45^ .The O_α_ species account about 75–85% over MnCoO_*x*_ catalysts, indicating their significant role in oxidation reaction. In addition, O_2_-TPD (Supplementary Fig. 5a) was performed to study the type and mobility of oxygen that contained in the MnCoO_*x*_ catalysts. It was found that the O_2_ desorption peak in the range of 300–600 °C (Region II) obviously shifted towards low temperature on MnCoO_*x*_ catalysts compared to MnO_2_ and Co_3_O_4_ references, implying an improved oxygen mobility after Mn addition. However, the desorption amount in Region II dramatically decreased when Mn/Co ratio is above 0.5, perhaps due to the excessive accumulation of MnO_2_ on the surface. This trend is consistent with what we observed from EPR analysis (Supplementary Fig. 6), indicating that there was more O_v_ on MnCoO_*x*_-0.5.

To clarify the reducibility of involved oxides and the interaction of various species, the H_2_ reduction peak was roughly divided into three individual peaks for MnCoO_*x*_ catalysts with Mn/Co ratio of 0.1–0.5 (Supplementary Fig. 5b). Peak(I) appearing at 100–200 °C belongs to the surface adsorbed O^39^. Noted that the peak (I) accounts for 20% of all the consumed H_2_ on the MnCoO_*x*_-0.1 catalyst, while this value decreased to 10% once more Mn was introduced (Supplementary Tables 2 and 3). The relative amount of peak (II) increased with increased Mn/Co ratio (max.26%), indicating the appearance of MnO_2_ on the surface of MnCoO_*x*_. Also, it is noticeable that the reduction peak (III) shifted towards the lower temperature region (355–375 °C) compared to the bulk Co_3_O_4_ (387 °C), perhaps due to the facile H_2_ transfer from MnO_2_-Mn_*x*_Co_3-*x*_O_4_ interface to the bulk materials. Similar phenomenon was also observed on Mn_2_O_3_@MnO_2_ catalyst via MnO_2_-Mn_2_O_3_ interface^45^. Note that the total integrated area of peaks (I) and (II) in the O_2_-TPD analysis exhibits a linear correlation with the cumulative area under H_2_ reduction obtained from (I), (II), and (III) peaks (Fig. 1f, Supplementary Table 4). However, the excessive amount of Mn shifts peak (III) towards high temperature and even induces the formation of peak (IV), a suggestive of the strong interaction between Mn and Co oxides^12^. To better understand the low-temperature reducibility of MnCoO_*x*_ catalysts, the initial H_2_ consumption rate was calculated and plotted as a function of inversed temperature (1/T), as shown in Supplementary Fig. 7a. Clearly, the initial H_2_ consumption rate decreased in the sequence of MnCoO_*x*_-0.5 > MnCoO_*x*_-0.2 > MnCoO_*x*_-0.1 > MnCoO_*x*_-1.0.

### Catalytic performance evaluation

**Fig. 2:**
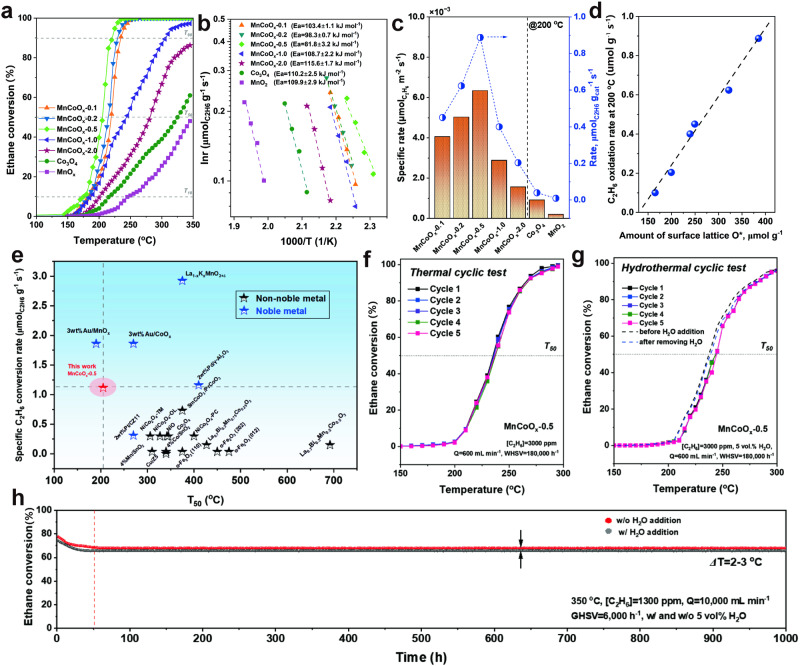
Catalytic performance of MnCoO_*x*_-0.5 catalysts for ethane oxidation. **a** Light-off curves of the as-prepared catalysts (reaction conditions: *ca*. 200 mg catalyst, [C_2_H_6_] = 3000 ppm, Q = 200 mL min^-1^ and WHSV = 60,000 h^-1^). **b** corresponding Arrhenius plots. **c** specific ethane conversion rate at 200 °C. **d** a correlation of the fitted peak area of oxygen species from O_2_-TPD analysis with C_2_H_6_ oxidation rate at 200 °C. **e** comparation of ethane conversion rate at T_50_ with other catalysts reported in the literature (see Table S9 for details). **f** cyclic thermal stability test of MnCoO_*x*_−0.5 catalyst. **g** cyclic hydrothermal stability test of MnCoO_*x*_-0.5 catalyst (reaction conditions: *ca*. 200 mg catalyst, [C_2_H_6_] = 3000 ppm, Q = 600 mL min^-1^, WHSV = 180,000 h^-1^ w/o and w/ 5 vol% H_2_O, respectively). **h** long-term scale up stability tests by 1 wt% MnCoO_*x*_-0.5 coated on micro-monolith substrate for 1000 h (reaction conditions: 350 °C, [C_2_H_6_] = 1300 ppm, Q = 10,000 mL min^-1^, and GHSV = 6000 h^-1^, w/ and w/o 5 vol% of H_2_O). (Source Data are provided as a Source Data file).

To determine the influence of Mn addition, all the synthesized MnCoO_*x*_ catalysts were employed for ethane combustion (Fig. 2a, Supplementary Table 5). Taking the temperature at 50% ethane conversion (T_50_) as an indicator, we found that the oxidation activities decreased in the order of MnCoO_*x*_-0.5 (205 °C) > MnCoO_*x*_-0.2 (215 °C) > MnCoO_*x*_-0.1 (219 °C) > MnCoO_*x*_-1.0 (260 °C) > MnCoO_*x*_-2.0 (282 °C) > Co_3_O_4_ (325 °C) > MnO_2_ (348 °C), suggesting that a small amount of Mn can greatly enhance the activity. However, the activity is sluggish while adding too much Mn, perhaps due to the aggregation of MnO_2_. To further evaluate the commercial potential of MnCoO_*x*_ catalysts, the catalytic activities of other low-chain alkanes (CH_4_ and C_3_H_8_) were tested since they are also contained in the industrial emission (Supplementary Fig. 8, Tables 6–7). It is well-known that the initial H abstraction of short-chain alkanes is often regarded as the key elementary step^7,46^. The strength of C-H bond is closely related to the chain length of alkanes, which in turn determines their reactivity in oxidation reactions. Specifically, the C-H bonds become weaker as the chain length increased (1^st^ C-H bond strength: CH_4_ (465 kJ mol^-1^) > C_2_H_6_ (442 kJ mol^-1^) > C_3_H_8_ (427 kJ mol^-1^))^47^. For comparison, the catalytic activity of Co-based oxides for low-chain alkane (C_1_-C_3_) combustion were summarized in Supplementary Table 8. Clearly, the prepared MnCoO_*x*_-0.5 in this work revealed a better catalytic activity than many of the reported catalysts in the literature.

To further evaluate the catalytic performance of MnCoO_*x*_ catalysts, a kinetic study was completed. Figure 2b presents the Arrhenius plots of MnCoO_*x*_ catalysts for ethane combustion based on the normalized reaction rates at ethane conversion in the range of 5-10%. The obtained apparent activation energy (E_a_) of MnCoO_*x*_ is in the range of 80-116 kJ mol^-1^, exhibiting a volcano-typed trend with increased Mn content. Also, the calculated E_a_ is strongly correlated with the reactivity of MnCoO_*x*_ catalysts. Note that, the E_a_ value of MnCoO_*x*_-0.5 catalyst (E_a_ = 81.8 ± 3.2 kJ mol^−1^) is the lowest, indicating an easier oxidation of C_2_H_6_. Also, the turn-over frequency (TOF) of MnCoO_*x*_-0.5 catalyst for ethane oxidation is 3.93 × 10^-2 ^s^−1^ at 200 °C, which is significantly higher than other MnCoO_*x*_ samples. A good correlation was built between TOF and the initial H_2_ consumption rate for MnCoO_*x*_ catalysts, as shown in Supplementary Fig. 7b. These results suggest that the MnCoO_*x*_-0.5 catalyst with low E_a_ (81.8 ± 3.2 kJ mol^−1^) and high TOF (3.93 × 10^-2 ^s^-1^) is more effective for ethane oxidation on per site basis. Moreover, the effect of space velocity on catalytic activity of MnCoO_*x*_-0.5 catalyst was investigated as shown in Supplementary Fig. 9. Clearly, the ethane conversion decreased with the increased WHSV, as a result of shortening the contact time.

To study the intrinsic activity of MnCoO_*x*_ catalysts, the areal rates normalized by the specific surface area (Supplementary Fig. 10, Supplementary Table 9) of synthesized catalysts (expressed in the unit of µmol m^-2^ s^-1^) were calculated and plotted in Fig. 2c. MnCoO_*x*_-0.5 catalyst showed the highest areal rate (6.3 ×10^-3^ µmol m^-2^ s^-1^), which might be attributed to the strong chemical interaction between Mn and Co oxides, thus creating more effective interfacial sites and further changes the interaction between reactants and lattice O upon Mn substitution. The specific ethane oxidation rate either as per surface area or per mass of prepared catalysts exhibited a similar volcano-typed trend as a function of Mn/Co ratio. This trend is in good agreement with the calculated E_a_. Also, a linear correlation was established between C_2_H_6_ oxidation rate and the amount of surface or subsurface lattice oxygen species, as calculated by the cumulative area of peak (II) in O_2_-TPD results (Fig. 2d). Note that the prepared MnCoO_*x*_-0.5 catalyst showed a superior catalytic performance in ethane oxidation compared to the reported non-noble metal catalysts so far, and even better than several reported noble-metal supported catalysts (Fig. 2e, Supplementary Table 10).

Moreover, the cyclic stability tests were performed both under dry and humid conditions at a relatively high WHSV of 180,000 h^-1^ (Fig. 2f, g, Supplementary Fig. 11). As shown in Fig. 2f, the MnCoO_*x*_-0.5 catalyst was able to be completely oxidized at 295 °C, and showed no attenuation on ethane conversion (Δ*X*_ethane_ < 1%) during thermal cyclic tests. In addition to this, the effect of water vapor was examined. No significant change is observed during the hydrothermal cyclic tests, and the T_90_ value is about 280 °C for all cycles over MnCoO_*x*_-0.5 catalyst (Fig. 2g). Also, the activity almost recovered after H_2_O removal, which suggests the reversible deactivation of MnCoO_*x*_-0.5 catalyst. This reversible deactivation can be substantiated by C_2_H_6_-O_2_/O_2_ + H_2_O TPSR results as shown in Supplementary Fig. 12. Due to its superior performance in our lab scale tests, the MnCoO_*x*_-0.5 powder was chosen and mixed with Al_2_O_3_ to prepare into a suspension for monolith washcoating. A similar preparation method was also used in one of our recently published work^31^. Afterwards, a long-term stability test was performed at 350 °C (Fig. 2h). The ethane conversion slightly dropped from ca. 76 to 68% at the initial stage of the reaction either with or without water addition. After that, no deactivation was observed up to 1000 h time-on-stream (TOS) measurement, which demonstrates the superior water-resistance of monolith MnCoO_*x*_-0.5 catalyst.

### Role of MnO_2_-Mn_*x*_Co_3-*x*_O_4_ interface

**Fig. 3:**
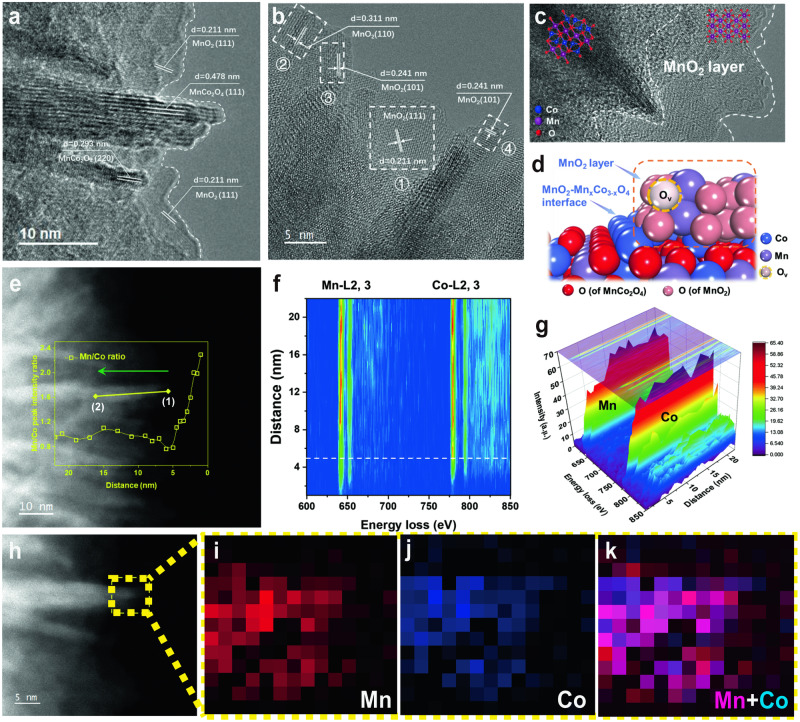
Microstructure characterizations of MnCoO_*x*_ catalyst. **a**, **b** HRTEM images of MnCoO_*x*_-0.5 catalyst at selected interfacial areas. **c** HRTEM image of MnCoO_*x*_-2.0 catalyst. **d** schematic illustration of MnCoO_*x*_-0.5 at interface. **e** high-angle annular dark-field (HAADF) images of MnCoO_*x*_-0.5 catalyst at MnO_2_-Mn_*x*_Co_3-*x*_O_4_ interface with an insert showing the change of Mn/Co ratio along the yellow line from point (1) to (2), as indicated by the green arrow. **f**, **g** Mn-L2, 3-edge and Co-L2, 3 edge spectra as a function of line scanning distance (indicated by the green arrow on (**e**)). **h–k** EELS elemental maps of Mn, Co, and the corresponding Mn-Co overlap of MnCoO_*x*_-0.5 catalyst. Scale bar, 5 nm; Red: Mn, Green: Co. (Source Data are provided as a Source Data file).

To gain a better understanding on the interfacial regions, the aberration-corrected STEM images and EELS analyses were performed to determine the structure and morphology of MnCoO_*x*_ catalysts (Fig. 3, Supplementary Figs. 13–15). An enlarged image on these nanosheets yields a periodic lattice fringe of 0.48 nm, corresponding to the (111) plane of MnCo_2_O_4_, which again confirmed the successful substitution of Mn into the lattice of cubic Co_3_O_4_ (Fig. 3a). Outside the microspheres, some ultra-thin layers were noticeable with an average thickness of ca. 4–5 nm for MnCoO_*x*_-0.5. The measured lattice spacing is about 0.24, 0.21, and 0.31 nm, which can be indexed to the (101), (111), and (110) planes of MnO_2_ (PDF#24-0735), respectively (Fig. 3b). Overall, the HRTEM images provide visual evidence for the formation of MnO_2_-MnCo_2_O_4_ interface, as illustrated in Fig. 3c, d. To better understand the chemical environment of elemental Mn and Co at MnO_2_-Mn_*x*_Co_3-*x*_O_4_ interface, the EELS line-scanning was employed. The elemental distribution from electron energy loss spectra (EELS) clearly showed that Mn is evenly distributed on the shell of MnCo_2_O_4_ microsphere (Fig. 3e–g). Also, the EELS area scanning images give a direct view on the close contact between Co and Mn (Fig. 3h–k). Noted that, Mn prefers to stay on the edge of MnCo_2_O_4_ nanosheets. Next, we employed surface-sensitive technique TOF-SIMS to distinguish the chemical composition between surface and interior of MnCoO_*x*_ catalyst. Supplementary Fig. 16 presents the depth profile of ^55^Mn^+^ and ^59^Co^+^ elements, which again confirms the enrichment of Mn on the surface of Mn_*x*_Co_3-*x*_O_4_ microspheres. Similar conclusion was also obtained on the depth profile of Mn^4+^/Mn^3+^ and Co^2+^/Co^3+^ atomic ratio from the XPS data (Supplementary Fig. 17).

**Fig. 4:**
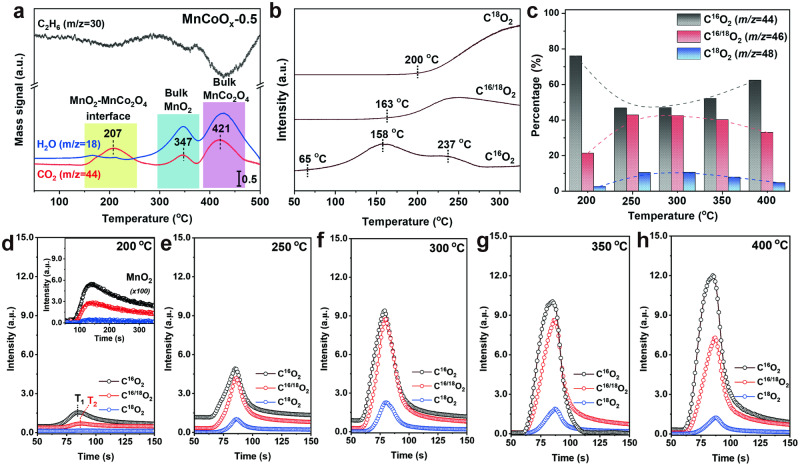
Role of MnO_2_-MnCo_2_O_4_ interface for ethane oxidation. **a** C_2_H_6_-TPSR-MS profile. **b** ^18^O isotopic labeling experiment in the temperature programmed oxidation of ethane over MnCoO_*x*_-0.5 catalyst. **c**–**h** Temporal analysis of products (TAP) of ethane oxidation over MnCoO_*x*_-0.5 as a function of temperature from 200 to 400 °C (the insert of **d** represents the TAP analysis of MnO_2_ reference at 200 °C, T_1_ stands for the maximum temperature of generated C^16^O_2_, T_2_ stands for the maximum temperature of produced C^16/18^O_2_). (Source Data are provided as a Source Data file).

After studying the microstructure of MnCoO_*x*_ catalysts, the properties of MnO_2_-MnCo_2_O_4_ interface were explored. To attain a deeper understanding on the reactivity of MnO_2_-MnCo_2_O_4_ interface, a platform MnO_2_/MnCo_2_O_4_ catalyst with 1 wt% of Mn loading was synthesized. Firstly, C_2_H_6_-TPSR was carried out to study the properties and reactivity of involved O on MnCoO_*x*_-0.5 (Fig. 4a). Both CO_2_ (*m/z* = 44) and H_2_O (*m/z* = 18) were detected in the tested temperature range (50–500 °C). After studied the O reactivity of MnO_2_, MnCo_2_O_4_, and MnO_2_/MnCo_2_O_4_ references (Supplementary Fig. 18), we deduce that the evolved CO_2_ peak below 250 ^o^C (as indicated in the yellow box) can be ascribed to the oxygen that is located at or near MnO_2_-MnCo_2_O_4_ interfacial region, while the high-temperature peak above 400 °C (as indicated in the pink box) is assigned to the bulk MnCo_2_O_4_ substrate. Besides, a relatively weak CO_2_ peak appeared at 347 °C (as indicated in the blue box), suggestive of the existence of a small portion of aggregated MnO_2_. Comparatively, the C_2_H_6_-TPSR result of MnCoO_*x*_-0.5 catalyst indicates the reactive nature of surface lattice O that located at the interface of MnO_2_-MnCo_2_O_4_. To get more insights into the activity of lattice oxygen (O_Latt_) near MnO_2_-MnCo_2_O_4_ interfacial areas, two DRIFT-MS experiments were designed. One was carried out in an O_2_-free environment under isothermal conditions, and the other experiment was performed in transient state. Notably, CO_2_ was detected on the MnCoO_*x*_-0.5 catalyst without gas-phase O_2_ supply, indicating the participation of lattice O at 250 °C (Supplementary Fig. 19). The transient DRIFT-MS analysis showed that the transition period for O_2_-depletion follows the trend of MnCoO_*x*_-0.5 (*Δ*t = 210 s) > MnO_2_/MnCo_2_O_4_ (*Δ*t = 106 s) > MnO_2_ (*Δ*t = 90 s) (Supplementary Fig. 20), which is in accordance with the isotherm experiments.

Following this result^18^O_2_ isotopic labeling experiments were performed to monitor how the lattice oxygen was involved in ethane oxidation. The formation of C^16^O_2_ (*m*/*z* = 44) became noticeable above 65 °C, indicating the active nature of O_latt_ on MnCoO_*x*_-0.5 (Fig. 4b). Noted that, C^16^O_2_ doublet peak appeared (158 and 237 °C), which represent two types of lattice O. As the reaction proceeds, the formation of C^16/18^O_2_ occurs (163 °C) accompanied with the gradual decline of C^16^O_2_, indicating that the oxygen exchange was taking place between gas phase ^18^O_2_ and lattice ^16^O from the catalyst. Followed by this, the formation of C^18^O_2_ is initiated (200 °C) due to the depletion of surface lattice ^16^O and the ^18^O_2_ replenishment. The obtained isotope results emphasized the effectiveness of lattice O in MnCoO_*x*_-0.5. For MnO_2_ reference, the lattice ^16^O could also participate in oxidation, but with a higher onset temperature (189 °C), an indicator of the low activity of O_latt_ (Supplementary Fig. 21). Also, the presence of C^16^O_2_ (or H_2_^16^O) single peak suggested that there is only one type of lattice O participating in the reduction process, which is distinct from MnCoO_*x*_-0.5. Overall, these isotopic O exchange studies suggests that the ethane oxidation is dominated by a surface Mars-van Krevelen (MVK) mechanism in both cases.

Subsequently, temporal analysis of products (TAP) was undertaken to unveil the dynamic surface change of MnCoO_*x*_-0.5 and MnO_2_ reference as a function of temperature (Fig. 4c–h, Supplementary Fig. 22). During each test, a small quantity of reactant mixture (5 ml, C_2_H_6_ + ^18^O_2_ + He) was injected in the temperature range of 200–400 °C to facilitate the scrambling of ^18^O/^16^O atoms, thereby making it possible to capture the initial catalytic behavior of the material. Despite the quantitative difference in product distribution between steady-state and TAP experiments, the general selectivity trends were consistent. Note that the amounts of ^16^O-containing products (C^16^O_2_ and C^16/18^O_2_, accounts for >95%) significantly exceed that of C^18^O_2_ at 200 ^o^C on the MnCoO_*x*_-0.5 catalyst. Upon combining with the results obtained from C_2_H_6_-TPSR analysis, we can confidently verify that the majority of the participated O arises from the lattice O that resides at MnO_2_-MnCo_2_O_4_ interface, exhibiting a remarkable reactivity in promoting oxidation reactions, particularly at relatively lower temperatures. Also, we found that the activity of lattice O on MnCoO_*x*_-0.5 is significantly higher than that of bulk MnO_2_ (insert of Fig. 4d). At 250 °C, the surface lattice ^16^O is quickly consumed as indicated by the increase of C^16/18^O_2_ and C^18^O_2_. However, once the temperature is above 300 °C, the amount of ^16^CO_2_ slightly increased due to the enhanced bulk phase O migration/diffusion to refill the surface O_v_ at high temperature. Thereby, we can infer that the replenishment of O_v_ originates from a conjugated effect both from the gaseous O_2_ and bulk phase O migration/diffusion, in which the contribution from the latter could be enhanced at high temperature. Also, the results evidently conclude that the lattice O stayed at MnO_2_-MnCo_2_O_4_ interfaces plays a crucial role for low-temperature ethane activation.

**Fig. 5:**
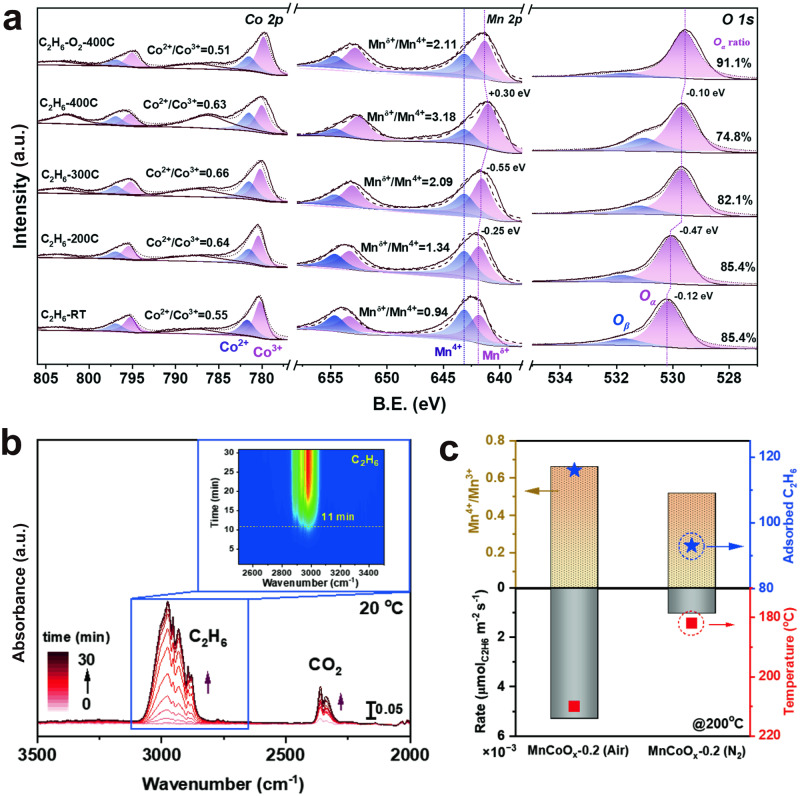
Properties of MnO_2_-MnCo_2_O_4_ interface for ethane oxidation. **a** in-situ XPS analysis of MnCoO_*x*_-0.5 catalyst under different gas atmospheres. **b** DRIFT spectrum of ethane adsorption over MnCoO_*x*_-0.5. **c** The correlation between ethane conversion rate (or temperature at constant rate of 0.21 µmol g_cat_^−1^ s^−1^) and Mn^4+^/Mn^3+^ ratio (or C_2_H_6_ adsorption capacity). (Source Data are provided as a Source Data file).

Aside from this, the in-situ XPS analyses (Fig. 5a, Supplementary Table S11) showed that the ratio of Co^2+^/Co^3+^ quickly increased from 0.55 (fresh sample at RT) to 0.64 (C_2_H_6_ at 200 °C) with no more change above 200 °C, perhaps due to the efficient electron transfer from the absorbed C_2_H_6_ to the positively charged Co ions. While from Mn 2*p* spectra, we observed the significant increase of Mn^δ+^/Mn^4+^ ratio from 0.94 (fresh sample at RT) to 3.18 (C_2_H_6_ at 400 °C) accompanied by the shifting of Mn^δ+^ peak towards lower B.E., suggesting the consumption of lattice O on MnO_2_ domains during H abstraction, thereby resulting in a coordination change on Mn species. Once C_2_H_6_/O_2_ mixture was introduced into the system, the Mn^δ+^/Mn^4+^ ratio slightly increased from 3.18 (C_2_H_6_ at 400 °C) to 2.11 (C_2_H_6_/O_2_ at 400 °C), while the Co^2+^/Co^3+^ ratio almost went back to its original states. This result again indicates the participation of lattice oxygen from MnO_2_ layer. Also, the in-situ XPS results revealed that the O_α_ (lattice O) peak gradually shifts towards lower B.E. with increased C_2_H_6_ reduction temperature, indicating the weakened interaction between Co/Mn and O atoms, potentially resulting in an increase in oxygen vacancies^48^. The O_α_ species gradually consumed during C_2_H_6_ reduction from 85.4% (fresh catalyst at RT) to 74.8% (C_2_H_6_ at 400 °C). This observation further confirmed the participation of lattice O species over the MnCoO_*x*_-0.5 catalyst during C_2_H_6_ oxidation, which is consistent with the isotopic labeling experiments.

Next, the adsorption of C_2_H_6_ over MnCoO_*x*_ catalysts was investigated. As shown in the time-resolved DRIFT spectra (Fig. 5b), the intensity of ethane adsorption bands (3000 cm^-1^) gradually increased with time-on-stream operation to reach a steady-state level. The MnCoO_*x*_-0.5 exhibited the strongest ethane adsorption capacity compared to MnCo_2_O_4_ and MnO_2_ references (Supplementary Fig. 23). Interestingly, the time it took to detect ethane follows the order of MnCoO_*x*_-0.5 (11.0 min) > MnCo_2_O_4_ (7.5 min) > MnO_2_ (4.0 min), indicating that more C_2_H_6_ are adsorbed/activated over MnCoO_*x*_-0.5 catalyst. Again, CO_2_ was detected at 2300–2400 cm^-1^, which indicates the participation of lattice O. Moreover, C_2_H_6_-TPD was employed to address the chemisorption behavior of C_2_H_6_ over MnCoO_*x*_. As shown in Supplementary Fig. 24, CO, CO_2_, and H_2_O as main products were detected due to the reduction of C_2_H_6_ from lattice O, but with different desorption temperatures. Also, the integrated peak area of produced C-related species followed a decreasing trend of MnCoO_*x*_-0.5 > MnCo_2_O_4_ > MnO_2_, suggesting that more ethane was preserved over MnCoO_*x*_-0.5 catalyst.

Furthermore, the ethane oxidation activity of MnCoO_*x*_-0.5 is compared to MnO_2_/MnCo_2_O_4_, MnCo_2_O_4_, and MnO_2_ references, to identify the catalytic contribution of MnO_2_-MnCo_2_O_4_ interface (Supplementary Fig. 25). Clearly, the areal rate of MnCoO_*x*_-0.5 (1.35 × 10^-2^ μmol m^-2^ s^-1^, 220 °C) is close to that of MnO_2_/MnCo_2_O_4_ (1.14 × 10^-2^ μmol m^-2^ s^-1^, 220 °C), indicating that the high conversion of MnCoO_*x*_-0.5 catalyst may result from the presence of MnO_2_-MnCo_2_O_4_ interface. Noted that the temperature of T50 dramatically reduced to 304 C for the physically mixed MnO_2_ and MnCo_2_O_4_ (referred to as Phy-MnCo_2_O_4_-MnO_2_) catalyst compared to pure MnO_2_. A similar performance was obtained on the layer-packed MnCo_2_O_4_-MnO_2_ (refers to as LP_MnCo_2_O_4_-MnO_2_, T_50_ = 311 °C). However, the catalytic activity of MnCo_2_O_4_ and MnO_2_ mixtures was lower than that of the MnO_2_/MnCo_2_O_4_ model catalyst regardless of their mixing methods, indicating the significant role of interfacial sites due to the proximity between the two components. In this regard, it is imperative to study the correlation of MnO_2_-MnCo_2_O_4_ interface with catalytic properties. Therefore, several control experiments were designed by annealing the MnCoO_*x*_ precipitates under N_2_ and air, respectively. It was found that the number of MnO_2_-MnCo_2_O_4_ interfacial sites can be altered based on the strong O_2_ affinity of Mn, which is similar to the synthesis of core/shell Au/MnO and PtFe-FeO_*x*_/TiO_2_ catalysts^25,49^. From XPS analysis, we know that there are more high valence Mn species appeared on the surface of the air calcined MnCoO_*x*_-0.2 catalyst compared to the N_2_-treated one, as evidenced by the high AOS value and Mn^4+^/Mn^3+^ ratio (Supplementary Fig. 26). Hence, it is reasonable to deduce that more Mn species diffuse out onto the Mn_*x*_Co_3-*x*_O_4_ spinel surface forming MnO_2_ domains due to the strong O_2_ driving force and consequently, creating more MnO_2_-MnCo_2_O_4_ interfaces. Also, the C_2_H_6_-TPD results showed that the MnCoO_*x*_-0.2-Air has a strong C_2_H_6_ storage capacity compared to that of MnCoO_*x*_-0.2-N_2_ (Supplementary Fig. 27). Eventually, a positive correlation was established between ethane conversion rate and Mn^4+^/Mn^3+^ ratio, which proved the highly effective of MnO_2_-MnCo_2_O_4_ interface in catalyzing ethane oxidation (Fig. 5c, Supplementary Fig. 28).

### Surface mechanism

**Fig. 6:**
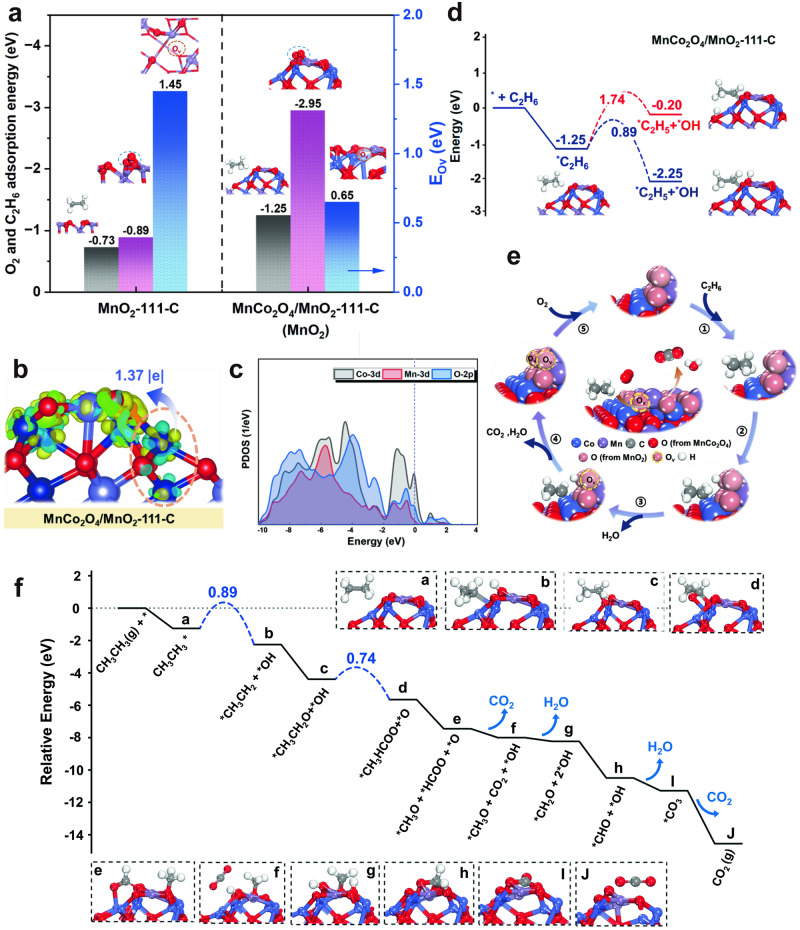
Mechanistic study of ethane oxidation over the MnCoO_*x*_-0.5 catalyst. **a** The calculated adsorption energy of C_2_H_6_, O_2_, and the O_v_ formation energy on the bulk MnO_2_−111-C and MnCo_2_O_4_/MnO_2_-111-C catalyst models (Note: The adsorption energy of C_2_H_6_ was obtained by adsorbing C_2_H_6_ at the interfacial region of MnCo_2_O_4_/MnO_2_-111-C model; the O_2_ adsorption energy was obtained by adsorbing O_2_ on the upper MnO_2_ cluster of MnCo_2_O_4_/MnO_2_-111-C model). **b** the calculated differential charge density between O atom in the upper MnO_2_ cluster and the interfacial Co atom of MnCo_2_O_4_/MnO_2_-111-C. **c** the calculated projected density of states (PDOSs) of Co-3*d*, Mn-3*d* and O-2*p* orbital on the MnCo_2_O_4_/MnO_2_-111-C (the Fermi level was set to zero and the isosurface value was set to 0.005 e Å^-3^; the cyan and yellow regions represent positive and negative charges, respectively). **d** energy profiles for the dissociation of the first C-H bond of C_2_H_6_ over MnCo_2_O_4_/MnO_2_-111-C model (red line: H abstraction of adsorbed C_2_H_6_ at the interfacial region of MnCo_2_O_4_/MnO_2_ model catalyst; blue line: H abstraction of adsorbed C_2_H_6_ at the upper MnO_2_ cluster of the MnCo_2_O_4_/MnO_2_ model catalyst). **e** schematic illustration of the reaction mechanism of ethane oxidation over MnCoO_*x*_−0.5 catalyst (① C_2_H_6_ adsorption; ② Initiated 1^st^ H abstraction; ③ Continuous H abstraction; ④ CO_2_ and H_2_O desorption; ⑤ Refilling O_v_ by O_2_). **f** Energy diagram of the optimal reaction paths for ethane oxidation on MnCo_2_O_4_/MnO_2_-111-C catalyst surface and the optimized structures of all species involved. (Source Data are provided as a Source Data file).

Density functional theory (DFT) calculations were carried out to further assess the effects of MnO_2_-MnCo_2_O_4_ interface and provide information on how the constructed interface contributes to the catalytic behaviors, especially in terms of the interactions with reactants. Similar to one of our recent work^31^, we constructed the MnCo_2_O_4_ crystal structure by replacing part of the octahedral Co atoms of cubic Co_3_O_4_ with Mn. As shown in supplementary Fig. 29a, the Type (II) model was found to be the most stable structure in our calclation by substituting octahedral Co^3+^ with Mn^3+^, as demonstrated by the lowest relative energy per Mn atom in the proposed MnCo_2_O_4_ models. The obtained lattice parameter of MnCo_2_O_4_ spinel is enlarged from 8.07 to 8.14 Å, which is consistent with the XRD results. Meanwhile, the bulk MnO_2_ models exposed with (111), (110), and (101) facets as well as the MnCo_2_O_4_ (111) facets (Supplementary Fig. 29b, c) were built to correlate with what we observed from the HRTEM images (Fig. 3). After analyzing the termination stability of MnO_2_ and MnCo_2_O_4_, the optimized interfacial models of MnO_2_-MnCo_2_O_4_ were established by taking MnCo_2_O_4_-111-A as the underlying substrate and intercepting a structural unit from MnO_2_-111-C, MnO_2_-110-B, and MnO_2_-101-B as the upper cluster (named as MnCo_2_O_4_/MnO_2_-111-C, MnCo_2_O_4_/MnO_2_-110-B, and MnCo_2_O_4_/MnO_2_-101-B, respectively, see details in Supplementary Figs. 30–31). Figure 6a showed the adsorption energy of C_2_H_6_ and O_2_ as well as the oxygen vacancy formation energy (E_Ov_) on the bulk MnO_2_ and MnCo_2_O_4_/MnO_2_ catalyst models. Taken MnCo_2_O_4_/MnO_2_-111-C as an example, we can clearly see that the adsorption energy of C_2_H_6_ at the interface of MnCo_2_O_4_/MnO_2_-111-C model (-1.25 eV) is negatively higher than the corresponding bulk MnO_2_-111-C (-0.73 eV), indicating the preferential adsorption of C_2_H_6_ on the former catalyst. Also, the adsorption of O_2_ at the interface of MnCo_2_O_4_/MnO_2_-111-C model (−1.01 eV) is negatively less than that of C_2_H_6_ (-1.25 eV), indicating that O_2_ cannot compete with C_2_H_6_ for the adsorption at MnO_2_-MnCo_2_O_4_ interface (Supplementary Fig. 32a). Similar results were also obtained on other interfacial models (MnCo_2_O_4_/MnO_2_-110-B and MnCo_2_O_4_/MnO_2_-101-B, Supplementary Fig. 32b, c).

Interestingly, we found that the O_2_ molecule is prone to be activated on the topmost MnO_2_ domain of the MnCo_2_O_4_/MnO_2_-111-C catalyst as evidenced by the partial electron transfer from MnCo_2_O_4_ sublayer to MnO_2_ via the interfacial Co cations to O anions that located at the adjacent of MnO_2_ cluster (1.37 |e |), as shown in Fig. 6b. In addition, the calculated projected density of states (PDOS) shows a upshift of O *p*-band near Fermi level, indicating a strong interaction between Co 3d and O 2p orbitals. The enhanced C_2_H_6_ adsorption can also be explained by the strong hybridization between O 2*p* and Co-3*d*/Mn-3*d* orbitals (Fig. 6c). To further confirm this, we carried out a crystal orbital Hamilton population (COHP) calculation to get a quantitative analysis of the interfacial O-Co bond interaction of MnCo_2_O_4_/MnO_2_ interfacial models (Supplementary Fig. 33). The integral values below the Fermi level are -1.74 over MnCo_2_O_4_/MnO_2_-111-C catalyst, which again demonstrated the significant hybridization between O and Co sites. Additionally, we calculated the adsorption energy of C_2_H_6_ on the *x*Co/MnO_2_ (*x* = 1–2) model catalysts by varying the Co content on different MnO_2_ planes to gain a better understanding of the Co-O-Mn sites and their effects on C_2_H_6_ activation (Supplementary Fig. 34). Noticeably, the adsorption strength of C_2_H_6_ increases with increasing the Co substitution contents, indicating a positive effect of Co sites on C_2_H_6_ adsorption, which aligns with the experimental results (Supplementary Fig. 35). Meanwhile, the lowest oxygen vacancy formation energy (E_Ov_ = 0.65 eV) was obtained on the uppermost MnO_2_ domain of MnCo_2_O_4_/MnO_2_-111-C model compared to other proposed catalyst models, which confers a better O_2_ adsorption ability on this catalyst (Supplementary Fig. 32d). Also, the average Mn-O bond length of MnCo_2_O_4_/MnO_2_-111-C (1.94 Å) is larger than that of MnO_2_-111 (1.83 Å), which implied a high O mobility on the former model (Supplementary Fig. 36). Therefore, the significant influence from the underlying spinel MnCo_2_O_4_ was identified.

To understand the underlying mechanism of C_2_H_6_ oxidation over the MnCoO_*x*_-0.5 catalyst, a detailed discussion of the first C-H bond dissociation of C_2_H_6_ was carried out on the MnCo_2_O_4_/MnO_2_-111-C model, because this step was typically being regarded as the kinetically relevant step^31^. As shown in Fig. 6d, two reaction pathways were proposed based on the position of the abstracted H, either bind to the O sites of the upper MnO_2_ cluster or to the underlying MnCo_2_O_4_ substrate, eventually forming OH groups. The obtained results showed that the energy barrier (ΔE_TS_) of C-H bond cleavage on the O sites of MnO_2_ cluster (ΔE_TS_: 0.89 eV) is lower than that on the MnCo_2_O_4_ substrate (ΔE_TS_: 1.74 eV), indicating that the former route is kinetically more favorable. Moreover, the formation of OH group from C_2_H_6_ dissociation on the upper MnO_2_ clusters is thermodynamically more favorable by releasing energy of 1.00 eV, whereas the OH group formation on the MnCo_2_O_4_ substrate is endothermic by 1.05 eV. Therefore, the lattice oxygen species of MnO_2_ domain plays a significant role in C_2_H_6_ oxidation, as evidenced by both experimental and DFT results. Similar trends were also obtained on the other two interfacial models (MnCo_2_O_4_/MnO_2_-110-B and MnCo_2_O_4_/MnO_2_-101-B, Supplementary Fig. 37). Compared to the MnCo_2_O_4_-111-A model without MnO_2_ domain, the C-H bond dissociation barrier (1.27 eV) is higher than that obtained on the MnCo_2_O_4_/MnO_2_-111-C interfacial model, inferring an interfacial engineering of MnO_2_-Mn_*x*_Co_3-*x*_O_4_ catalyst to boost ethane oxidation. Here, a schematic illustration of the reaction mechanism was proposed and illustrated in Fig. 6e. Subsequently, the energy diagram of elementary steps for ethane oxidation along the reaction pathways was calculated to gain a deeper understanding on the MnO_2_-MnCo_2_O_4_ interfacial system, as illustrated in Fig. 6f. After dissociating the first C-H bond of C_2_H_6_, the generated ^*^CH_3_CH_2_ species is prone to bond on Co sites that located at the interface of MnO_2_ and MnCo_2_O_4_ substrate (Fig. 6f, b), which aligns with the C_2_H_6_-TPSR results. Then, the adsorbed ^*^CH_3_CH_2_ changes its adsorption site from interfacial Co to the lattice O^*^ of upper MnO_2_ cluster to form ^*^CH_3_CH_2_O (Fig. 6f, c), which is proved to be thermodynamically favorable by releasing an energy of 2.14 eV. This calculation is in line with our in-situ XPS results, which implies that further dehydrogenation mostly occurs on the upper MnO_2_ domains. After that, the produced ^*^CH_3_CH_2_O entities undergo further dehydrogenation, resulting in the formation of ^*^CH_3_HCOO intermediates (Fig. 6f, d). These intermediates subsequently decompose into^*^CH_3_O and ^*^HCOO by breaking the C-C bonds, releasing an energy of 1.8 eV. Finally, the continuous dehydrogenation of ^*^CH_3_O and ^*^HCOO leads to the formation of ^*^CH_2_O^*^,CHO, CO_2_, and H_2_O species, showing a downhill energy profile. Overall, DFT results are consistent with the in-situ DRIFT studies (Supplementary Fig. 39) and confirm that the first C-H bond cleavage of C_2_H_6_ is the rate-determining step in ethane combustion on the MnCo_2_O_4_/MnO_2_ interfacial catalyst, which has a barrier of 0.89 eV. Based on the above analyses, we can reasonably conclude that the simultaneous enhancement on ethane adsorption/activation and lattice O mobility of MnCoO_*x*_-0.5 catalyst is proved to be the main reason of achieving an excellent activity in ethane oxidation, which is ingeniously controlled by interfacial engineering.

## Discussion

In summary, we have successfully developed the MnCoO_*x*_ catalyst by a facile chemical reduction synthesis method, which shows the highest specific reaction rate in ethane combustion beyond all the reported non-noble metal catalysts, as well as an excellent long-term stability up to 1000 h even under humid conditions. Mn with strong O affinity tends to diffuse out onto the spinel surface forming MnO_2_ domains during an O_2_-rich environment. The established interaction between MnO_2_ and Mn_*x*_Co_3-*x*_O_4_ triggers the construction of interfacial sites. Surprisingly, the Co sites on the established hierarchical interface of MnO_2_-Mn_*x*_Co_3-*x*_O_4_ exhibit a preferential adsorption on ethane; while, the MnO_2_ layer displays a strong ability of doing H abstraction on their active lattice O, and further proceed the ethane oxidation through a redox pathway at interfacial regions. Revealing the essential role of interface provides an effective strategy of regulating the coordination environment of involved components as well as their electron transfer ability.

## Methods

### Materials

Potassium permanganate (VII) (KMnO_4_ powder, ≥99.0 %), manganese (II) nitrate tetrahydrate (Mn(NO_3_)_2_·4H_2_O, ≥97%) and cobalt (II) nitrate hexahydrate (Co(NO_3_)_2_·6H_2_O, ≥97%) purchased from Sinopharm, were used as received without further purification.

### Catalyst preparation. (1) Synthesis of MnCoO_*x*_

(1) **Synthesis of MnCoO**_**x**_: MnCoO_*x*_ were synthesized by a redox-controlled synthesis method (Mn^7+^ + 3Co^2+^ → Mn^4+^ + 3Co^3+^). In a typical synthesis process, the Mn (VII) solution was prepared by dissolving certain amounts of KMnO_4_ into 1000 mL deionized water under magnetic stirring for 30 min at 70 °C. The Co (II) solution was prepared by dissolving specific amounts of Co(NO_3_)_2_·6H_2_O into aqueous solution with certain amounts of potassium citrate under magnetic stirring. Subsequently, the prepared Co precursor solution was added dropwise into the KMnO_4_ solution at a specific injection speed to control the reduction process. After completed the injection, the mixed solution was keeping stirring for another 2 h at 80 °C. Then, the mixed solution was maintained under ambient conditions. After aging for a few hours, the black precipitate was collected by filtration, and washed by deionized water and absolute ethanol three times before drying. After that, the precursor was subjected to an annealing treatment in static air at 350 °C for 2 h at a ramping rate of 1 °C min^-1^. Finally, the resulting catalysts were washed by 1 M NH_4_NO_3_ solution for 2 h at room temperature under stirring to remove K ions prior to the catalytic tests. The obtained catalysts were denoted as MnCoO_*x*_-z, where z represents the nominal molar ratio of Mn/Co. The synthesis parameters of MnCoO_*x*_ catalysts are given in Table S10. (2) **Synthesis of Co**_**3**_**O**_**4**_: A typical precipitation method was employed to prepare Co_3_O_4_ reference by adding ammonia (1 mol L^-1^) into cobalt nitrate solution. After vigorous stirring for 2 h, the solution was filtered and washed three times by deionized water and ethanol. The obtained precipitate was dried at 70 °C for 12 h and followed by annealing in air at 400 °C for 3 h. (3) **Synthesis of MnO**_**2**_: MnO_2_ nanoparticles (NPs) were synthesized by using KMnO_4_ solution as Mn precursor to take K effects into account. The precursor was prepared according to the procedure described elsewhere^[^^50,51^^]^. Typically, oleic acid (10.0 mL) was added to KMnO_4_ solution (0.0126 mol L^-1^). After vigorous stirring for 30 min, the emulsion was washed by water and ethanol three times to remove residuals. Then, the product was dried in air at 80 °C overnight before calcinated in air. Finally, the obtained precursor was treated at 200 °C in air for 5 h. (4) **Synthesis of MnCo**_**2**_**O**_**4**_ **and MnO**_**2**_**/MnCo**_**2**_**O**_**4**_: MnCo_2_O_4_ support was synthesized by a conventional precipitation method. Typically, ammonia (1 mol L^-1^) was added dropwise to the solution of Mn(NO_3_)_2_·4H_2_O (0.19 mol L^-1^) and Co(NO_3_)_2_·6H_2_O (0.38 mol L^-1^). After vigorous stirring for 6 h, the mixture was filtered and dried. The obtained powder was calcinated at 350 °C for 4 h. The resulting sample was denoted as MnCo_2_O_4_. A supported 1%MnO_2_/MnCo_2_O_4_ catalyst was prepared by chemically reducing Mn on the obtained MnCo_2_O_4_ support. Firstly, 1 g of MnCo_2_O_4_ support was dispersed in 25 mL deionized water. After that, 7.286×10^-5 ^mol KMnO_4_ was added into the MnCo_2_O_4_ dispersed solution and stirring for 30 min. Followed by this, 1.09 × 10^-4 ^mol Mn(NO_3_)_2_ was added to reduce KMnO_4_. The mixture was stirred for another 30 min before increasing the temperature to 70 °C for 2 h. The obtained precipitate was filtered and washed by water and ethanol three times. The obtained precursor was firstly dried at room temperature for 24 h, and then dried at 70 °C for another 12 h. Eventually, the as-prepared sample was calcined at 350 °C for 2 h.

### Characterization

The specific surface area, pore volume, and averaged pore size were determined from N_2_ adsorption-desorption isotherms measured at -196 °C using a Micromeritics ASAP 2020 analyzer. All samples were degassed at 200 °C (100 μm Hg) for 6 h. The specific surface area (S_BET_) was calculated from the measured N_2_ isotherm using the Brunauer-Emmett-Teller (BET) equation applied in a relative pressure range (P/P_o_) of 0.01-0.35. The total pore volume (V_total_) was obtained from N_2_ uptake at a relative pressure of P/P_o_ = 0.99. The averaged pore size was calculated by 4V_total_/S_BET_. The elemental analysis was conducted by inductively coupled plasma-optical emission spectroscopy (ICP-OES). An acid digestion was conducted by aqua regia at 100 °C to dissolve all metals.

Powder X-ray diffraction (XRD) patterns were performed on a Rigaku SmartLab 9 kW diffractometer with Cu Kα (*λ* = 1.5406 Å) radiation operating at 45 kV and 200 mA to determine the bulk structure of the synthesized materials. The scanning speed was set at 10 s/step with 2*θ* in the range of 10^o^ to 70^o^.

Raman scattering spectra were collected on a DRX Microscope instrument (Thermo Fisher Scientific) with an exciting wavelength of λ_ex._ =  532 nm equipped with a charge coupled device (CCD) detector at ambient conditions. The scanning range was set at 100–1000 cm^-1^ with resolution of 1.0 cm^-1^. The attenuated total reflection Fourier Transform Infrared Spectroscopy (ATR FT-IR) spectrum were collected by Thermo Nicolet iS50 spectrometer from 400 to 4000 cm^-1^. The electron paramagnetic resonance (EPR) spectra were obtained on Bruker EPR equipment (model a220-9.5/12) at room temperature by detecting the unpaired electron.

Field emission scanning electron microscopy (FESEM) images were obtained with a Hitachi SU8220 instrument with an acceleration voltage of 5 kV to reveal the morphology of MnCoO_x_ catalysts. The average particle size was calculated by accounting >100 particles/clusters and then fitting the measured size to a normal distribution. Energy dispersive X-ray (EDX) elemental analyses were used to reveal the elemental composition of MnCoO_x_ catalysts. The high-resolution transmission electron microscopy (HRTEM) images and electron energy loss spectroscopy (EELS) spectrum images were recorded on Thermis ETEM (thermos Scientific) operated at 300 kV in dual EELS mode with energy resolution of 1.3 eV. A time flight secondary ion mass spectrometer (TOF-SIMS; TESCAN Amber) was used in a dynamic mode to get a depth profile of Mn and Co elements in MnCoO_*x*_ sample.

X-ray photoelectron spectroscopy (XPS) was used to analysis the relative abundance and chemical state of the surface components of MnCoO_*x*_ catalysts (Thermo Fisher ESCALAB^TM^ xi^+^). The monochromatic Al *K*α was used as the photo source (1486 eV). Binding energies were corrected for surface charging by referring to C 1 s peak at 284.8 eV. For depth profile analysis, Ar^+^ sputtering was performed with an acceleration voltage of 500 eV with an irradiation area of 2 mm×2 mm. Also, the in-situ XPS analysis was conducted to investigate the evolution of MnCoO_*x*_ catalyst during ethane oxidation. XPSPEAK41 software was used to conduct peak deconvolution. The experimental peaks were decomposed though mixing Gaussian-Lorentzian functions (80%-20%) after Shirley background subtraction. The relative ratio of each element with different valence states was calculated based on the peak areas.

Both hydrogen temperature-programmed reduction (H_2_-TPR) and oxygen temperature-programmed desorption (O_2_-TPD) were performed on a Micromeritics AutoChem II 2920 analyzer equipped with a TCD detector. For H_2_-TPR measurement, about 100 mg sample was loaded and pretreated in He at 200 °C for 2 h. After cooling down, the analysis was conducted under 10%H_2_/Ar flow from 50 to 700 °C with a ramping rate of 10 °C min^-1^. Similar to H_2_-TPR tests, O_2_-TPD was conducted by firstly purging with He flow at 100 ^o^C for 30 min to remove moisture and subsequently, switch to 10%O_2_/He for 1 h. After that, the temperature was cooling down to room temperature in He flow to remove the physiosorbed O_2_ and stabilize the detector baseline. Eventually, the temperature was programmed from 50 to 800 ^o^C at a ramping rate of 10 ^o^C min^-1^ in He flow. Ethane temperature-programmed surface reduction (C_2_H_6_-TPSR) was carried out in a fixed-bed reactor with a mass spectrometer (MKS-Cirrus3, USA) to investigate the property of MnO_2_/MnCo_2_O_4_ interface. The catalyst was firstly pretreated by 10%O_2_/Ar flow (40 mL min^-1^) at 300 °C for 1 h. After cooling down, the inlet gas was switched to 10 vol%C_2_H_6_/He (50 mL min^-1^) for 30 min to stabilize the baseline. After that, a temperature-programmed reduction was conducted from 50 to 400 °C at a ramping rate of 10 °C min^-1^. The MS signals of C_2_H_6_ (*m/z* = 30) and CO_2_ (*m/z* = 44) were recorded accordingly. Ethane temperature-programmed desorption (C_2_H_6_-TPD) were performed on the same instrument. Typically, about 0.1 g of catalyst was pretreated under 10 vol%O_2_/Ar (40 mL min^-1^) flow at 300  ^o^C for 1 h to remove the surface adsorbed water. After cooling down to 50 °C, the sample was exposed to 10 vol%C_2_H_6_ for 30 min. Next, the inlet gas was switched to He flushed for another 30 min. After being saturated with C_2_H_6_, the temperature was subsequently ramped from 50 to 650 °C at a rate of 10 ^o^C min^-1^ in He flow (50 ml min^-1^). The desorbed C_2_H_6_ (*m/z* = 30), CO_2_ (*m/z* = 44), CO (*m/z *= 28), and H_2_O (*m/z* = 18) were monitored by the mass spectrometer. For C_2_H_6_-O_2_-TPSR and C_2_H_6_-O_2_ + H_2_O-TPSR experiments, the pretreated catalysts (ca. 0.1 g) were flushed with 10%C_2_H_6_/He (50 mL min^-1^) flow at 50 ^o^C for 1 h, followed by He (50 mL min^-1^) purging for 30 min. After that, the catalyst was heated from 50 to 400 ^o^C under O_2_ flow with and without H_2_O addition (10 vol% O_2_/N_2_, 5 vol% H_2_O, total flow rate 40 mL min^-1^).

CO chemisorption was used to determine the number of active sites (#CO, μmol g^-1^) on a Quantachrome ChemBETPulsar analyzer. Samples were purged with He at 100 °C for 30 min to remove moisture, and then reduced by 5%H_2_/Ar flow at 250 °C for 1 h. Afterwards, CO titration was performed by using thermal conductivity detector (TCD) as detector. The turnover frequency (TOF) defined as the number of alkane (CH_4_, C_2_H_6_, and C_3_H_8_) molecules converted per active site per second, was calculated based on the following equation.

1 $${TOF}=\frac{{F}_{{CnH}2n+2.{inlet}}\cdot {X}_{{CnH}2n+2}}{{{{{{\rm{\#}}}}}}{CO}\cdot M}$$

where F_C(n)H(2n+2), inlet_ represented the inlet flow rate (mol s^-1^) of C_n_H_2n+2_, *M* represents the molecular weight (g mol^-1^) of alkane.

In-situ diffuse reflectance infrared Fourier transform spectroscopy (in-situ DRIFTs) experiments were performed on a Thermo Nicolet iS50 spectrometer equipped with mercury cadmium telluride (MCT) detector. Prior to each experiment, the catalysts were pretreated at 300 °C in 10%O_2_/N_2_ (50 ml min^-1^) flow for 30 min and quickly cooling down to room temperature. After that, the experiment was performed under 1%C_2_H_6_/10%O_2_/89%N_2_ mixture to observe the evolution of reactants/products and intermediates. All the spectra were collected at a resolution of 16 cm^-1^ with 32 scans in the temperature range of 50–350 °C. In addition, we examined the adsorption behavior of ethane over MnCoO_x_-0.5 catalyst and references at 25 °C using the same setup. To investigate the interfacial property of MnCoO_*x*_ catalyst, DRIFT was coupled with MS (DRIFT-MS) to perform ethane oxidation at isotherm conditions without O_2_ feed (1%C_2_H_6_ balanced by He, 250 °C). The MS signals of products were collected as a function of time.

Steady-state isotopic labeling experiments were performed in a fixed-bed reactor. 0.1 g of catalyst was pretreated in air at 300 °C for 1 h with a gas flow rate of 50 mL min^-1^ and then flushed by N_2_ for 30 min to clean the adsorbed O_2_. After cooling down to 50 °C, the mixed gas of 1 vol%^18^O_2_ and C_2_H_6_ (1 ml min^-1^) balanced by He was introduced with a total gas flow rate of 50 ml min^-1^. After the baseline of MS signal was stabilized for 15-20 min, the reactor was heated from 50 to 350 °C at a ramping rate of 10 ^o^C min^-1^. During this process, the produced oxygen containing products (C^18^O_2_ (*m/z* = 48), C^16/18^O_2_ (*m/z* = 46), C^16^O_2_ (*m/z* = 44), H_2_^18^O (*m/z *= 20), H_2_^16^O (*m/z* = 18)) were monitored online by mass spectrometer. Transient mechanistic studies: Ethane oxidation was investigated in the temporal analysis of products (TAP) in pulse mode over MnCoO_*x*_ and bulk MnO_2_. Similar to steady-state isotopic labeling experiments, an oxidation treatment was conducted prior to the pulse experiments^18^O_2_:C_2_H_6_ = 1:1 mixture was pulsed in the temperature range of 200–400 ^o^C with a stepwise of 50 °C for MnCoO_*x*_ and 100 °C for MnO_2_.

### Catalytic reaction

Low-chain alkane combustion was carried out in a continuous flow packed bed reactor (*Φ* = 8 mm) to assess the catalytic activities of the MnCoO_x_ catalysts. 200 mg catalyst (40–60 mesh) was used for each activity test. The temperature was controlled by a K-type thermocouple. The reactant gas contains 3000 ppm C_2_H_6_ (CH_4_ or C_3_H_8_) balanced by air and N_2_ (O_2_:N_2_ = 11:89) at a flow rate of 200 ml min^-1^ (weight hourly space velocity (WHSV) = 60,000 h^-1^), accurately controlled by a gas distribution system with electric mass flow controllers (Brooks 5850 TR). The effluent was analyzed on-line by MKS-MultiGas analyzer. The range of test temperature was set at 50 to 400 ^o^C. Alkane conversion was calculated by the following equations:

2 $${X}_{{CnH}2n+2}\left(\%\right)=\frac{\left[{C}_{n}{H}_{2n+2}\right]{in}-\left[{C}_{n}{H}_{2n+2}\right]{out}}{\left[{C}_{n}{H}_{2n+2}\right]{in}}\times 100\%,\,n\ge 1$$

where [C_n_H_2n+2_]_in_ and [C_n_H_2n+2_]_out_ represented the inlet and outlet concentration of C_n_H_2n+2_, respectively; S_co2_ stand for CO_2_ selectivity; [CO], [CO_2_], and [C_n_H_2n_] represented the concentration of CO, CO_2_, and C_2_H_4_ (or C_3_H_6_), respectively. The reaction temperatures for 10%, 50%, and 90% conversion of C_n_H_2n+2_ to CO_2_ were assigned to T_10_, T_50_, and T_90_, respectively. Y_CO2_ stands for CO_2_ yield.

The surface area normalized rate (µmol_C2H6_ m^-2^ s^-1^) was calculated by the following equation:

3 $${r}_{C2H6=\frac{{X}_{C2H6}\cdot {F}_{C2H6}}{{S}_{{BET}}\cdot {m}_{{cat}.}}}$$

where *X*_C2H6_ (%) represents the conversion of C_2_H_6_, F_C2H6_ (mol s^-1^) is the mole flow rate of C_2_H_6_, S_BET_ (m^2^ g^-1^) is the surface area of tested materials, m_cat._(g) is the mass of the applied catalyst.

### Computational details

All calculations performed in this work were within the framework of Density Functional Theory (DFT) by using the Vienna Ab initio simulation program (VASP) 6.1.0. The projector-augmented wave (PAW) pseudopotentials were used to describe the electron-ion interactions^52^. The generalized gradient approximation with the Perdew-Burke-Ernzerhof functional (GGA-PBE) was used to treat the electron exchange and correlation energy^53^. Electron smearing was employed via Gaussian smearing method with a smearing width consistent to 0.05 eV. Valence electrons were described by a plane wave basis with an energy cutoff energy of 450 eV. Optimized structures were obtained by minimizing the forces on each atom using the conjugate gradient (CG) algorithm until <0.03 eV/Å. The energy convergence criteria were set to 10^-5 ^eV. A correction for Coulomb and exchange interactions was employed by setting U_eff_ = 3.5 eV and 3.1 eV (U_eff_ = coulomb U − exchange J) for Co and Mn atoms, respectively, using the model proposed by Dudarev et al.^54^. The D3 correction method (DFT-D3) was employed in order to include the van der Waals (vdW) interactions^55^.

The formation energy of an oxygen vacancy (E_Ov_) was calculated by the following equation:

4 $${{{{{{\rm{E}}}}}}}_{{{{{{\rm{ov}}}}}}}={{{{{{\rm{E}}}}}}}_{{{{{{\rm{slab}}}}}},{{{{{\rm{Ov}}}}}}}-{{{{{{\rm{E}}}}}}}_{{{{{{\rm{slab}}}}}}}+1/2\,{{{{{{\rm{E}}}}}}}_{{{{{{\rm{O}}}}}}2}$$

where E_slab,Ov_ is the energy of the defective MnCo_2_O_4_ slab surface, E_slab_ is the energy of the perfect slab surface, and E_O2_ is the energy of the gaseous oxygen molecule.

The adsorption energy of oxygen (E_ads,O2_) was calculated based on a perfect slab surface by the following equation:

5 $${{{{{{\rm{E}}}}}}}_{{{{{{\rm{ads}}}}}},{{{{{\rm{O}}}}}}2}={{{{{{\rm{E}}}}}}}_{{{{{{\rm{slab}}}}}},{{{{{\rm{O}}}}}}2}-{{{{{{\rm{E}}}}}}}_{{{{{{\rm{slab}}}}}}}-{{{{{{\rm{E}}}}}}}_{{{{{{\rm{O}}}}}}2}$$

where E_slab,O2_ is the energy of MnCo_2_O_4_ slab surface covered by the oxygen molecule, E_slab_ is the energy of the clean slab surface, and E_O2_ is the energy of the gaseous oxygen molecule.

The adsorption energy of ethane (E_ads,C2H6_) was calculated based on a perfect slab surface by the following equation:

6 $${{{{{{\rm{E}}}}}}}_{{{{{{\rm{ads}}}}}},{{{{{\rm{C}}}}}}2{{{{{\rm{H}}}}}}6}={{{{{{\rm{E}}}}}}}_{{{{{{\rm{slab}}}}}},{{{{{\rm{C}}}}}}2{{{{{\rm{H}}}}}}6}-{{{{{{\rm{E}}}}}}}_{{{{{{\rm{slab}}}}}}}-{{{{{{\rm{E}}}}}}}_{{{{{{\rm{C}}}}}}2{{{{{\rm{H}}}}}}6}$$

where E_slab,C2H6_ is the energy of MnCo_2_O_4_ slab surface covered by the ethane molecule, E_slab_ is the energy of the clean slab surface, and E_C2H6_ is the energy of the gaseous ethane molecule.

The calculated equation for the surface energy (*Ω*) of the three crystal facets can be expressed as follows:

7 $$\varOmega=1/2A[{G}_{{slab}}-N(O)\mu (O)-N({{{{{\rm{Mn}}}}}})\mu ({{{{{\rm{Mn}}}}}})-N({{{{{\rm{Co}}}}}})\mu ({{{{{\rm{Co}}}}}})]$$

where *G*_slab_ is approximated as the total energy calculated by DFT and A is the surface area of the crystal facet terminations. *N*(O), *N*(Mn) and *N*(Co) are the numbers of O, Mn and Co atoms on the crystal facets, and *μ*(O), *μ*(Mn) and *μ*(Co) are the chemical potentials of O, Mn and Co atoms. Since the chemical potentials of O, Mn and Co are assumed to be in equilibrium with the bulk MnCo_2_O_4_, they are related through the following expression:

8 $$\mu ({{{{{\rm{Mn}}}}}}{{{{{{\rm{Co}}}}}}}_{2}{{{{{{\rm{O}}}}}}}_{4})=\mu ({{{{{\rm{Mn}}}}}})+2\mu ({{{{{\rm{Co}}}}}})+4\mu (O)$$

where *μ*(MnCo_2_O_4_) is the chemical potential of the bulk MnCo_2_O_4_, which is approximated by the total energy of bulk MnCo_2_O_4_ unitary.

### Supplementary information

Supplementary Information

Peer Review File

### Source data

Source Data

## Data Availability

The data that generated in this study are provided in the published article and its Supplementary Information/Source Data files. The data that support the findings of this study have been deposited in the FigShare database under accession code 10.6084/m9.figshare.24150888. Source data are provided with this paper.

## Code availability

The data that generated in this study are provided in the published article and its Supplementary Information/Source Data file. The data that support the findings of this study have been deposited in the FigShare database under accession code 10.6084/m9.figshare.24150888. Source data are provided with this paper.
