# Supplementary material for: Redox-induced controllable engineering of MnO2-MnxCo3-xO4 interface to boost catalytic oxidation of ethane
Source: Nat Commun. 2024 May 15;15:4118. doi: 10.1038/s41467-024-48120-8 (PMC11096404; doi:10.1038/s41467-024-48120-8)
Supplement: Supplementary file 1 — Supplementary Information [file 41467_2024_48120_MOESM1_ESM.pdf]

Redox-induced Controllable Engineering of MnO_2-$Mn_xCo_{3-x}O_4$ Interface to Boost Catalytic Oxidation of Ethane

Haiyan Wang[a]+, Shuang Wang[a]+, Shida Liu[b]+,*, Yiling Dai[c], Zhenghao Jia[d], Xuejing Li[b], Shuhe Liu[b], Feixiong Dang[a], Kevin J. Smith[e], Xiaowa Nie[a]*, Shuandi Hou[b]*, Xinwen Guo[a]*

[a]State Key Laboratory of Fine Chemicals, Frontiers Science Center for Smart Materials, School of Chemical Engineering, Dalian University of Technology, Dalian 116024, P.R. China
[b]SINOPEC Dalian (Fushun) Research Institute of Petroleum and Petrochemicals. Dalian 116045, P.R. China
[c]Key Lab of Organic Optoelectronics & Molecular Engineering, Department of Chemistry, Tsinghua University, Beijing 100084, China.
[d]Division of Energy Research Resources, Dalian Institute of Chemical Physics, Chinese Academy of Sciences, 457 Zhongshan Road, Dalian, 116023, China.
[e]Department of Chemical and Biological Engineering, University of British Columbia, 2360 East Mall, Vancouver, B.C., V6T 1Z3, Canada.

#These authors contributed equally: Haiyan Wang, Shuang Wang, Shida Liu
*These authors jointly supervised this work: Shida Liu, Xiaowa Nie, Shuandi Hou, Xinwen Guo

Correspondence: Shida Liu (liushida.fshy@sinopec.com), Xiaowa Nie (niexiaowa@dlut.edu.cn), Shuandi Hou (houshuandi.fshy@sinopec.com), Xinwen Guo (guoxw@dlut.edu.cn)

Table of Contents

Table of Contents 2
Supplementary Figures..3
Supplementary Tables..42
Supplementary References 55

Supplementary Figures

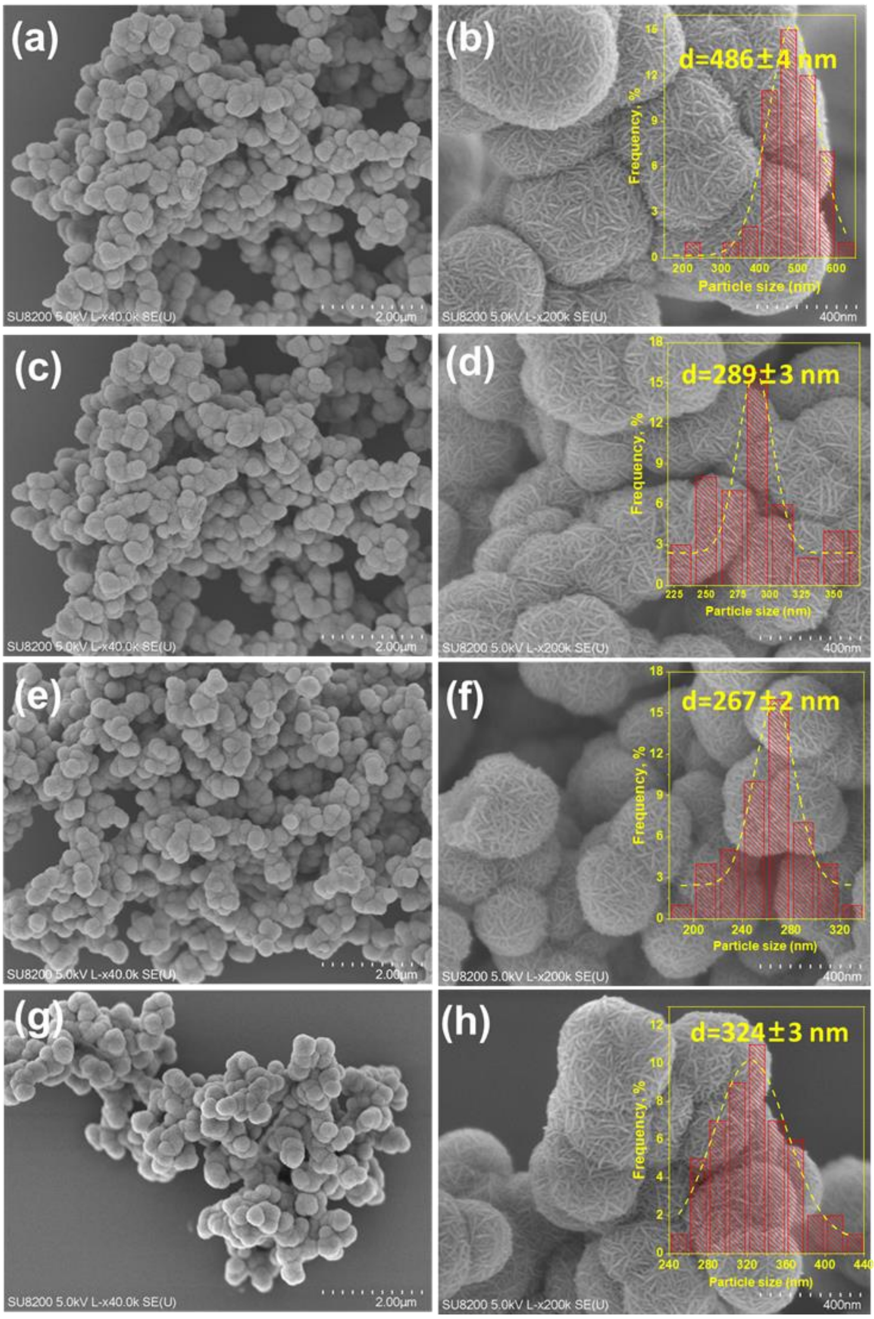

Supplementary Figure 1. SEM images of the as-synthesized $MnCoO_x$ catalysts with different Mn/Co ratios (a, b) Mn/Co=1:10, (c, d) Mn/Co=1:2, (e, f) Mn/Co=2:1, and (g, h) $MnCoO_x$-0.5 precursor (the inserts of supplementary Figure 1(b, d, f, h) represent the particle size distribution of $MnCoO_x$ catalysts).

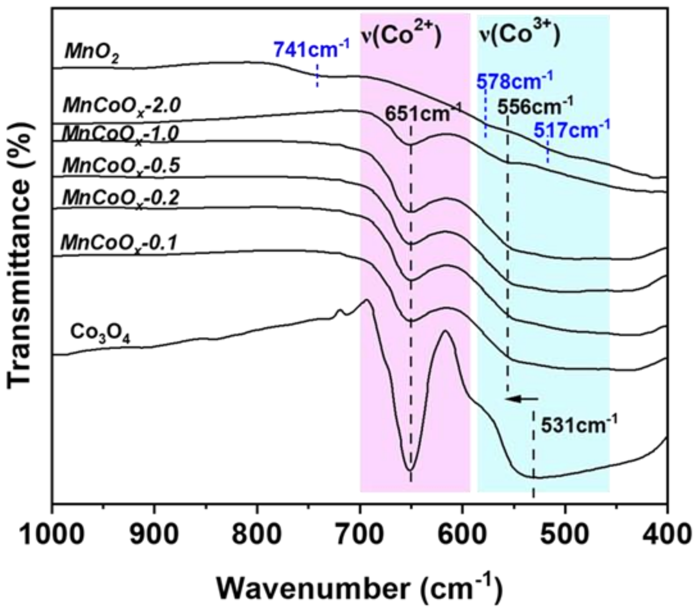

Supplementary Figure 2. ATR FT-IR spectra of synthesized $MnCoO_x$ catalysts (Red shading area: the vibration of Co^{2+} species; green shading area: the vibration of Co^{3+} species).

Supplementary Discussion: the Fourier-transformed infrared (FT-IR) analyses were performed to study the chemical structure of synthesized $MnCoO_x$ catalysts. As shown in Supplementary Figure 2, two strong IR absorption bands located at around 531 (the stretching vibration of Co^{3+}-O from octahedral sites) and 651 cm^{-1} (the stretching vibration of Co^{2+}-O from tetrahedral sites), were observed on Co_3O_4 reference.[1] The band centered at around 556 cm^{-1}, corresponds to the stretching vibration of Mn-O-Co bonds on the $MnCoO_x$ catalysts.[2] Compared to the fcc-Co_3O_4, the adsorption band of $MnCoO_x$ at Co^{3+} sites slightly shifted towards higher wavenumber, suggesting that part of the Mn^{2+} was oxidized to high valence Mn^{3+}.

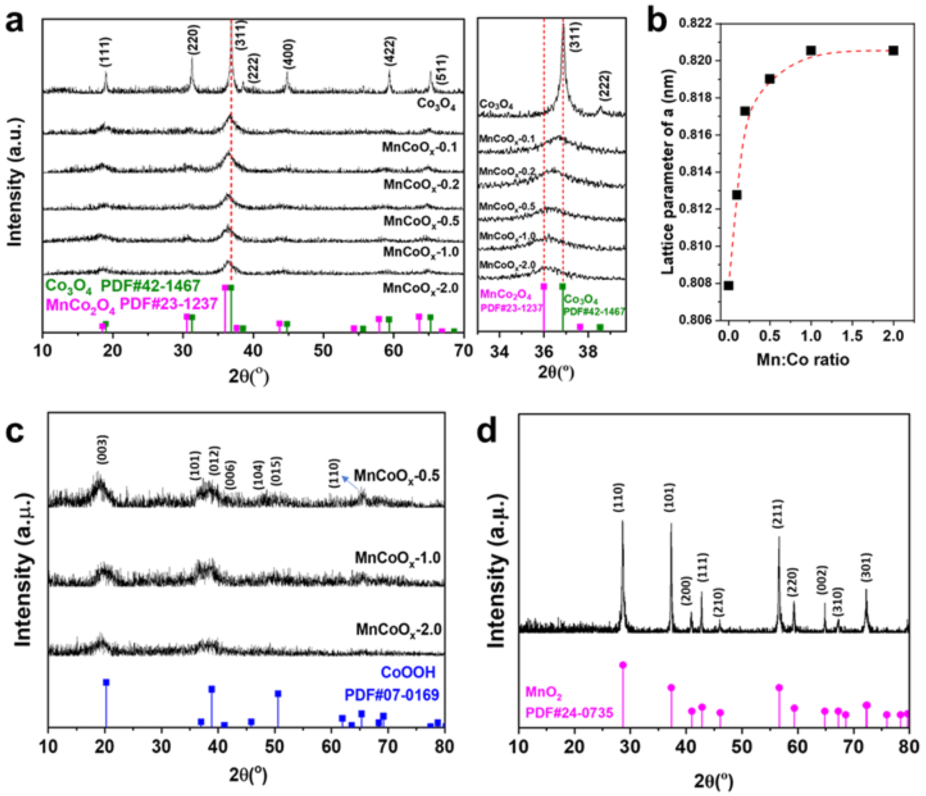

Supplementary Figure 3. XRD patterns of (a) $MnCoO_x$ precursors (CoOOH PDF card#:07-0169)) and (b) MnO_2 reference (PDF card#: 24-0735).

Supplementary Discussion: Supplementary Figure 3(a) showed the XRD pattern of $MnCoO_x$ and Co_3O_4 reference. Obviously, the peak intensities of $MnCoO_x$ catalysts were broader and weaker compared to the reference Co_3O_4. Moreover, the diffraction pattern slightly shifted towards low diffraction angle as Mn/Co ratio increased, up to the stoichiometric Mn/Co ratio (0.5) of $MnCo_2O_4$ spinel. No obvious peak shift was observed above Mn/Co ratio of 0.5. The observed structural evolution implied the modification of Co_3O_4 by Mn ions, resulting in the formation of $MnCo_2O_4$ spinel (PDF#23-1237) through expanding the unit cell of Co_3O_4 (PDF#42-1467). Here, the lattice parameters were calculated and reported in Supplementary Table 1, which increased monotonically with an increasing Mn/Co ratio and a levelling-off above 0.5 (Supplementary Figure 3(b)). No MnO_2 phases were detected even when the Mn/Co ratio exceeded 0.5, perhaps due to their well distribution and low crystallinity. The obtained XRD data were in accordance with the Raman results, suggesting that the overall synthesis was well controlled by a moderate redox chemistry. The formation of CoOOH (PDF#07-0169) was identified (Supplementary Figure 3(c)), with diffraction peaks at 2θ = 20.2°, 36.9°, 38.8°, 50.6°, 65.3°, and 68.4°, respectively. No bulk phase of MnO_2 presented, indicating that Mn was uniformly distributed into the matrix of Co precursor and remained at amorphous states. Also, it is noted that the intensities of CoOOH reflections increased with decreased Mn/Co ratio. Supplementary Figure 3(d) presents the XRD pattern of MnO_2 reference.

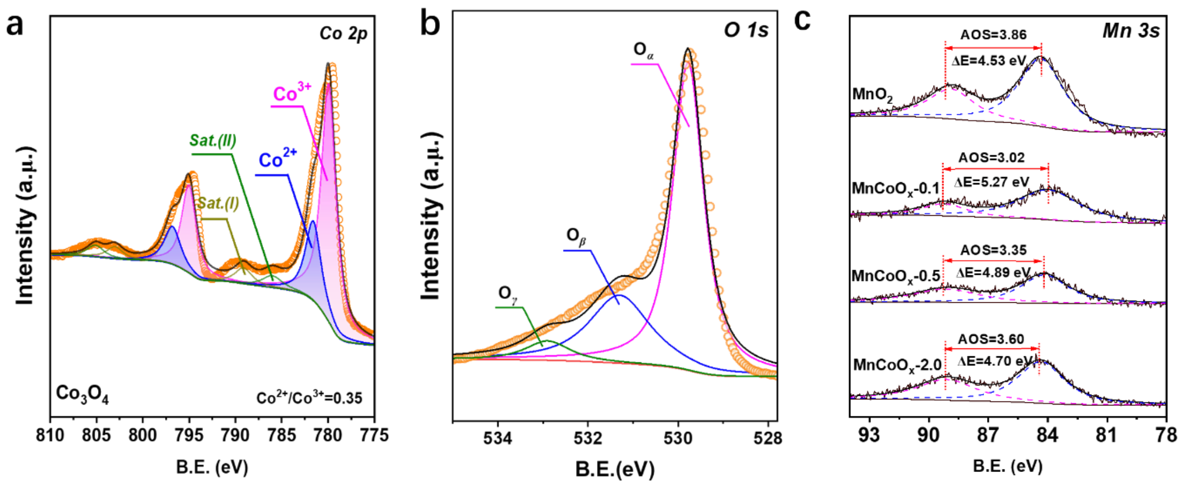

Supplementary Figure 4. XPS spectra of Co_3O_4 reference: (a) Co 2p; (b) O 1s; (c) Mn 3s.

Supplementary Discussion: As displayed in Supplementary Figure 4(c), the average oxidation state (AOS) of Mn was calculated based on an empirical equation (AOS = 8.956 - 1.126 x ΔE)[3] from measuring the energy differences (ΔE) from Mn 3s spectra. The AOS increased with increasing the Mn/Co ratio, which is consistent with Mn 2p results.

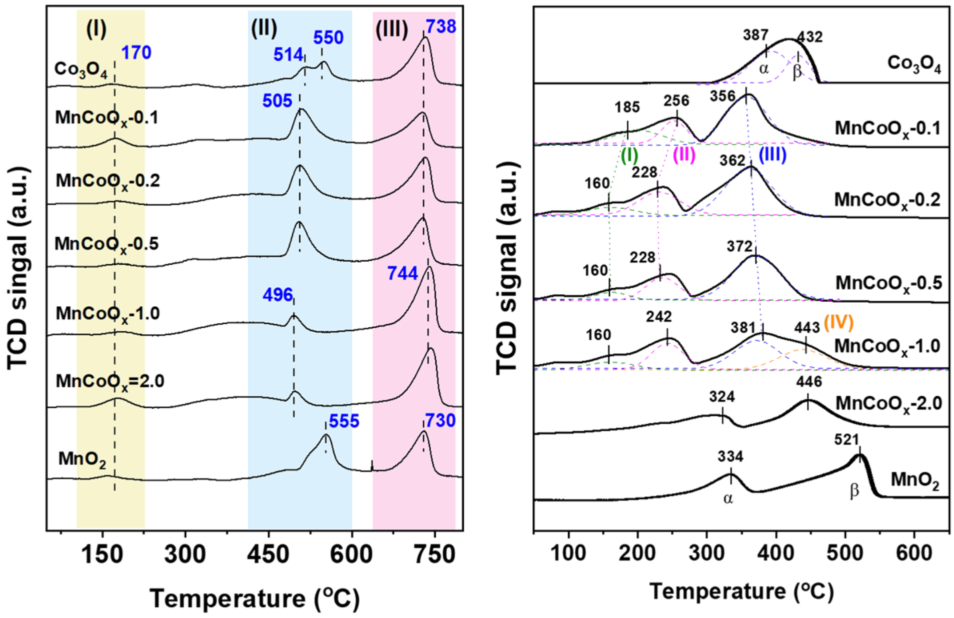

Supplementary Figure 5. Surface chemical states of $MnCoO_x$ catalysts and references. (a) O_2-TPD. (b) H_2-TPR profiles.

Supplementary Discussion: Clearly, three desorption peaks appeared in the temperature range of 50-800 °C, as shown in Supplementary Figure 5(a). The low-temperature desorption peak (<300 °C) corresponds to the surface adsorbed oxygen, including O^{2-} and O^{-} (Region I, yellow box). The medium-temperature desorption peak (300-600 °C) is ascribed to the surface and subsurface lattice oxygen (Region II, blue box). The high-temperature desorption peak (> 600 °C) is assigned to the bulk phase oxygen (Region III, pink box). Compared to the bulk phase O, the O appeared in Regions I &II are more active and tend to participate in the oxidation reactions.[4]

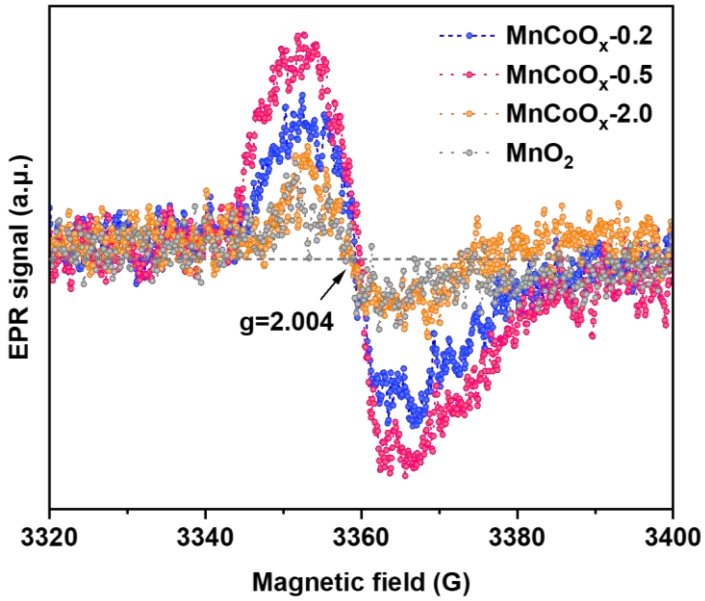

Supplementary Figure 6. (a) EPR spectra of $MnCoO_x$ catalysts and bulk MnO_2 reference recorded at 25 ^oC; (b) A correlation of cumulative area under H_2 reduction peaks (I & II & III) and O_2 desorption peaks (I & II).

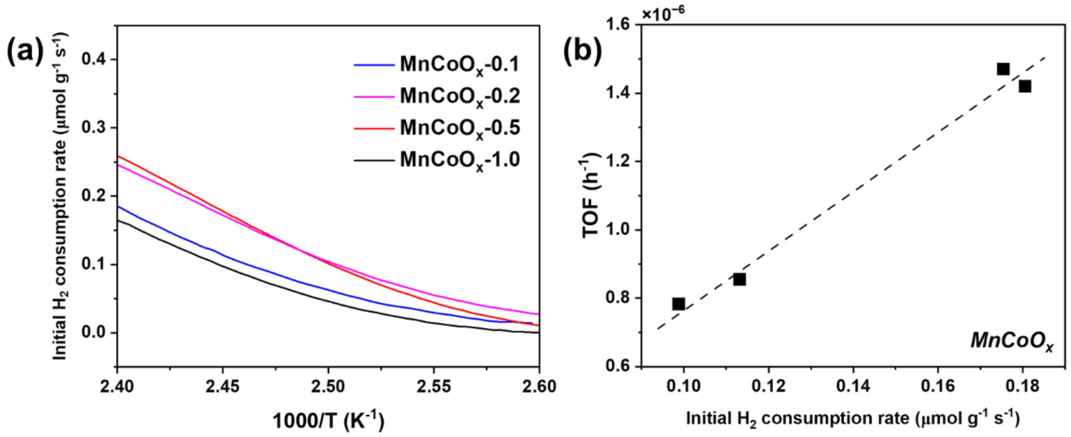

Supplementary Figure 7. (a) Initial H_2 consumption rate as a function of inversed temperature; (b) Dependence of ethane TOF (200 ^oC) on the initial H_2 consumption rate over $MnCoO_x$ catalysts.

Supplementary Note: The initial H_2 consumption rate was calculated to estimate the low-temperature reducibility of prepared $MnCoO_x$ catalysts.

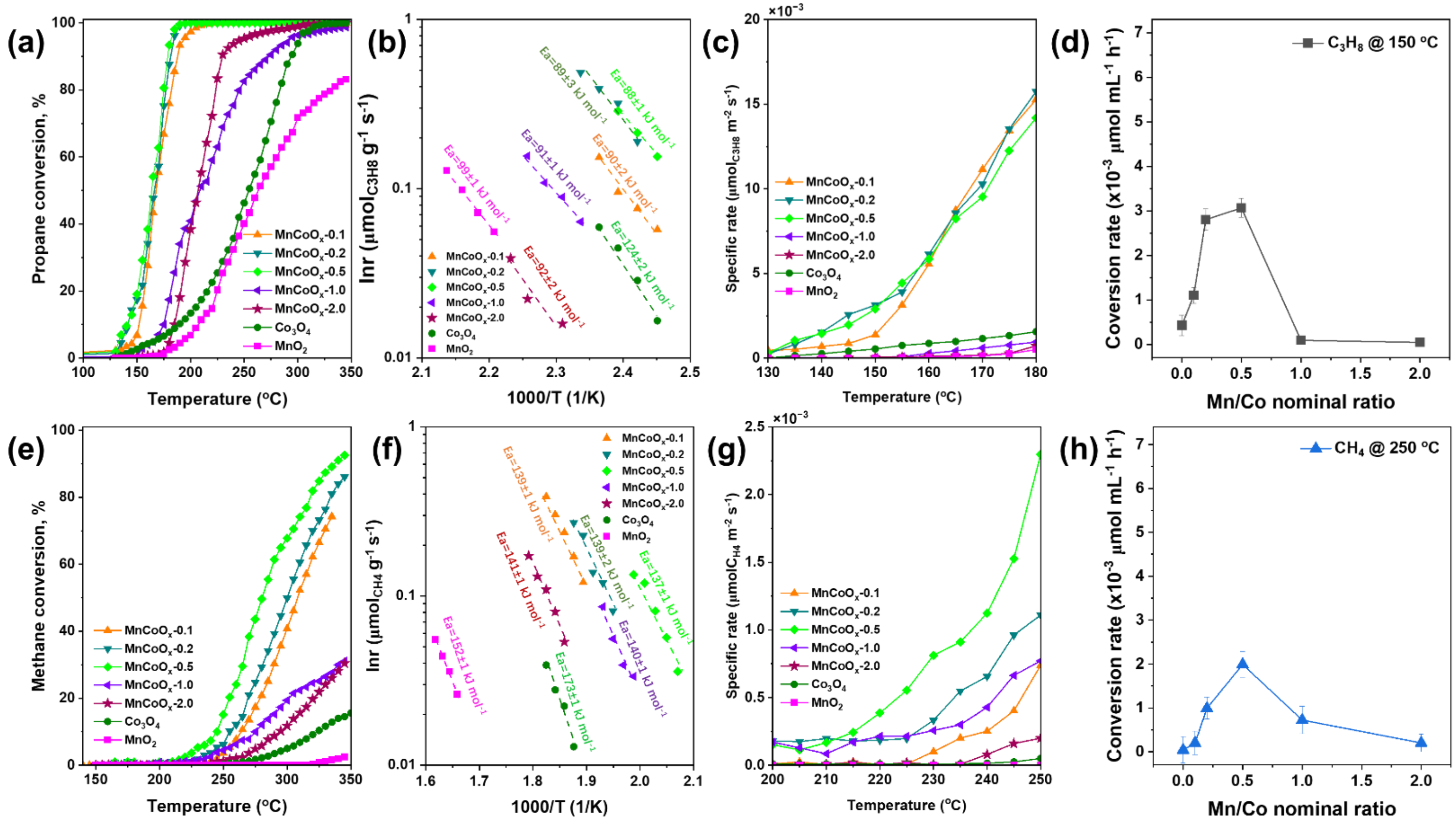

Supplementary Figure 8. Catalytic performance of $MnCoO_x$ catalysts in propane and methane oxidation: (a) propane conversion of the as-prepared catalysts (reaction conditions: *ca.* 200 mg catalyst, $[C_3H_8]$ = 3000 ppm, Q = 200 mL min^{-1} and WHSV = 60,000 h^{-1}); (b) Arrhenius plots; (c) specific reaction rate; (d) propane conversion rate as a function of Mn/CO nominal ratio at 150 °C (The error bar represents the standard deviation of the calculated conversion rate); (e) methane conversion of the as-prepared catalysts (reaction conditions: *ca.* 200 mg catalyst, $[CH_4]$ = 3000 ppm, Q = 200 mL min^{-1} and WHSV = 60,000 h^{-1}); (f) Arrhenius plots; (g) specific reaction rate; (h) methane conversion rate as a function of Mn/CO nominal ratio at 250 °C (The error bar represents the standard deviation of the calculated conversion rates).

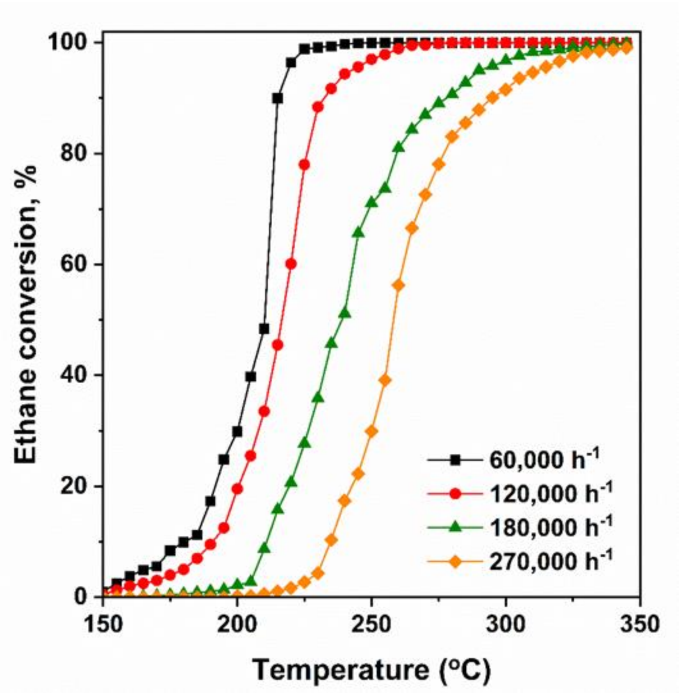

Supplementary Figure 9. Effect of WHSV on ethane oxidation over $MnCoO_x$-0.5 catalyst. (Reaction conditions: ca. 200 mg catalyst, [C_2H_6] = 3000 ppm, balanced by Air)

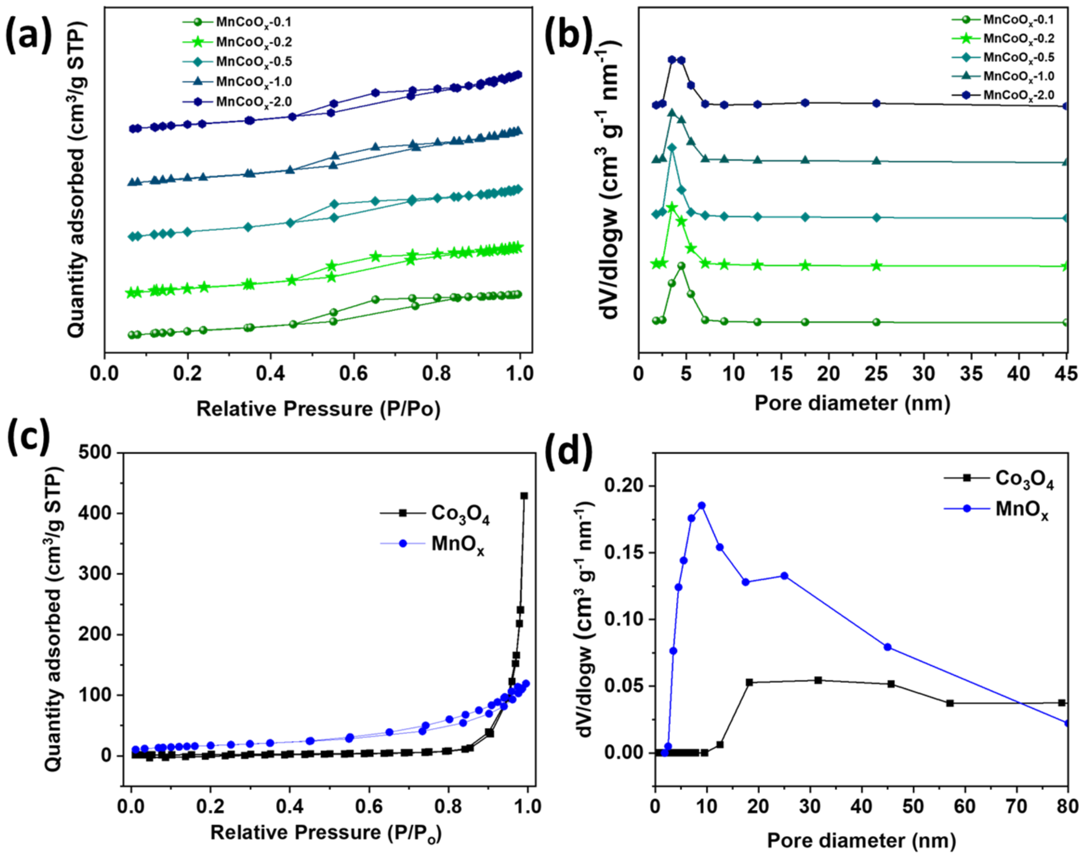

Supplementary Figure 10. N_2 adsorption-desorption isotherms and pore size distribution of the as-prepared $MnCoO_x$ catalysts (a, b) and references (c, d).

Supplementary Discussion: the physical properties of $MnCoO_x$ were examined by the N_2 adsorption-desorption isotherms (Supplementary Figure 10 and Table 7). The surface area of $MnCoO_x$ is in the range of 110-140 $m^2\ g^{-1}$ which is significantly higher than that of the monometallic Co_3O_4 and MnO_2, and the pore volume is about 0.2 $cm^3\ g^{-1}$.

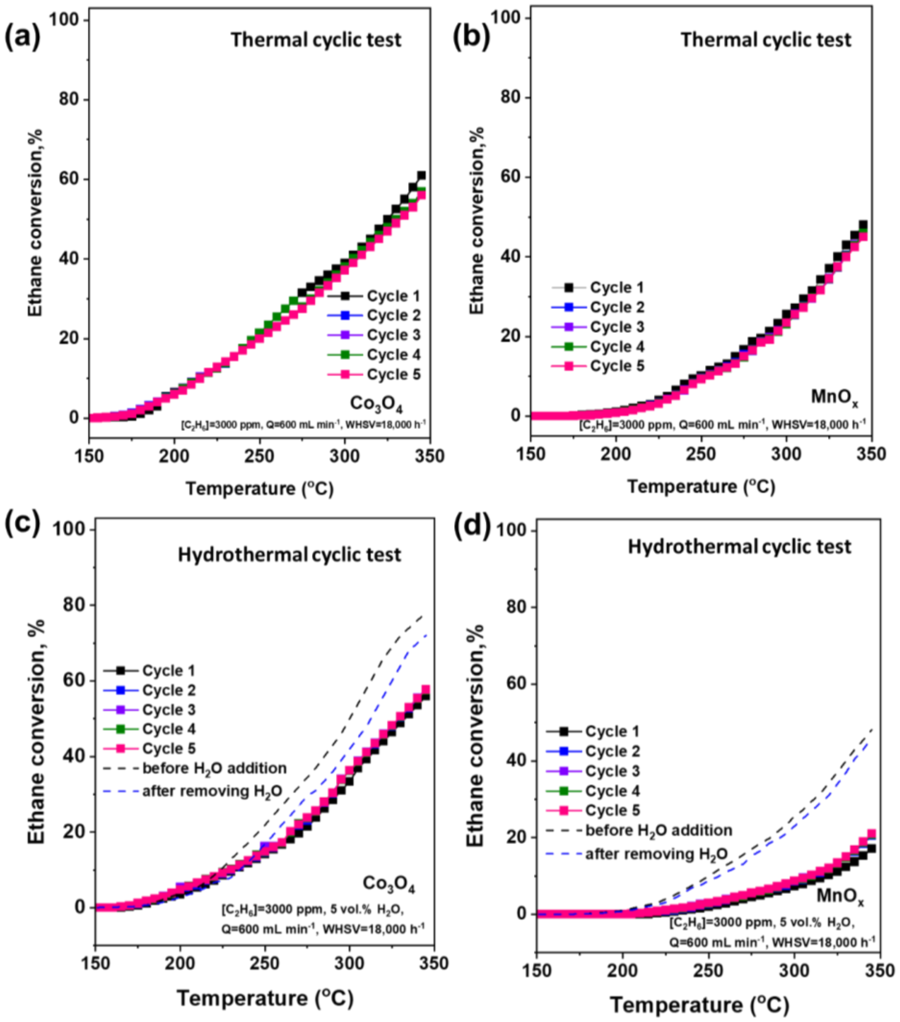

Supplementary Figure 11. Thermal and hydrothermal cyclic stability tests of Co_3O_4 (a, c) and MnO_2 (b, d) references (reaction conditions: ca. 200 mg catalyst, $[C_2H_6]$ = 3000 ppm, Q = 600 mL min^{-1} and WHSV = 180,000 h^{-1})

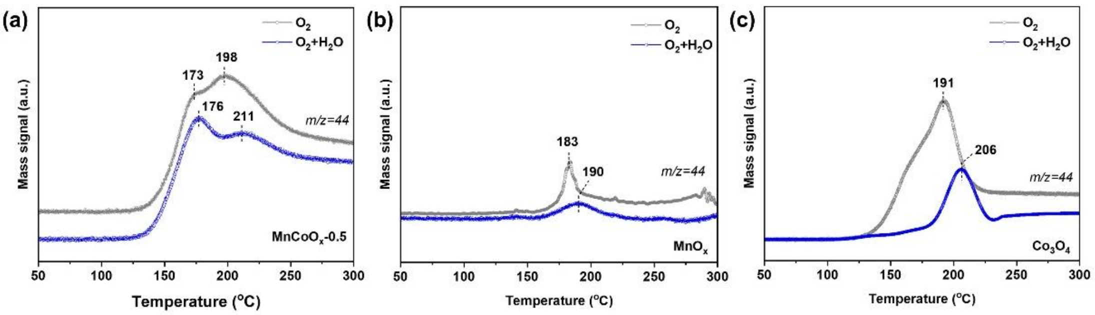

Supplementary Figure 12. Comparation of CO_2 signal (*m/z*=44) for $MnCoO_x$-0.5 (a), MnO_2 (b), and Co_3O_4 (c) catalysts during C_2H_6-O_2/O_2+H_2O TPSR under dry and humid conditions (10%O_2 and 10%O_2+5 vol% H_2O).

Supplementary Discussion: As shown in Supplementary Figure 12, C_2H_6-O_2/O_2+H_2O TPSR experiments were carried out to study the effect of water vapor by introducing O_2 or O_2+H_2O to C_2H_6 pre-adsorbed catalysts. It was found that only CO_2 (*m/z*=44) was detected both in the absence or presence of H_2O, indicating the occurrence of complete ethane oxidation over these materials. The amount of produced CO_2 decreased in the order of $MnCoO_x$-0.5 < Co_3O_4 < MnO_2, which is consistent with the increased reaction temperature of ethane conversion as reported in Supplementary Figure 11. The peak intensity of produced CO_2 significantly dropped after water addition over MnO_2 and Co_3O_4 references, while the decrease was negligible on $MnCoO_x$-0.5 catalyst. Also, it is noticeable that the temperature of C_2H_6 consumption peak became higher under humid conditions than dry conditions over MnO_2 and Co_3O_4 references. The CO_2 peak temperature decreased from 183 to 190 °C over MnO_2, and from 191 to 206 °C over Co_3O_4, respectively. This result suggested that the water vapor could inevitably occupy the active sites of MnO_2 and Co_3O_4, and then inhibite the activation of ethane, therefore a high temperature is required under water-containing conditions. Meanwhile, a significant drop on the produced CO_2 amount was observed over MnO_2 and Co_3O_4 catalysts. For $MnCoO_x$-0.5 catalyst, the C_2H_6 consumption peak at low temperature (173 °C) almost remained with no change on peak intensity, while the high-temperature C_2H_6 consumption peak slightly shifted from 198 to 211 °C along with a slightly decreased on peak intensity.

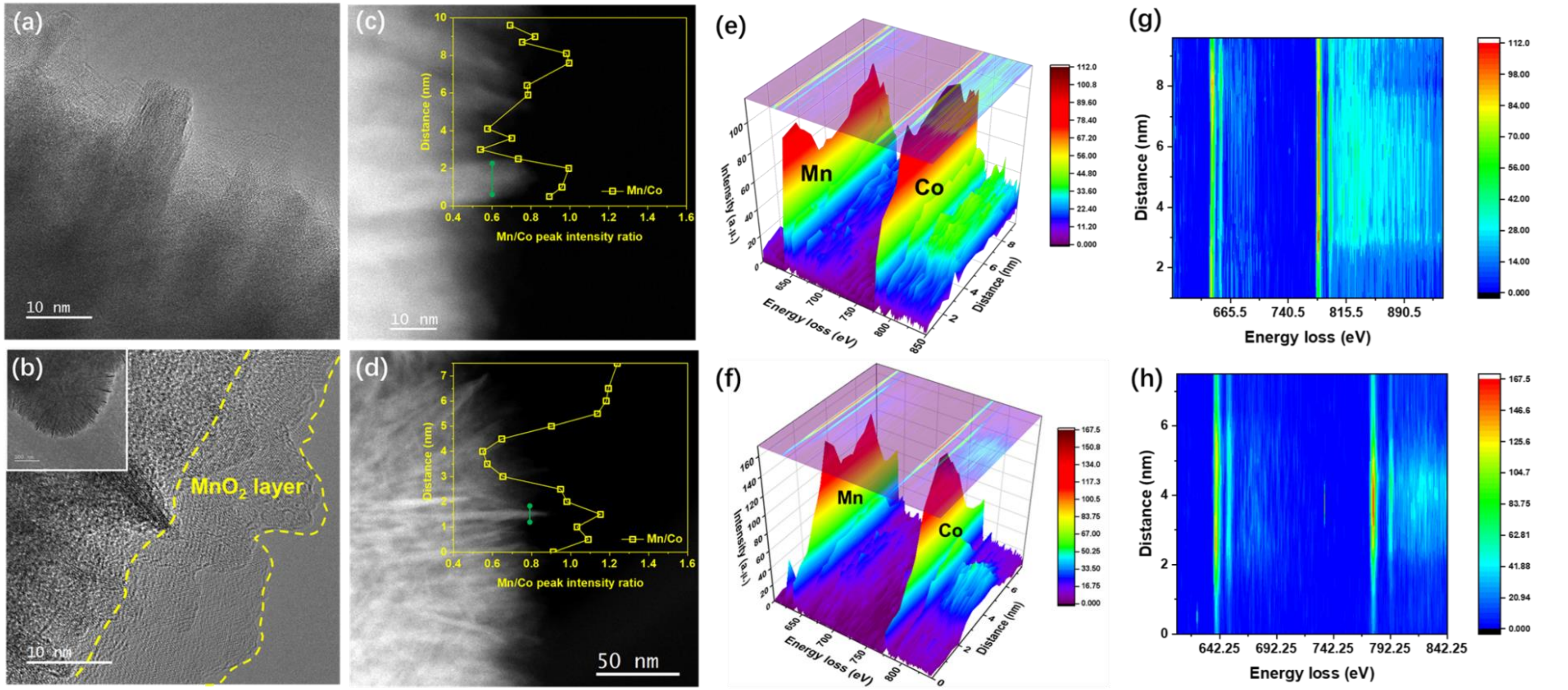

Supplementary Figure 13. HRTEM image of $MnCoO_x$-0.1 (a) and $MnCoO_x$-2.0 (b); HAADF image of $MnCoO_x$-0.1 (c) and $MnCoO_x$-2.0 (d); (e, g) EELS elemental line scanning of Mn and Co over $MnCoO_x$-0.1 (e, g) and $MnCoO_x$-2.0 (f, h) (The line scanning was performed based on the green line indicated on Figure S11(c) and (d), respectively).

Supplementary Discussion: for the $MnCoO_x$-0.1 catalyst, the MnO_2 boundary layer was not fully developed. Taking $MnCoO_x$-2.0 as an example, it was found that the thickness of MnO_2 layer significantly increased to ≥10 nm, with an uneven distribution. Therefore, varying Mn/Co ratio turns to be an effective way of tuning the coverage and thickness of MnO_2 layer and consequently, influencing the number of interfacial MnO_2-$MnCo_2O_4$ sites.

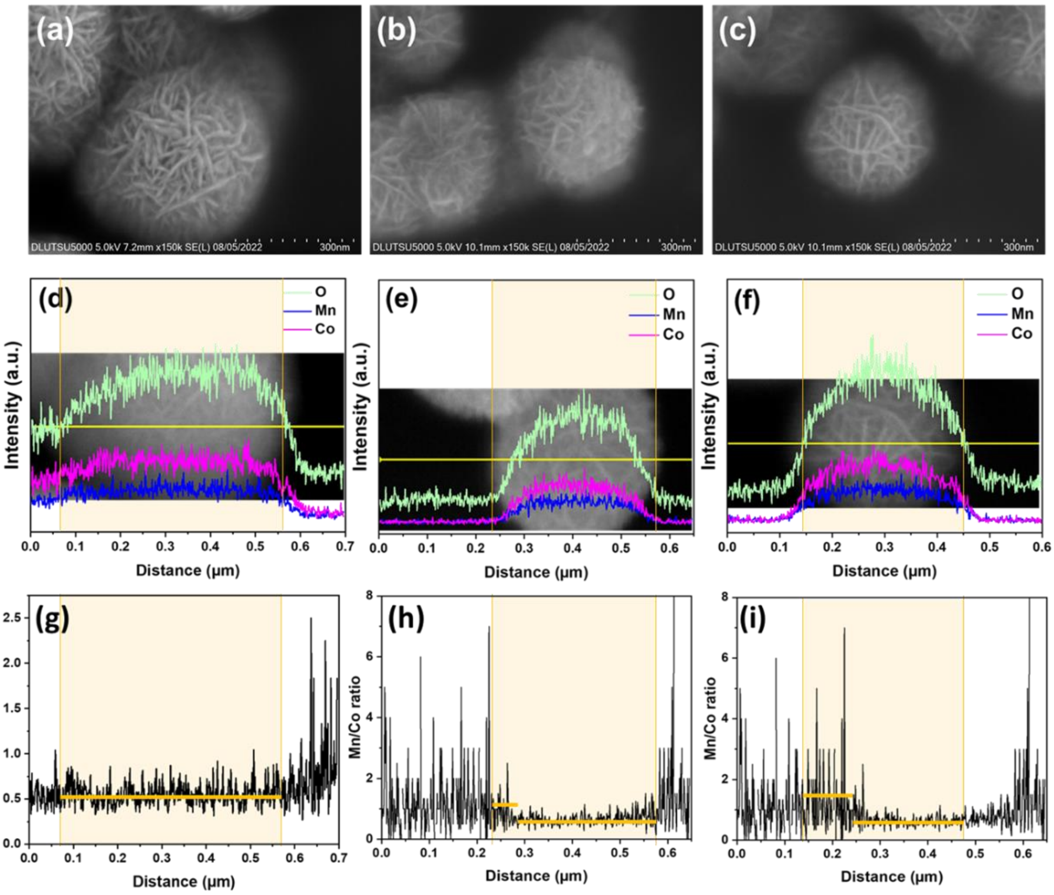

Supplementary Figure 14. SEM images and the corresponding EDX line scanning of $MnCoO_x$ microsphere: (a, d, g) $MnCoO_x$-0.1; (b, e, h) $MnCoO_x$-0.5; (c, f, i) $MnCoO_x$-2.0.

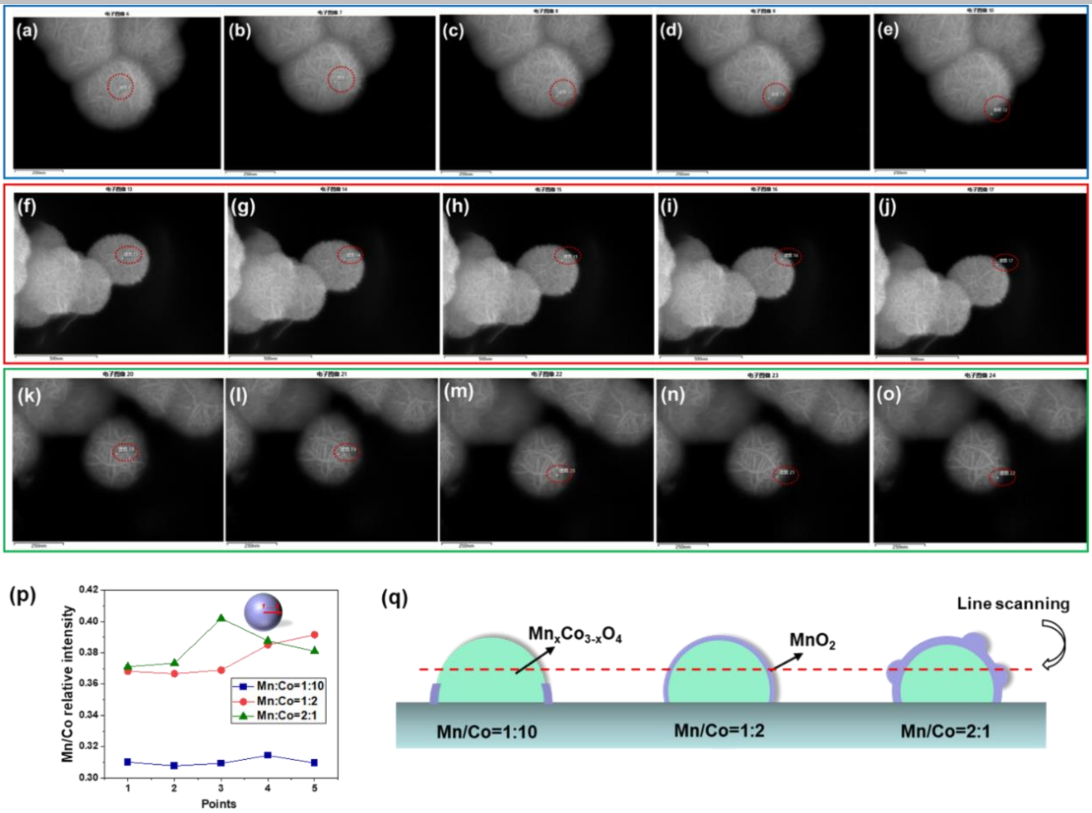

Supplementary Figure 15. SEM images and the corresponding multi-spot EDX elemental scanning as a function of the distance from particle center to the edge: (a-e) $MnCoO_x$-0.1; (f-j) $MnCoO_x$-0.5; (k-o) $MnCoO_x$-2.0. (p) the correlation between Mn/Co ratio and particle diameter. (q) schematic illustration of $MnCoO_x$ morphology change as a function of Mn/Co ratios.

Supplementary Discussion: Multi-spot EDX elemental scanning was employed from particle center to the edge as shown in Supplementary Figs. 15(a-o). The relative concentration of Mn/Co was plotted against particle radius to show concentration gradient change (Supplementary Figure 15p). The elemental analysis showed that the Mn/Co ratio was almost constant for $MnCoO_x$-0.1 catalyst. While a slight increase on Mn/Co ratio was observed from Point 1 to Point 5 on $MnCoO_x$-0.5, indicating that a small portion of Mn was concentrated at the edge of the micro-sphere as MnO_2 layer. However, the Mn/Co ratio showed a volcano-typed shape on $MnCoO_x$-2.0 catalyst, which may result from the uneven distribution of MnO_2 domains formed by excessive Mn addition. A schematic illustration was present in Supplementary Fig. 15q to explain the elemental distribution on $MnCoO_x$ surface.

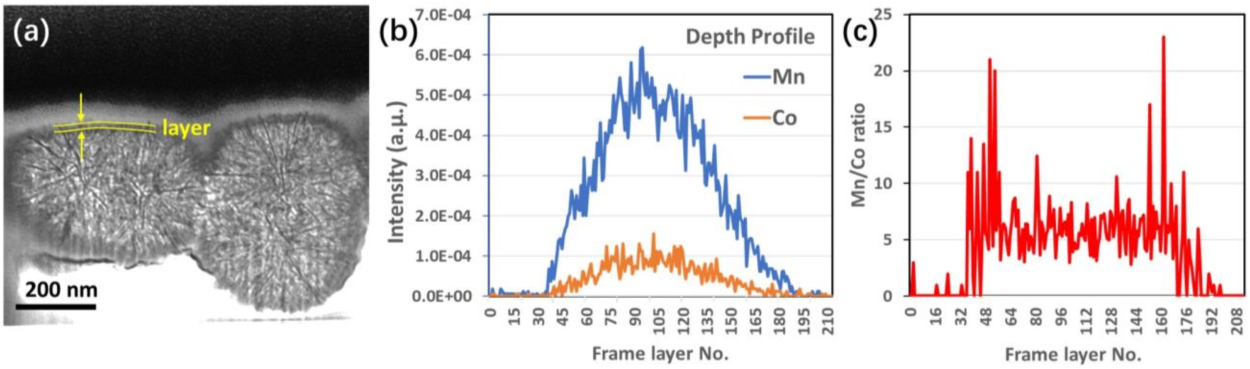

Supplementary Figure 16. (a) HAADF-STEM graph of $MnCoO_x$-1.0 catalyst; (b) Elemental distribution of Mn and Co by TOF-SIMS analysis; (c) Depth profile of Mn/Co ratio by TOF-SIMS analysis.

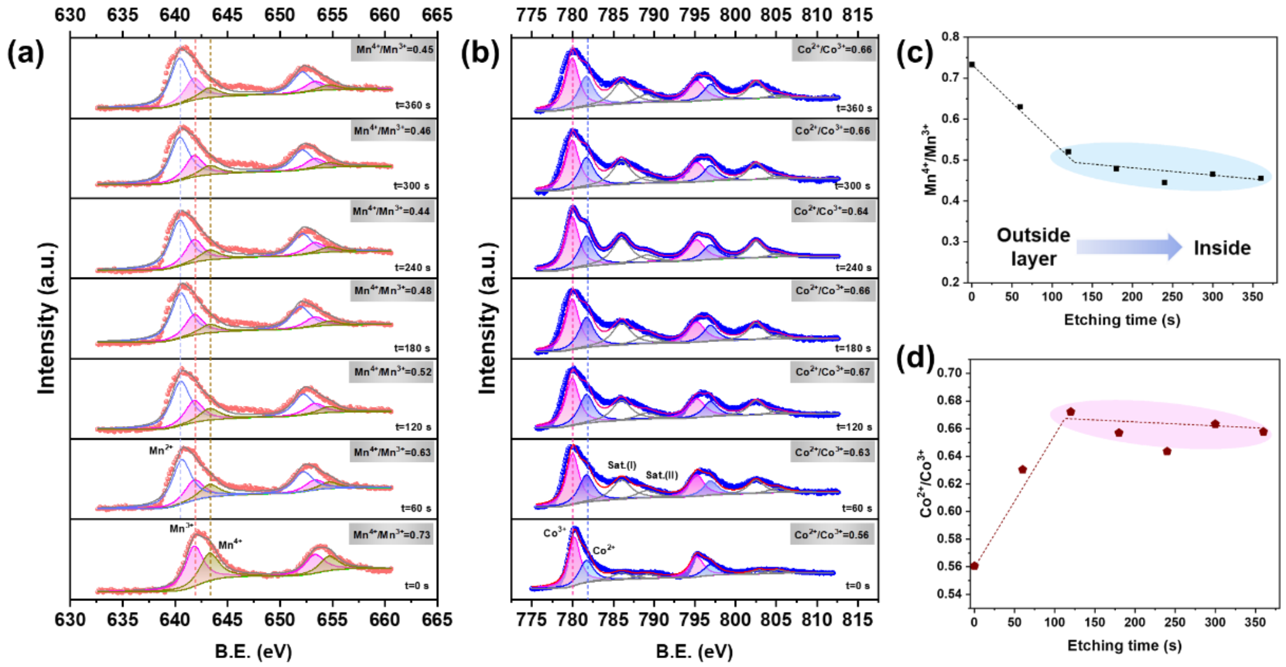

Supplementary Figure 17. XPS depth profile analysis of $MnCoO_x$-0.5 catalyst. (a) Mn^{4+}/Mn^{3+}; (b) Co^{2+}/Co^{3+}; (c) the Mn^{4+}/Mn^{3+} ratio as a function of etching time; (d) the Co^{2+}/Co^{3+} ratio as a function of etching time.

Supplementary Discussion: To get more insights of the $MnO_2/MnCo_2O_4$ interface, we performed a depth profile analysis by XPS on $MnCoO_x$ catalyst. Apparently, the ratio of Mn^{4+}/Mn^{3+} dramatically decreased with increasing etching time in the first 60 s (equivalent to 60 s x 0.08 nm/s = 4.8 nm), and reached to a relatively stable state afterwards. Accordingly, the ratio of Co^{2+}/Co^{3+} slowly increased with increased etching time in the first 60 s. Overall, the XPS analysis suggest that there are more Mn^{4+} species (MnO_2) retain on the surface of $MnCoO_x$ catalyst. The thickness of MnO_2 is about 4.8 nm, which is consistent with what we observed from HRTEM images (Fig. 3b).

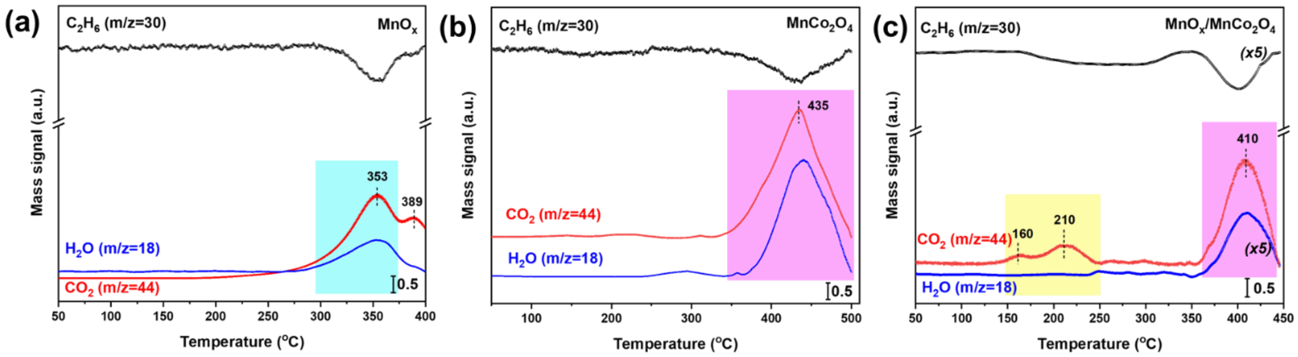

Supplementary Figure 18. C_2H_6-TPSR-MS profile of (a) MnO_2; (b) $MnCo_2O_4$, and (c) $MnO_2/MnCo_2O_4$.

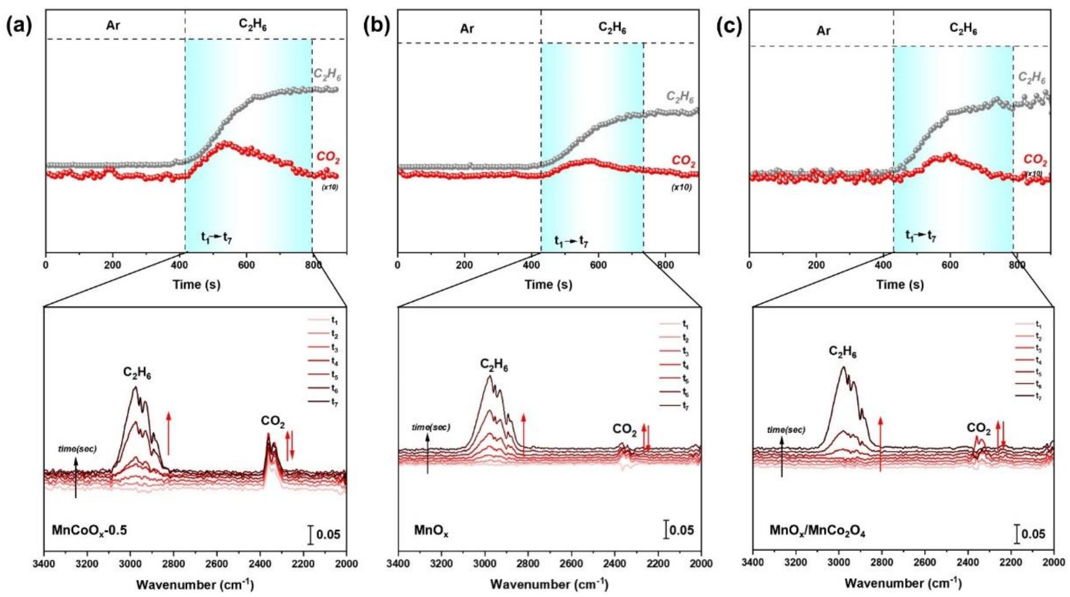

Supplementary Figure 19. DRIFT-MS analysis of isotherm ethane oxidation without O_2 at 250 ºC (a) $MnCoO_x$-0.5, (b) MnO_2, and (c) $MnO_2/MnCo_2O_4$ catalysts.

Supplementary Discussion: Clearly, CO_2 was detected once C_2H_6 was introduced, indicating that the surface lattice oxygen could easily participate in ethane oxidation. The integrated peak area of evolved CO_2 was obviously higher on $MnCoO_x$-0.5 catalyst compared to MnO_2 and $MnO_2/MnCo_2O_4$ references due the existence of MnO_2-$MnCo_2O_4$ interfacial sites. This result agrees with the C_2H_6-TPSR-MS measurement. Consistent with the MS signal change, the intensity of CO_2 peak observed from DRIFT spectra showed a gradual increase at the initial stage of C_2H_6 injection, and slowly decreased during the transition period due to the depletion of active lattice O.

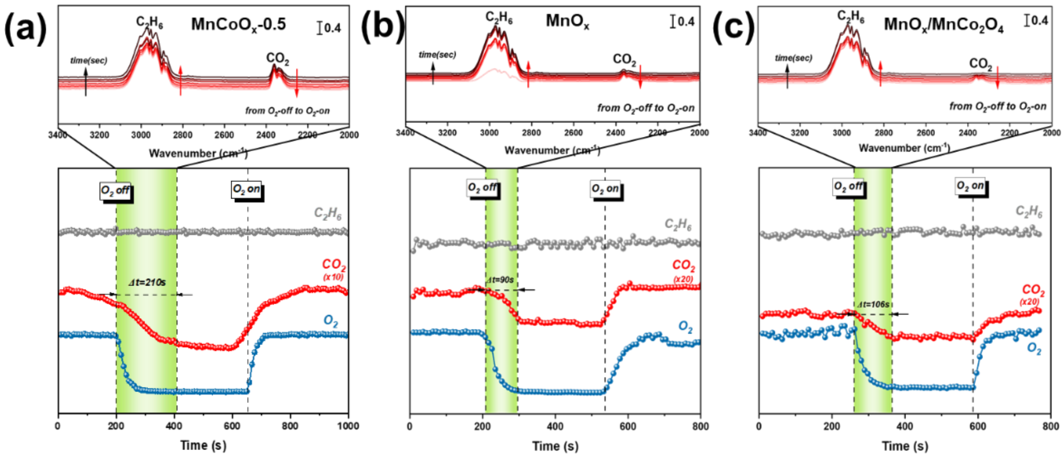

Supplementary Figure 20. Transient DRIFT-MS experiment with O_2 cut-off and turn-on 250 ºC. (a) $MnCoO_x$-0.5, (b) MnO_2, and (c) $MnO_2/MnCo_2O_4$ catalysts.

Supplementary Discussion: The redox properties of $MnCoO_x$-0.5 was studied by the designed transient time-resolved DRIFT-MS experiments at 250 ºC with O_2 cut-off/turn-on (Supplementary Fig. 20). After the system stabilized for 200 s, the O_2 was removed from the feed to reduce the catalyst by ethane and then added again to allow the catalyst re-oxidized under reaction conditions. As shown in Supplementary Fig. 19a, $MnCoO_x$-0.5 was gradually reduced as reflected by the formation of CO_2 from the depletion of active lattice O. The transition period followed the order of $MnCoO_x$-0.5 (Δt = 210 s) > $MnO_2/MnCo_2O_4$ (Δt = 106 s) > MnO_2 (Δt = 90 s), which is in accordance with the C_2H_6 isotherm experiments (Supplementary Fig. 19).

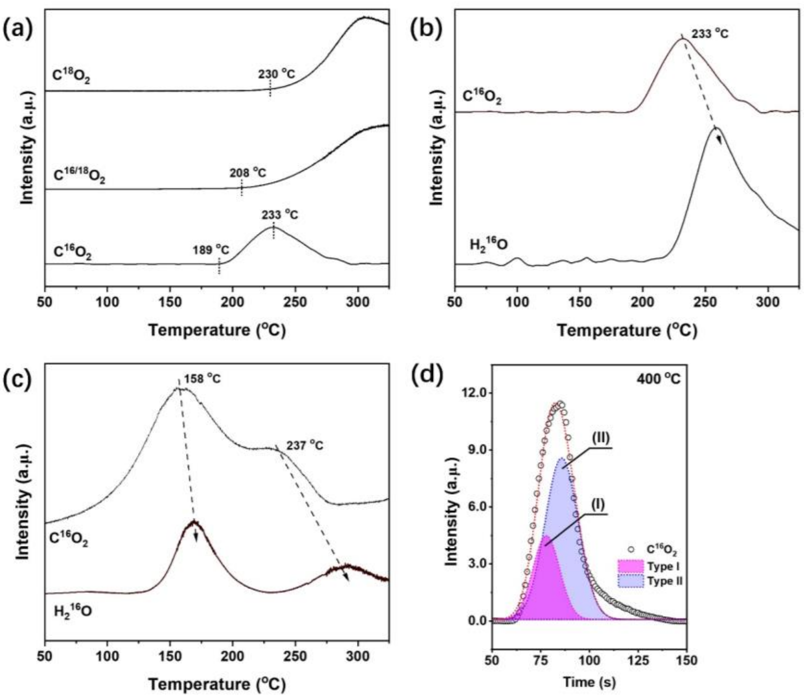

Supplementary Figure S21. ^{18}O isotopic labeling experiment in the temperature programmed oxidation of ethane over (a, b) bulk MnO_2 and (c) $MnCoO_x$-0.5. (d) peak deconvolution of $C^{16}O_2$ over bulk $MnCoO_x$-0.5 in transient experiment.

Supplementary Note: The production of $H_2{}^{16}O$ is slightly slower than the decrease of $C^{16}O_2$ due to the longer holdup of moisture in the catalyst bed.

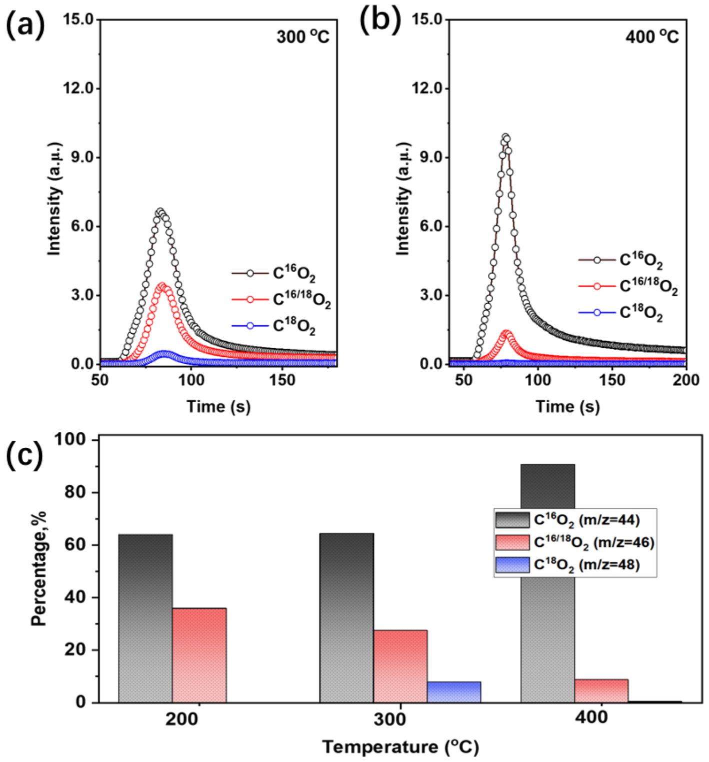

Supplementary Figure 22. Temporal analysis of products (TAP) of ethane oxidation over MnO_2 as a function of reaction temperature. (a) 300 ºC, (b) 400 ºC, (c) products distribution of CO_2 (including $C^{16}O_2$, $C^{16/18}O_2$, and $C^{18}O_2$).

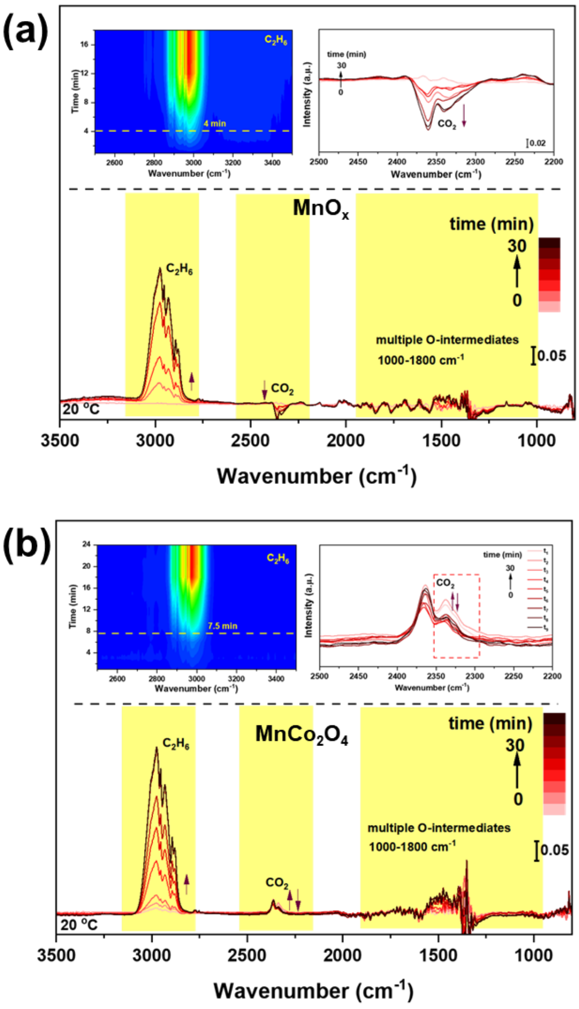

Supplementary Figure 23. Time-resolved DRIFT spectra of C_2H_6 adsorption on (a) MnO_2 and (b) $MnCo_2O_4$ catalysts.

Supplementary Discussion: The adsorption of ethane over MnO_2 and $MnCo_2O_4$ references was measured as a function of time by DRIFTs at 25 ^oC. The CO_2 signal increased to a maximum level and then decreased on $MnCo_2O_4$, suggesting the depletion of lattice O. A quick depletion of lattice O is noticed on MnO_2 as indicated by the decreased CO_2 signal once injecting ethane. Distinguished from these two references, the intensity of CO_2 was increased while increasing the adsorption time over $MnCoO_x$-0.5, indicating that there are more active O involved on $MnCoO_x$-0.5 (Fig. 5b).

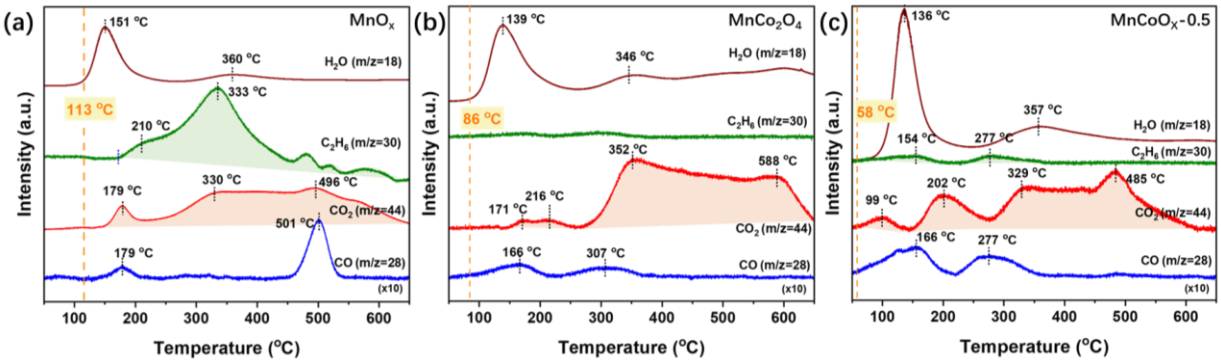

Supplementary Figure 24. C_2H_6-TPD. (a) MnO_2; (b) $MnCo_2O_4$; (c) $MnCoO_x$-0.5.

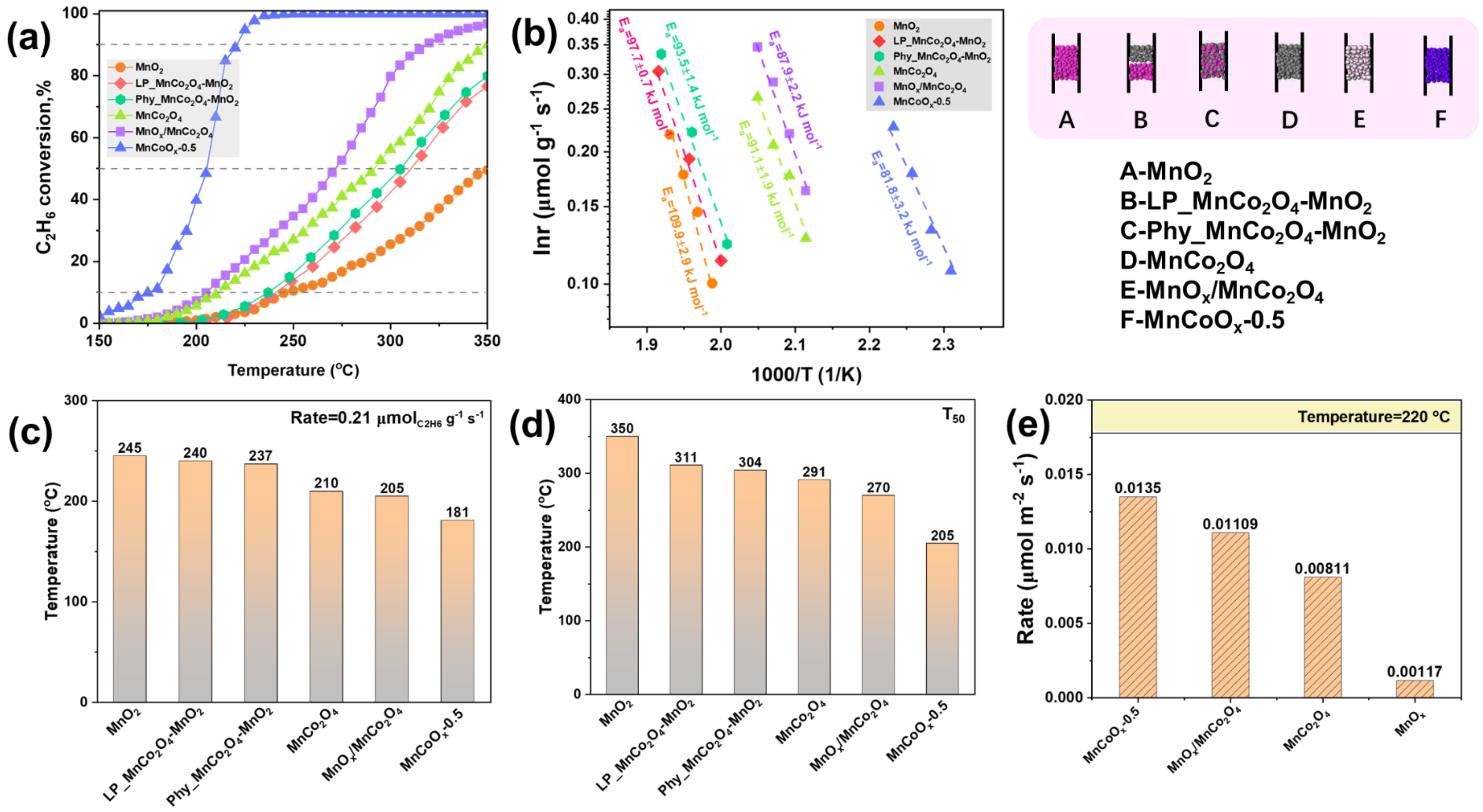

Supplementary Figure 25. Catalytic performance of $MnCoO_x$-0.5 and references in ethane oxidation. (a) Light-off curves; (b) Corresponding Arrhenius plots; (c) Temperature comparation at constant ethane conversion rate; (d) Temperature of 50% ethane conversion (T_{50}) and (e) The areal conversion rate at 220 °C.

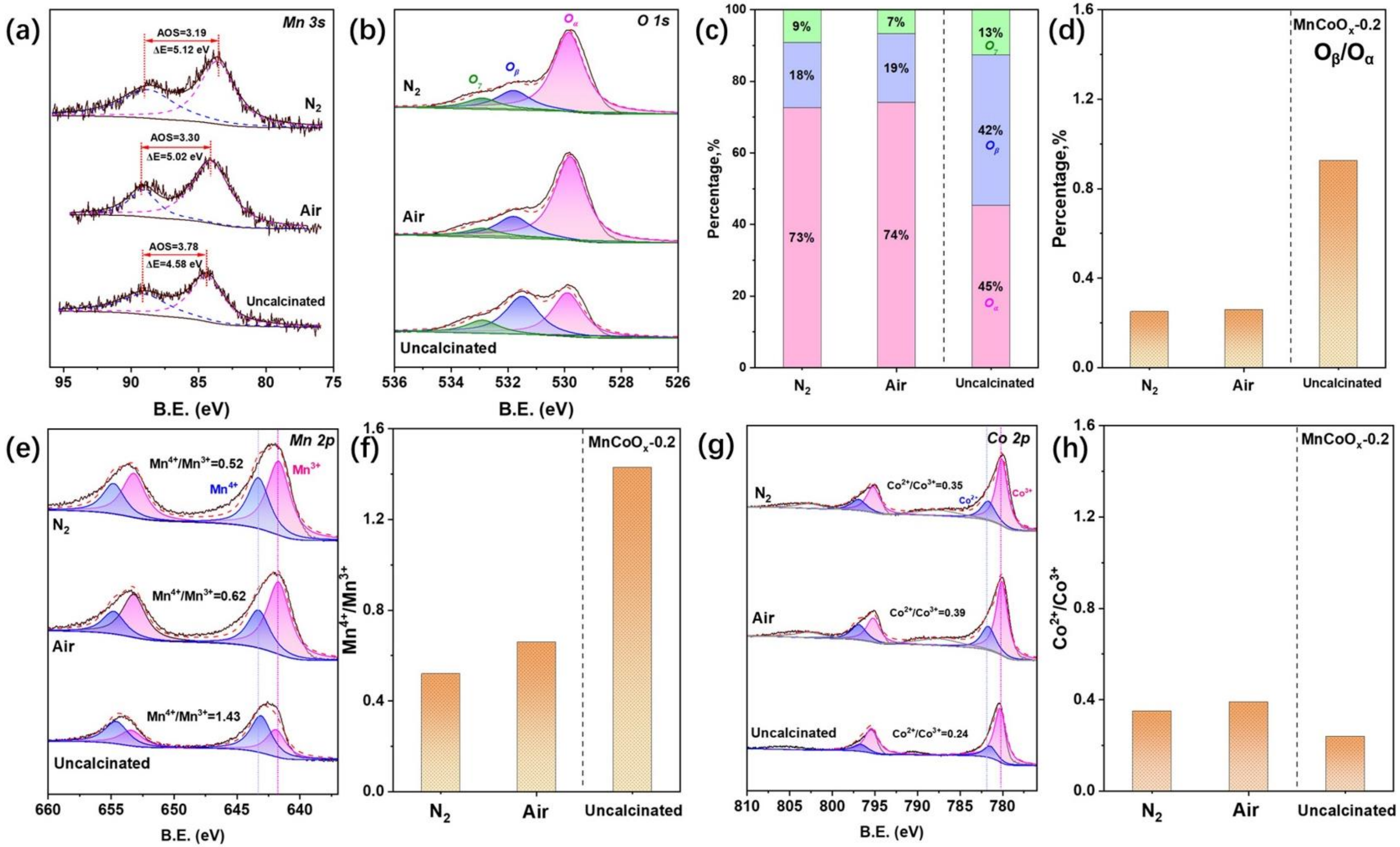

Supplementary Figure 26. XPS spectra of $MnCoO_x$-0.2 annealed at N_2 and air. (a) Mn 3s; (b) O 1s; (c) Percentage of O species; (d) Percentage of O_β/O_α; (e) Mn 2p; (f) Mn^{4+}/Mn^{3+}; (g) Co 2p; (h) Co^{2+}/Co^{3+}.

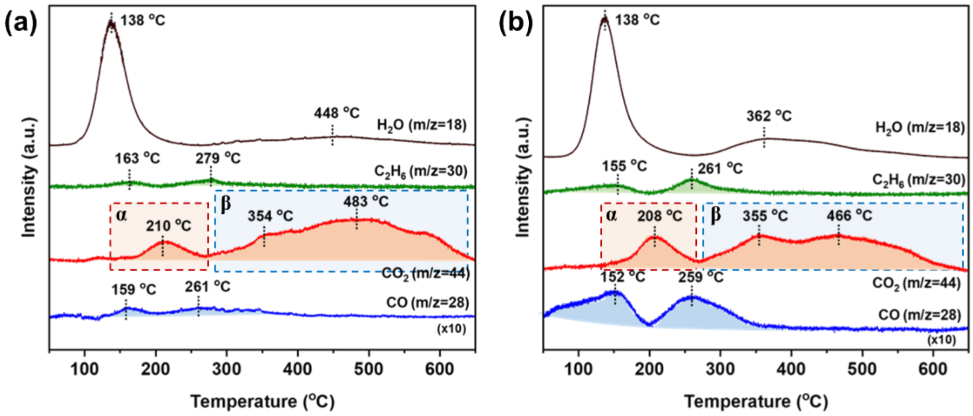

Supplementary Figure 27. C_2H_6-TPD. (a) $MnCoO_x$-0.2-N_2; (b) $MnCoO_x$-0.2-Air.

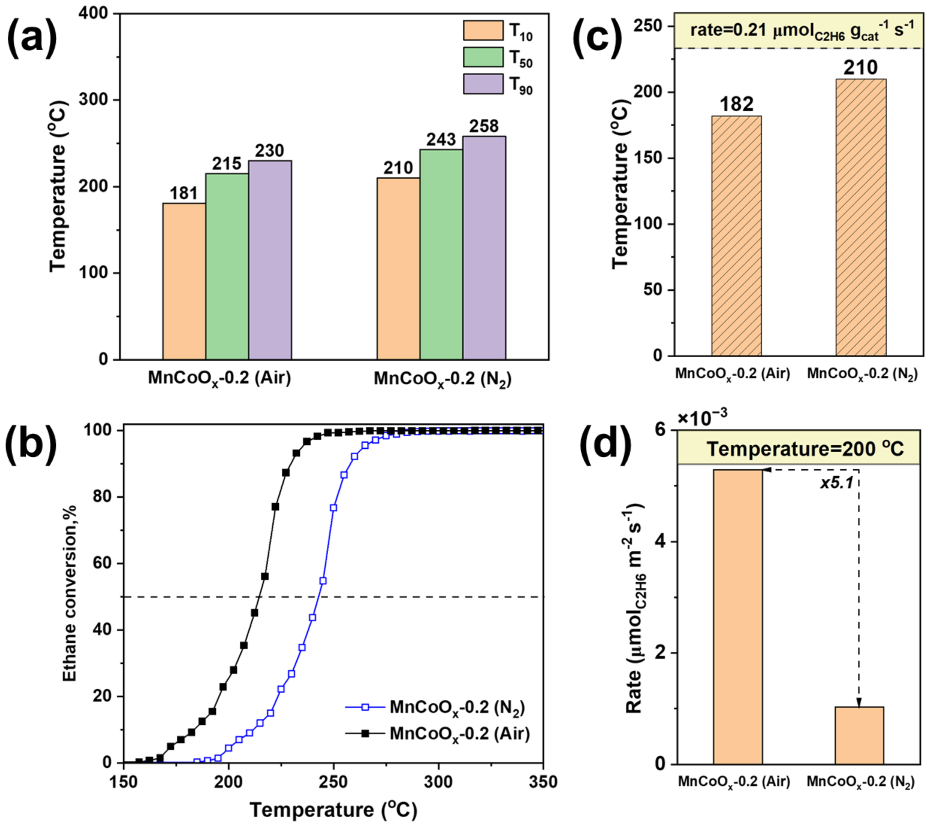

Supplementary Figure 28. Catalytic performance of ethane oxidation over $MnCoO_x$-0.2-N_2 and $MnCoO_x$-0.2-Air. (a) temperatures of ethane conversion at 10%, 50%, and 90%; (b) light-off curve of ethane combustion; (c) comparation of temperature at constant rate of 0.21 μmol g_{cat}^{-1} s^{-1}; (d) areal conversion rate of ethane at 200 ^{o}C.

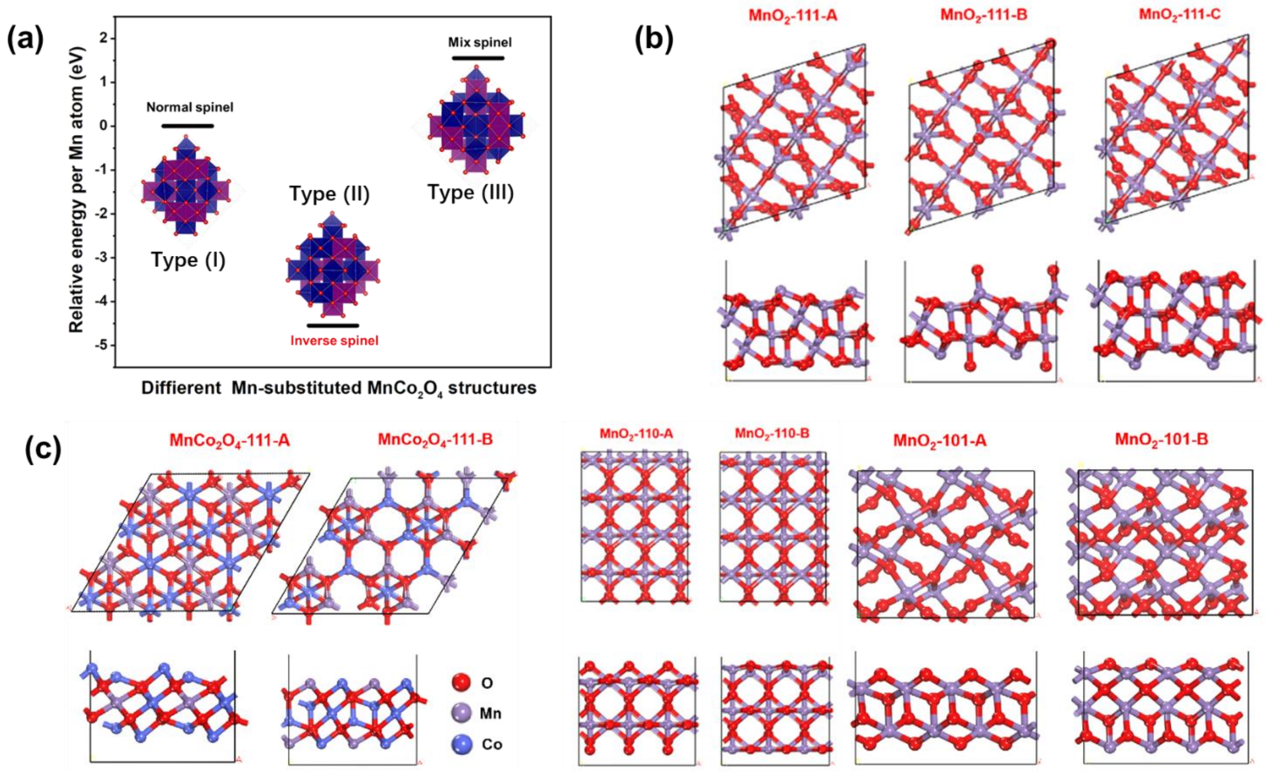

Supplementary Figure 29. (a) Relative energy per Mn atom in the proposed $MnCo_2O_4$ models; (b, c) Structural illustration of top and side views of $MnCo_2O_4$ (111), MnO_2 (111), MnO_2 (110), and β-MnO_2 (101) facets examined in this work.

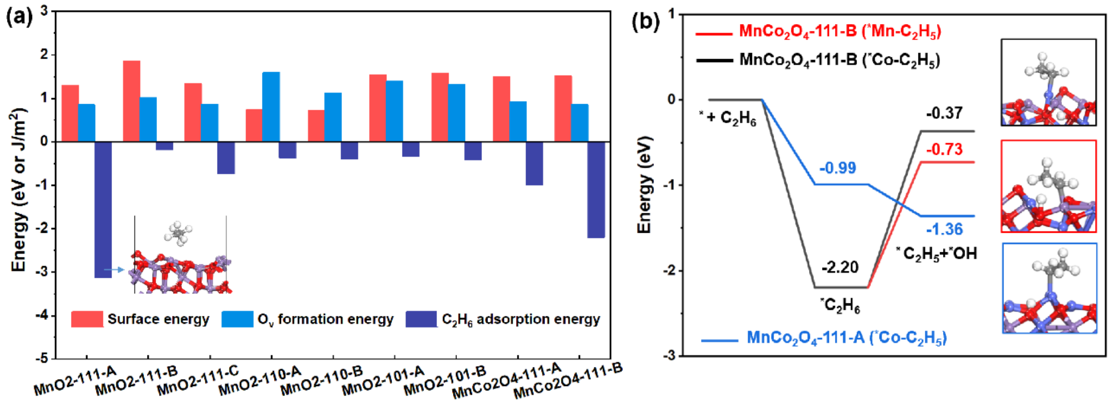

Supplementary Figure 30. (a) The calculated surface energy, O vacancy formation energy (E_{Ov}) and C_2H_6 adsorption energy ($E_{ads,C2H6}$) of $MnCo_2O_4$ and MnO_2 with different termination planes. (b) The dissociation energy of the first C-H bond in C_2H_6 on $MnCo_2O_4$-111-A and $MnCo_2O_4$-111-B planes.

Supplementary Discussion: DFT calculations were performed to evaluate the surface energy of different crystal facets that possess different endpoints to study the termination stability of MnO_2 and $MnCo_2O_4$ catalysts. It was found that the surface energies of different terminations of each crystalline surface do not differ much, as shown in Supplementary Fig. 30a. The oxygen vacancy formation energy (E_{Ov}) over these catalyst models and the adsorption energy of C_2H_6 ($E_{ads,C2H6}$) on different crystalline surfaces of MnO_2 and $MnCo_2O_4$ were calculated. The E_{Ov} of MnO_2-111-A and MnO_2-111-C surfaces are energetically more favorable than that of other facets with different terminations. However, the C_2H_6 adsorption over MnO_2-111-A is too strong and leads to the deformation of catalyst surface, which is detrimental to the C_2H_6 conversion. By contrast, the MnO_2-111-C surface has good structural stability and moderate adsorption strength for C_2H_6. For the (110) and (101) facet of MnO_2, structures of MnO_2-110-B and MnO_2-101-B are more favorable in terms of both oxygen vacancy formation energy and C_2H_6 adsorption energy calculations. While, for $MnCo_2O_4$-111-A and $MnCo_2O_4$-111-B catalyst models, they presented similar surface energies and oxygen vacancy formation energies. The adsorption strength of C_2H_6 is higher on $MnCo_2O_4$-111-B than $MnCo_2O_4$-111-A.

To further determine the active surface of $MnCo_2O_4$, the first C-H bond dissociation energy of C_2H_6 on $MnCo_2O_4$-111-A and $MnCo_2O_4$-111-B planes was calculated, as shown in Supplementary Fig. 30b. The dissociation of C-H bond in C_2H_6 on $MnCo_2O_4$-111-B plane shows a high reaction energy of 1.47 or 1.83 eV, dependent on the adsorption positions along with an obvious deformation of the catalyst surface. While, the dissociation of C-H bond in C_2H_6 on $MnCo_2O_4$-111-A is thermodynamically much more favorable. Therefore, the MnO_2-111-C, MnO_2-110-B, MnO_2-101-B, and $MnCo_2O_4$-111-A surfaces were used in the following study.

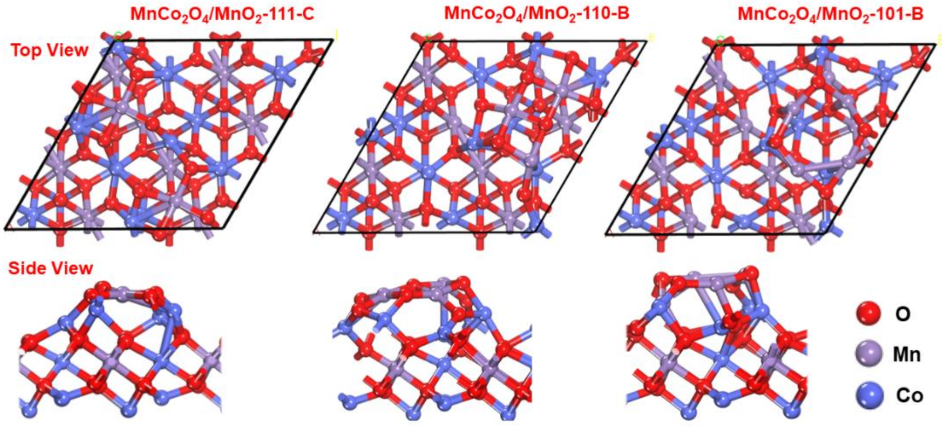

Supplementary Figure 31. Optimized structures of MnO_2-$MnCo_2O_4$ interfacial models (including $MnCo_2O_4$/MnO_2-111-C, $MnCo_2O_4$/MnO_2-110-B, and $MnCo_2O_4$/MnO_2-101-B).

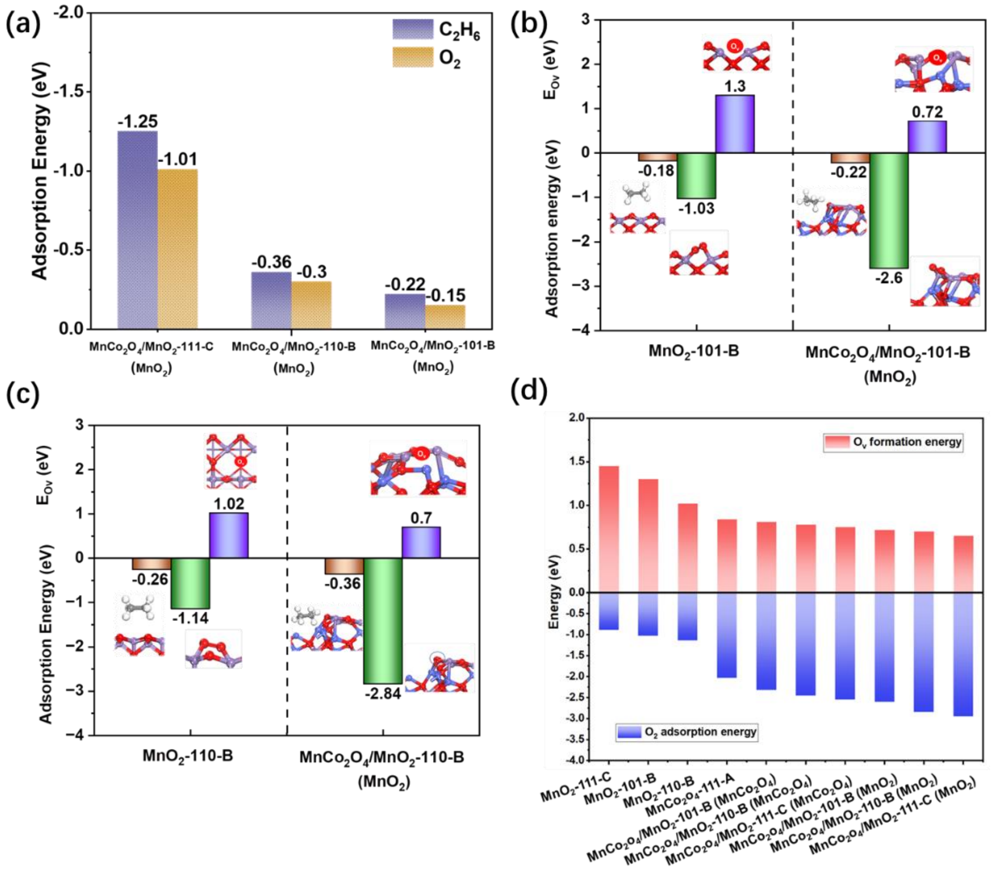

Supplementary Figure 32. (a) Comparation between C_2H_6 and O_2 adsorption energy at the interfacial sites of different $MnCo_2O_4/MnO_2$ models, adsorption energy of C_2H_6 ($E_{C2H6,ads}$), O_2 ($E_{O2,ads}$), and oxygen vacancy formation energy (E_{Ov}) over (b) MnO_2-101-B and $MnCo_2O_4/MnO_2$-101-B and (c) MnO_2-110-B and $MnCo_2O_4/MnO_2$-110-B (Note: The adsorption energy of C_2H_6 was obtained by adsorbing C_2H_6 at the interfacial region of $MnCo_2O_4/MnO_2$-101-B and $MnCo_2O_4/MnO_2$-110-B models; the O_2 adsorption energy was obtained by adsorbing O_2 on the upper MnO_2 cluster of $MnCo_2O_4/MnO_2$-101-B and $MnCo_2O_4/MnO_2$-110-B model), (d) The calculated O vacancy (O_v) formation energy and O_2 adsorption energy of MnO_2, $MnCo_2O_4$, and $MnCo_2O_4/MnO_2$ catalysts with different termination planes.

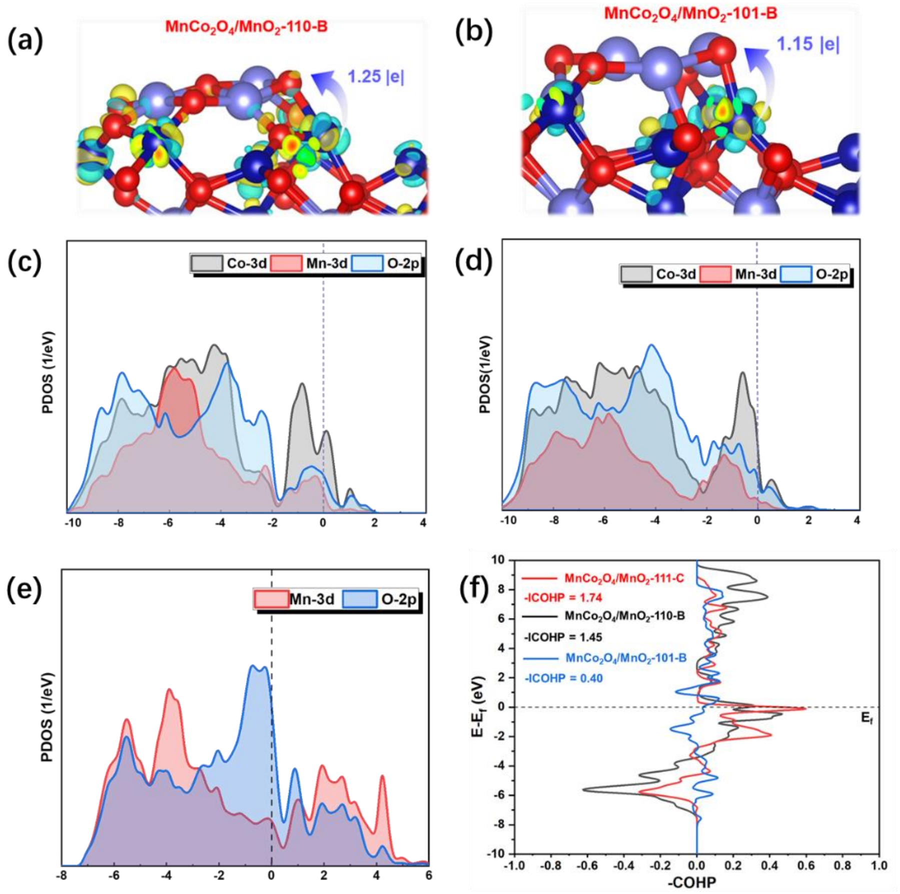

Supplementary Figure 33. Differential charge density analysis between O atom in the MnO_2 cluster and interfacial Co atom over (a) $MnCo_2O_4/MnO_2$-110-B and (b) $MnCo_2O_4/MnO_2$-101-B. The calculated projected density of states (PDOSs) of Co-3d, Mn-3d and O-2p orbital on the (c) $MnCo_2O_4/MnO_2$-110-B, (d) $MnCo_2O_4/MnO_2$-101-B, and (e) MnO_2. The Fermi level was set to zero and the isosurface value was set to be 0.005 e Å^{-3}; cyan and yellow regions represent positive and negative charges, respectively. (f) The crystal orbital Hamilton population (COHP) analysis of Co-O bonds on the $MnCo_2O_4/MnO_2$-111-C, $MnCo_2O_4/MnO_2$-110-B, and $MnCo_2O_4/MnO_2$-101-B catalyst models.

Supplementary Discussion: The differential charge analysis shows a trend of $MnCo_2O_4/MnO_2$-111-C (1.37 |e|)> $MnCo_2O_4/MnO_2$-110-B (1.25 |e|) > $MnCo_2O_4/MnO_2$-101-B (1.15 |e|), which is consistent with the order of dissociation activity of C_2H_6 at the interface (Supplementary Fig. 33(a, b), Fig. 6a). In addition, the partial density of states (PDOSs) shows a significant hybridization between O-2P and Co-3d/Mn-3d orbitals over $MnCo_2O_4/MnO_2$ catalysts (Supplementary Fig. 33(c, d), Fig. 6a) compared to the pure MnO_2 (Supplementary Fig. 33e).

A detailed comparation was conducted among these three $MnCo_2O_4/MnO_2$ catalyst models by COHP calculation. The results in Supplementary Fig. 33f show that the absolute integral value below the Fermi level over $MnCo_2O_4/MnO_2$-111-C catalyst is the highest, which proved the strong electron transfer from $MnCo_2O_4$ substrate to upper MnO_2 domains on the (111) plane, therefore the created interface facilitated the reactant activation. Overall, the above calculation results demonstrate that $MnCo_2O_4/MnO_2$-111-C is the optimal catalyst model for ethane activation, which will be used for further discussion in the present work.

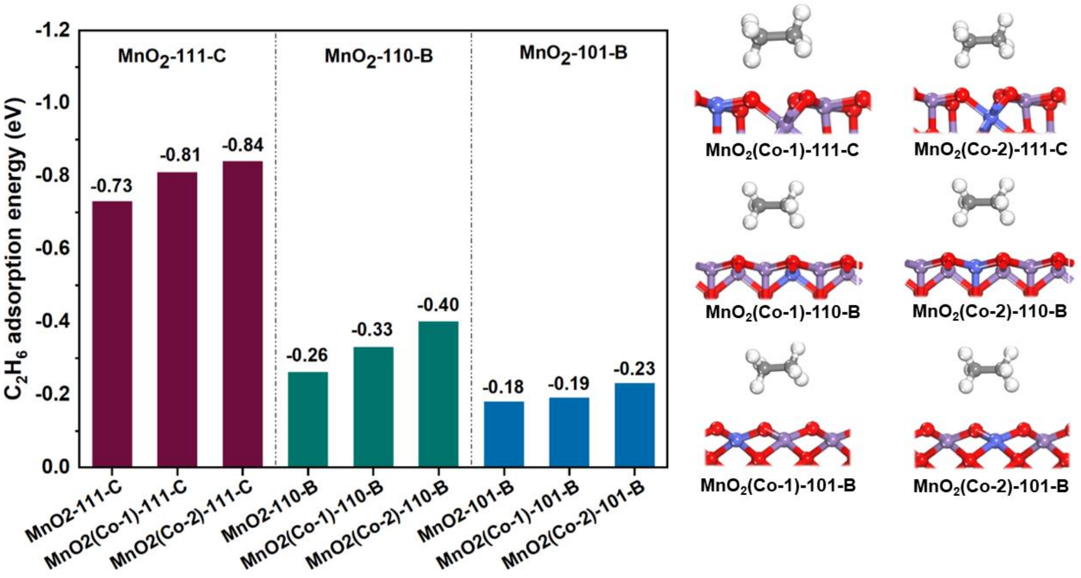

Supplementary Figure 34. The calculated adsorption energies of ethane on Co-doped MnO_2 surfaces and their optimal configurations.

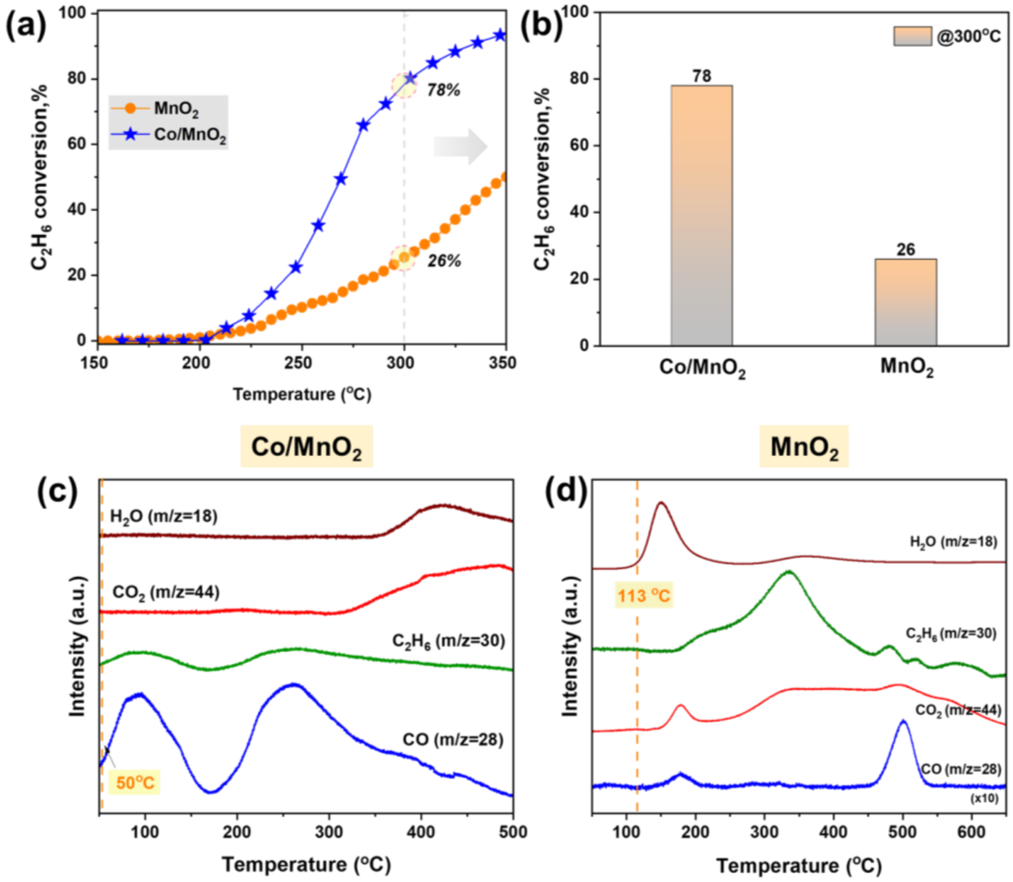

Supplementary Figure 35. (a) Light-off curves; (b) catalytic performance of Co/MnO_2 and MnO_2 in ethane oxidation; (c) C_2H_6-TPD over Co/MnO_2 catalyst; (d) C_2H_6-TPD over MnO_2.

Supplementary Discussion: the ethane conversion of Co/MnO_2 catalyst is higher than that of MnO_2 at 300 °C (Co/MnO_2: conv.=78%; MnO_2: conv.%=26%), indicating the positive role of Co on activating ethane. Then, C_2H_6-TPD was carried out to study the chemisorption behaviour of C_2H_6 over Co/MnO_2 catalyst. It was found that most of the adsorbed C_2H_6 was readily converted into CO at the onset of the TPD process of 50 °C over Co/MnO_2 catalyst, which may result from the strong C-C bond breakage ability over Co-related sites. While, the rest of the adsorbed C_2H_6 was converted into CO_2 and H_2O above 300 °C. Differently, for the pure MnO_2, most of the C_2H_6 was mainly desorbed as C_2H_6 and CO_2 with increased temperature. Also, through calculating the integrated area of C-related species, we found that the amount of adsorbed C_2H_6 was higher on the Co/MnO_2 catalyst compared to the pure MnO_2, perhaps due to the formation of Co-O-Mn bonds at the interface between Co_3O_4 and MnO_2, thus facilities the adsorption of C_2H_6 as well as increases the O mobility at interfacial areas.

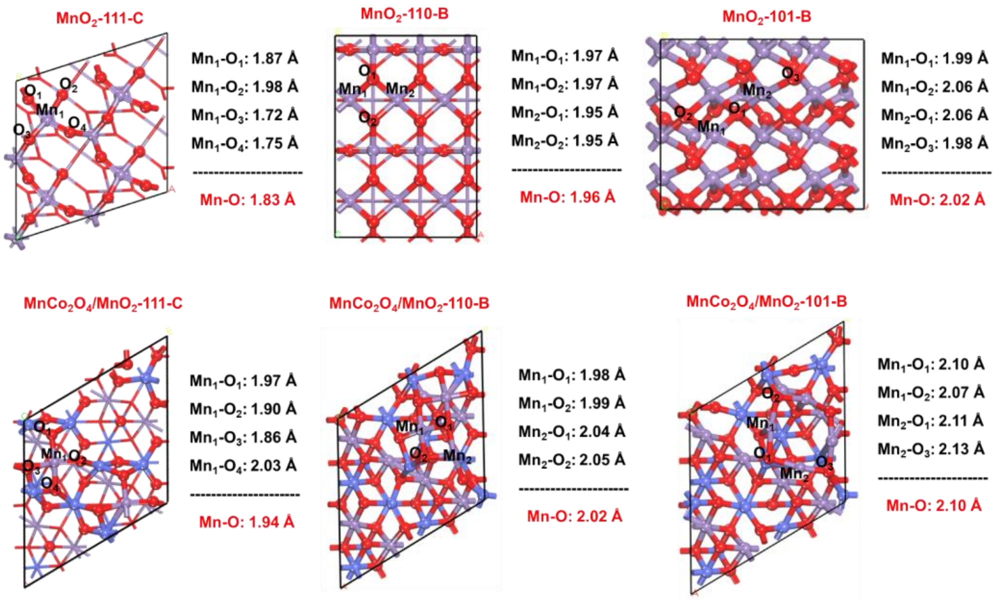

Supplementary Figure 36. Mn-O bond length of MnO_2 and $MnCo_2O_4/MnO_2$ catalyst models.

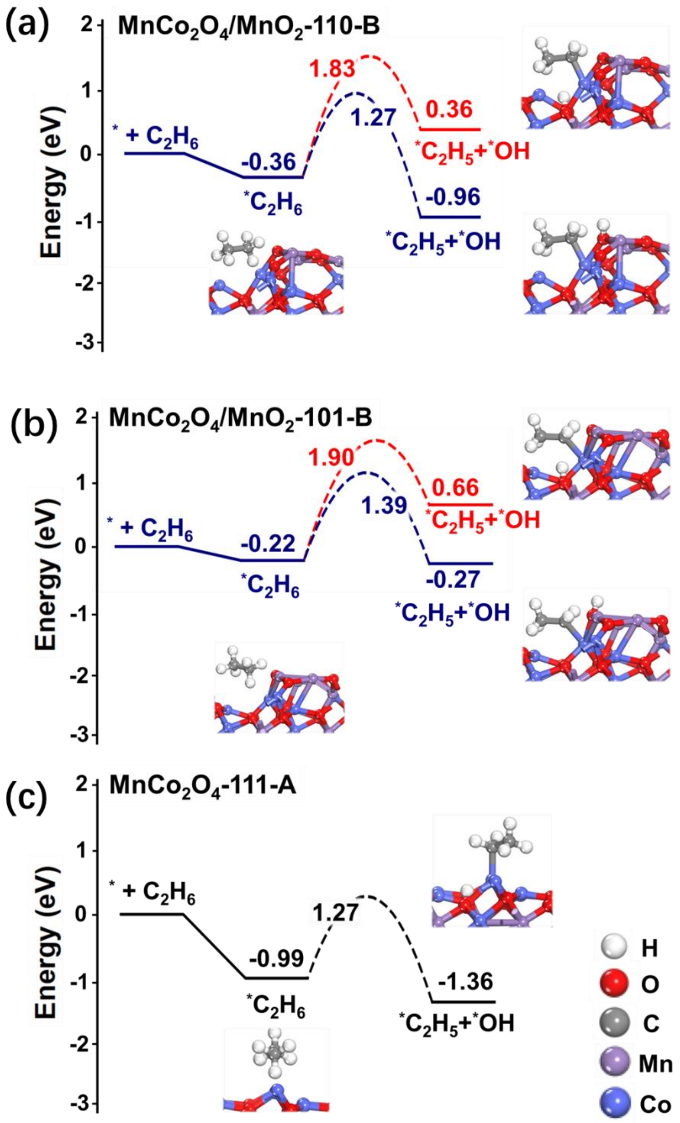

Supplementary Figure 37. Energy profiles for the dissociation of the first C-H bond of C_2H_6 on different sites over (a) $MnCo_2O_4/MnO_2$-110-B, (b) $MnCo_2O_4/MnO_2$-101-B, and (c) $MnCo_2O_4$-111-A (red line: H abstraction of adsorbed C_2H_6 at the interfacial region of $MnCo_2O_4/MnO_2$, blue line: H abstraction of adsorbed C_2H_6 on the upper MnO_2 cluster of $MnCo_2O_4/MnO_2$, black line: H abstraction of adsorbed C_2H_6 on the surface of $MnCo_2O_4$-111-A).

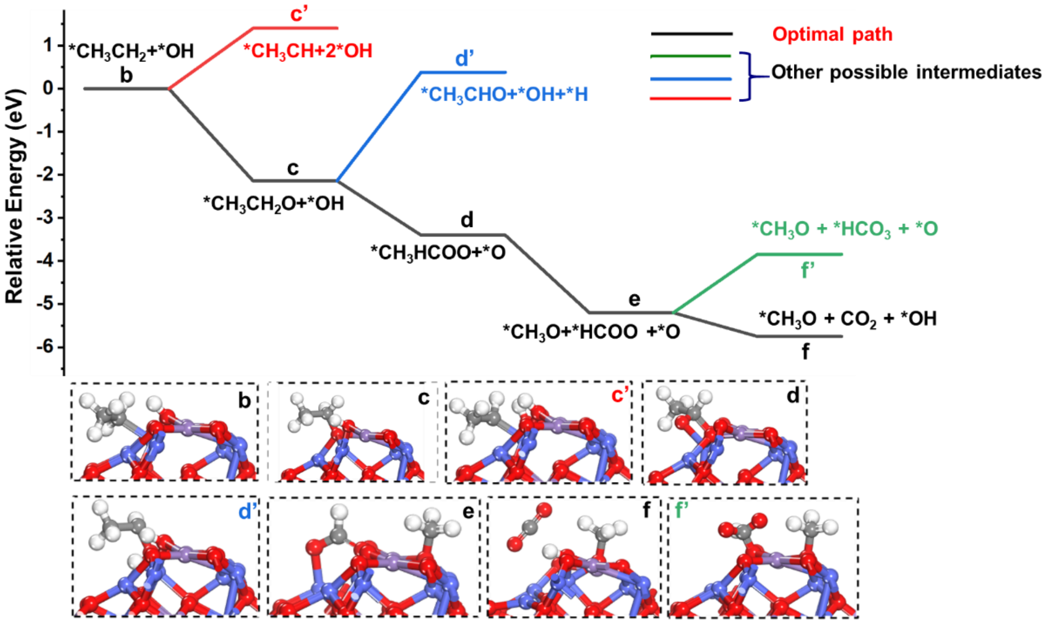

Supplementary Figure 38. Energy diagram of reaction paths for ethane oxidation on the the $MnCo_2O_4/MnO_2$-111-C model catalyst.

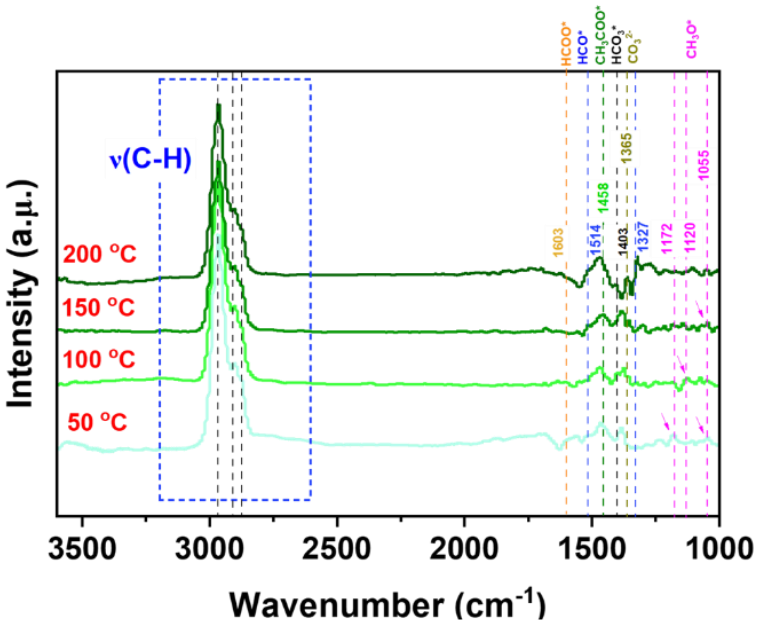

Supplementary Figure 39. *In-situ* DRIFT spectra of C_2H_6 oxidation over $MnCoO_x$-0.5 catalyst.

Supplementary Discussion: DRIFT analysis was conducted to track the reaction intermediates. The DRIFT spectra were recorded after 10 min injection of the feed gas in the temperature range of 50-200 °C (**Supplementary Fig. 39**). Apparently, the peak intensity of C-H stretching vibrations (ca. 3000 cm^{-1}) of ethane decreased with increased temperature, indicating the activation of ethane at ≥100 °C over $MnCoO_x$-0.5. Various oxygenates were generated as indicated by the formation of multiple adsorption bands at 1000-1700 cm^{-1}. Note that, a sharp peak appeared at 1458 cm^{-1}, which can be assigned to acetate (CH_3COO^*) from H abstraction of ethane on lattice O^*. The peak intensity of CH_3COO^* decreased with increased temperature from 50 to 150 °C, indicating their further conversion to other oxygenates. While the amount of CH_3COO^* slightly increased above 150 °C, perhaps due to the participation of MnO_2-$Mn_xCo_{3-x}O_4$ interface, thus producing more active sites for C_2H_6 activation. Moreover, the peaks at 1065, 1120, and 1172 cm^{-1} can be assigned to CH_3O^*, which is likely due to the breakage of C-C bond of CH_3COO^*. The intensity of CH_3O^* gradually decreased with increased temperature because of their further conversion. Aside from these, other dehydrogenation products were detected as well, such as carboxylate acid (HCO^*: 1327 & 1514 cm^{-1}), formate ($HCOO^*$: 1603 cm^{-1}), bicarbonate (HCO_3^*: 1403 cm^{-1}), and carbonate (CO_3^{2-}: 1303 & 1365 cm^{-1}). Overall, this result suggests that there are more O-intermediates involved in ethane oxidation via Mars van Krevelen (MvK) mechanism. (Reaction route: C_2H_6 → C_2H_6-O^* → $CH_3CH_2O^*$ → CH_3HCOO^* → CH_3O^* & $HCOO^*$ → $HCOO^*$, HCO^*, HCO_3^*, CO_3^{2-} → CO_2, H_2O).

Supplementary Tables

Supplementary Table 1. Lattice parameter calculation from XRD measurements.

Sample	Mn:Co	$d_{(311)}$	Lattice parameter a (a=b=c)
Co_3O_4	0	0.2436	0.8079
$MnCoO_x$-0.1	0.1	0.2451	0.8128
$MnCoO_x$-0.2	0.2	0.2464	0.8173
$MnCoO_x$-0.5	0.5	0.2469	0.8190
$MnCoO_x$-1.0	1.0	0.2474	0.8205
$MnCoO_x$-2.0	2.0	0.2474	0.8205

Supplementary Table 2. Summary of the determination and quantitative analysis for surface Mn, Co, and O.

Sample	XPS analysis				Surface Mn/Co ratio [a]	Bulk Mn/Co Ratio [b]	H_2-TPR			
	Co^{2+}/Co^{3+}	Mn^{4+}/Mn	Mn^{3+}/Mn^{4+}	O_β/O_α			Peak I (160 ºC)	Peak II (220-250 ºC)	Peak III (350-380 ºC)	Peak IV (440-460 ºC)
$MnCoO_x$-0.1	0.38	0.36	1.77	0.23	0.50	0.40	20.5	17.1	62.4	0.0
$MnCoO_x$-0.2	0.39	0.38	1.61	0.26	0.57	0.41	10.9	26.3	62.8	0.0
$MnCoO_x$-0.5	0.55	0.43	1.34	0.34	0.62	0.42	8.3	26.0	65.7	0.0
$MnCoO_x$-1.0	0.56	0.44	1.26	0.17	0.78	0.44	11.4	24.7	36.5	27.3
$MnCoO_x$-2.0	0.57	0.53	0.89	0.17	1.19	0.47	n.a.	n.a.	n.a.	n.a.

[a] Surface atomic ratio of Mn to Co in bulk $MnCoO_x$ samples measured by XPS. [b] Bulk atomic ratio of Mn to Co in bulk MnCoOx samples measured by ICP-OES.…

Supplementary Note: Peak (I) represents surface adsorbed oxygen; peak (II) represents surface MnO_2; peak (III) represents Co oxides; peak (IV) represents the bulk phase of MnCoOx oxides.

Supplementary Table 3. XPS analysis of $MnCoO_x$, Co_3O_4, and MnO_2 references.

Sample	Co*2p*$_{3/2}$ composition, %					Mn*2p*$_{3/2}$ composition, %			O*1s* composition, %		
	Co^{3+} 780.1 eV	Co^{2+} 781.7 eV	Sat.(II) 786.0 eV	Sat.(I) 802.9 eV	Co^{2+}/Co^{3+}	Mn^{3+} 641.7 eV	Mn^{4+} 643.2 eV	Mn^{3+}/Mn^{4+}	Oα 529.7 eV	Oβ 531.6 eV	Oγ 532.8 eV
Co_3O_4	67.7	23.9	4.6	3.7	0.35	n/a	n/a	n/a	n/a	n/a	n/a
$MnCoO_x$-0.1	63.5	24.3	7.8	4.4	0.38	63.9	36.1	1.77	75.3	13.5	11.2
$MnCoO_x$-0.5	53.8	30.2	10.7	5.3	0.55	57.2	42.8	1.34	85.4	14.6	0
$MnCoO_x$-2.0	54	31.2	10.5	4.3	0.57	47.1	52.9	0.89	82.7	13.4	3.9
MnO_2	n/a	n/a	n/a	n/a	n/a	26.3	73.7	0.36	77.9	16.5	5.6

Supplementary Table 4. O_2-TPD of $MnCoO_x$ catalysts and references.

Sample	Percentage of adsorbed O_2			Total O_2 desorption amount (μmol g^{-1})[a]	O_2-TPD of peak II (μmol g^{-1})[b]	Amount of surface lattice O (μmol g^{-1})[c]
	Peak (I)	Peak (II)	Peak (III)			
$MnCoO_x$-0.1	9.03	55.00	35.98	226.8	124.7	249.5
$MnCoO_x$-0.2	5.46	52.92	41.62	304.2	160.9	321.9
$MnCoO_x$-0.5	3.61	55.81	40.59	345.1	192.6	385.2
$MnCoO_x$-1.0	4.72	38.49	56.79	310.9	119.7	239.4
$MnCoO_x$-2.0	9.75	35.86	54.39	278.2	99.8	199.5
Co_3O_4	12.27	37.50	50.24	220.2	82.6	165.1
MnO_2	8.19	50.24	41.57	263.2	132.2	264.5

[a] Total O_2-desorption amount from O_2-TPD.

[b] O_2-TPD of peak II (μmol g^{-1}) = Total O_2-desorption amount from O_2-TPD (μmol g^{-1}) x Percentage of Peak (II) %.

[c] Amount of surface lattice O = Amount of desorbed O_2 from TPD (refers to Peak II) x 2.

Supplementary Table 5. Catalytic performance of prepared $MnCoO_x$ catalysts for ethane (C_2H_6) oxidation at GHSV of 60,000 h^{-1}.

Sample	Ethane combustion			CO titration number	TOF	TOF	E_a
	T_{10} (ºC)[a]	T_{50} (ºC)[a]	T_{90} (ºC)[a]	(μmol g^{-1})	(h^{-1}) [b]	(h^{-1}) [c]	(kJ mol^{-1}) [d]
$MnCoO_x$-0.1	183	219	235	19.0	8.55E+01	1.81E-03	103.4±1.1
$MnCoO_x$-0.2	181	215	230	19.3	1.06E+02	1.94E-03	98.3±0.7
$MnCoO_x$-0.5	175	205	220	22.6	1.42E+02	2.30E-03	81.8±3.2
$MnCoO_x$-1.0	195	260	345	11.3	7.83E+01	1.67E-03	108.7±2.2
$MnCoO_x$-2.0	202	282	350	9.5	8.29E+01	1.02E-03	115.6±1.7
Co_3O_4	216	325	n/a	8.6	4.20E+01	6.08E-04	110.2±2.5
MnO_2	250	348	n/a	2.3	3.49E+01	8.44E-05	109.9±2.9

[a] Temperature at 10, 50, and 90% conversion of ethane.

[b] Turnover frequency based on CO titration measured active sites at 200 ºC.

[c] Turnover frequency (TOF) of C_2H_6 conversion at 200 ºC by normalizing the amounts of surface lattice oxygen (O*).

[d] Apparent activation energy obtained from Arrhenius plots at ethane conversion in the range of 5-10%.

Supplementary Table 6. Catalytic performance of prepared $MnCoO_x$ catalysts for propane (C_3H_8) oxidation at GHSV of 60,000 h^{-1}.

Sample	Propane combustion			CO titration number	Rate	TOF	TOF	E_a
	T_{10} (ºC)[a]	T_{50} (ºC)[a]	T_{90} (ºC)[a]	(μmol g^{-1})	(mmol mL^{-1} h^{-1})[b]	(h^{-1})[c]	(h^{-1}) [d]	(kJ mol^{-1})[e]
$MnCoO_x$-0.1	152	168	188	19.0	1.10	2.90E+01	6.14E-04	90±2
$MnCoO_x$-0.2	141	166	181	19.3	2.80	7.25E+01	1.21E-03	89±3
$MnCoO_x$-0.5	141	164	177	22.6	3.07	6.80E+01	1.11E-03	88±1
$MnCoO_x$-1.0	175	209	275	11.3	0.09	4.05E+00	5.33E-05	91±1
$MnCoO_x$-2.0	185	207	230	9.5	0.05	2.43E+00	3.20E-05	92±2
Co_3O_4	190	255	295	8.6	0.43	2.48E+01	3.59E-04	124±2
MnO_2	207	263	n.a.	2.3	0.00	0.00E+00	0.00E+00	99±1

[a] Temperature at 10, 50, and 90% conversion of propane.

[b] Reaction rate of propane converted over various catalysts at 150 ºC

[c] Turnover frequency based on CO titration measured active sites at 150 ºC.

[d] Turnover frequency (TOF) of C_3H_8 conversion at 150 ºC by normalizing the amounts of surface lattice oxygen (O*).

[e] Apparent activation energy obtained from Arrhenius plots.

Supplementary Table 7. Catalytic performance of prepared $MnCoO_x$ catalysts for methane (CH_4) oxidation at GHSV of 60,000 h^{-1}.

Sample	Methane combustion T_{10} (°C) [a]	CO titration number (μmol g^{-1})	Rate (mmol mL^{-1} h^{-1}) [b]	TOF (h^{-1}) [c]	TOF (h^{-1}) [d]	E_a (kJ mol^{-1}) [e]
$MnCoO_x$-0.1	275	19.0	0.20	5.29E+00	1.12E-04	139±1
$MnCoO_x$-0.2	255	19.3	0.99	2.57E+01	4.28E-04	139±2
$MnCoO_x$-0.5	245	22.6	1.98	4.41E+01	7.17E-04	137±1
$MnCoO_x$-1.0	275	11.3	0.72	3.19E+01	4.19E-04	140±1
$MnCoO_x$-2.0	295	9.5	0.20	1.06E+01	1.39E-04	141±1
Co_3O_4	325	8.6	0.04	2.34E+00	3.38E-05	173±1
MnO_2	n.a.	2.3	0.00	00E+00	0.00E+00	152±1

[a] Temperature at 10, 50, and 90% conversion of methane.

[b] Reaction rate of methane converted over various catalysts at 250 °C

[c] Turnover frequency based on CO titration measured active sites at 250 °C.

[d] Turnover frequency (TOF) of CH_4 conversion at 250 °C by normalizing the amounts of surface lattice oxygen (O*).

[e] Apparent activation energy obtained from Arrhenius plots.

Supplementary Table 8. Comparation of catalytic performance of Co-based catalysts for low-chain alkane combustion with literature compositions.

Catalyst	Alkane	Experimental conditions	T_{10}	T_{50}	T_{90}	Ref.
$MnCoO_x$-0.5	CH_4<br>C_2H_6<br>C_3H_8	3000 ppm<br>WHSV=60,000 h^{-1}, Q=200 mL min^{-1}(0.2 g cat.)	245<br>175<br>141	280<br>205<br>164	335<br>220<br>177	This work
Co_3O_4-8h Aging	CH_4	0.5 vol%CH_4, 8 vol%O_2, 91.5 vol% He,<br>Q=40 mL min^{-1}(0.2 g cat.)	225	295	355	4
PdO/CeO_2-0.1/Co_3O_4	CH_4	0.5 vol%CH_4, 2 vol%O_2, 97.5 vol% He,<br>Q=30 mL min^{-1} (0.2 g cat.)	245	300	350	5
Co-In-0.2	CH_4	1.0 vol%CH_4, 10 vol%O_2 balanced by N_2,<br>Q=80 mL min^{-1}(0.1 g cat.)	265	320	380	6
Co_3O_4-AC<br>Co_3O_4-Cl<br>Co_3O_4-N<br>Co_3O_4-S	CH_4	0.5 vol%CH_4, 2 vol%O_2, 97.5 vol% He,<br>Q=30 mL min^{-1}(0.2 g cat.)	260<br>265<br>270<br>305	318<br>332<br>344<br>367	385<br>400<br>416<br>429	7
Co_3O_4-9.0	CH_4	0.5 vol%CH_4, 8 vol%O_2 balanced by N_2<br>Q=60 mL min^{-1}(0.2 g cat.)	265	344	413	8
Pd/Co-OMA-6	CH_4	1.0 vol%CH_4, 10 vol%O_2 balanced by N_2<br>Q=100 mL min^{-1}(0.2 g cat.)	340	400	450	9
ZrO_2(2)-Co_3O_4	CH_4	0.5 vol%CH_4, 8.0 vol%O_2 balanced by N_2<br>Q=40 mL min^{-1} (0.2 g cat.)	220	280	335	10
5%Pd/Co_3O_4	CH_4	0.5 vol%CH_4, 8.0 vol%O_2 balanced by N_2<br>Q=100 mL min^{-1} (0.1 g cat.)	300	350	410	11
m-$CuCo_2O_4$	CH_4	1500 ppm CH_4, 12 vol%O_2 balanced by N_2<br>Q=200 mL min^{-1} (0.2 g cat.)	280	369	440	12
30Co/12Mg-Al_2O_3	CH_4	1.0 vol%CH_4, 10.0 vol%O_2 balanced by N_2<br>Q=300 mL min^{-1} (0.3 g cat.)	415	525	585	13
Pd-$NiCo_2O_4/SiO_2$	CH_4	1.0 vol%CH_4 balanced by Air<br>Q=40 mL min^{-1} (0.08 g cat.)	290	330	360	14
$NiCo_2O_4$	CH_4	1000 ppm CH_4, 10%O_2, balanced by Ar and He<br>Q=150 mL min^{-1} (0.1 g cat.)	270	340	385	15
$La_{0.7}Bi_{0.3}Mn_{0.5}Co_{0.5}O_3$	C_2H_6	2000 ppm C_2H_6, 6 vol% CO balanced by N_2<br>Q=40 mL min^{-1} (0.2 g cat.)	658	692	718	16

4%Mn/SnO_2<br>4%Co/SnO_2	C_2H_6	2000 ppm C_2H_6 in air, GHSV = 20,000 h^{-1}	250<br>280	310<br>340	360<br>380	17
Au/CoO_x	C_2H_6	5000 ppm C_2H_6 in air, GHSV = 15,000 h^{-1}	160	190	230	18
$SmCoO_3$<br>$PrCoO_3$	C_2H_6	W/F=38.3 g h mol^{-1}	375[a]	N/A	N/A	19
$Co_2Ce_1O_x$	C_3H_8	800 ppm C_3H_8, 10 vol.% O_2 balanced by N_2,<br>Q=300 mL min^{-1}	185	220	249	20
Co_3O_4-E<br>Co_3O_4-F<br>Co_3O_4-S<br>Co_3O_4-B	C_3H_8	0.2 vol%C_3H_8, 5 vol%O_2 balanced by Ar,<br>GHSV=120,000 mL g^{-1} h^{-1}	230<br>223<br>222<br>220	269<br>258<br>272<br>250	295<br>285<br>297<br>278	21
Pt/$LaCoO_3$-He-O_2-H_2	C_3H_8	8000 ppm C_3H_8 balanced by air,<br>Q=100 mL min^{-1}, WHSV=42,857 h^{-1}	260	330	445	22
$Ni_{0.27}Co_{2.73}O_4$	C_3H_8	1 vol%C_3H_8, 10%O_2, 9%N_2 and 80%Ar,<br>Q=150 mL min^{-1}, GHSV=45,000 h^{-1}	250	320	395	23
$MnNi_{0.09}$	C_3H_8	1000 ppm C_3H_8, 5 vol%O_2 balanced by N_2,<br>Q=300 mL min^{-1}, WHSV=90 L g^{-1} h^{-1}	185	200	220	24
Co_1Cr_2	C_3H_8	0.2 vol%C_3H_8, 2 vol%O_2 balanced by N_2,<br>Q=67 ml min^{-1}, GHSV=80,000 mL g^{-1} h^{-1}	220	238	275	25
Co_3O_4<br>(Carbonate precipitate)	C_3H_8	0.1 vol%C_3H_8, 21 vol%O_2 balanced by He<br>Q=400 mL min^{-1}, WHSV=160,000 mL h^{-1} g^{-1}	160	190	220	26
Co_3O_4 (pH=9.5)	C_3H_8	1000 ppm C_3H_8, 21 vol%O_2 balanced by N_2,<br>Q=100 mL min^{-1}	197	215	245	27

Supplementary Table 9. Mn/Co bulk atomic ratio, specific surface area, pore volume, and pore size of $MnCoO_x$ catalysts. .

No.	Sample	Specific surface area, $m^2\ g^{-1}$	Pore volume, $cm^3\ g^{-1}$	Ave. pore size, nm
1	Co_3O_4	45	0.70	31.6
2	$MnCoO_x$-0.1	111	0.15	5.5
3	$MnCoO_x$-0.2	124	0.17	5.4
4	$MnCoO_x$-0.5	140	0.18	5.0
5	$MnCoO_x$-1.0	139	0.19	5.5
6	$MnCoO_x$-2.0	130	0.19	6.0
7	MnO_2	57	0.17	11.6

Supplementary Note: The surface area of Mn_xCoO_y catalysts increased with increased Mn/Co ratio up to 0.5 and slightly decreased after that.

Supplementary Table 10. Specific ethane conversion rate at T_{50} with catalyst reported in the literature..

Sample	T_{50} (^oC)	Specific rate (umol g_{cat}^{-1} s^{-1})	Reference
$MnCoO_x$-0.5	205	0.8876	This work
$La_{0.7}Bi_{0.3}Mn_{1-x}Co_xO_3$ (x=0.25)	427	0.1488	
$La_{0.7}Bi_{0.3}Mn_{1-x}Co_xO_3$ (x=0.50)	692	0.1488	16
$La_{1-x}K_xMnO_{3+\delta}$	375	2.924	
4%Mn/SnO_2	310	0.03569	17
4%Co/SnO_2	340	0.03569	
3%Au/CoO_x	190	1.86	18
3%Au/MnO_x	270	1.86	
$SmCoO_3$	375	0.731	19
$PrCoO_3$			
Co_3O_4	345	0.2976	
$NiCo_2O_4$-TM	306	0.2976	28
$NiCo_2O_4$-OL	327	0.2976	
$NiCo_2O_4$-PC	400	0.2976	
2wt%Pt/CZ11	270	0.31	29
2wt%Pd/γ-Al_2O_3	410	1.16	30
1wt%Cu/ZSM-5	340	0.0017	31
α-Fe_2O_3 (110)	375		
α-Fe_2O_3 (202)	450	0.0355	32
α-Fe_2O_3 (012)	475		

Supplementary Table 11. *In-situ* XPS analysis of $MnCoO_x$-0.5 catalyst.

Sample	Co2$p_{3/2}$ composition, %			Mn2$p_{3/2}$ composition, %			O 1s, %	
	Co^{3+} 780.1 eV	Co^{2+} 781.7 eV	Co^{2+}/Co^{3+}	$Mn^{\delta+}$	Mn^{4+} 643.2 eV	$Mn^{\delta+}/Mn^{4+}$	Oα	Oβ 531.6 eV
C_2H_6-RT	64.5	35.5	0.55	48.5 (641.8 eV)	51.5	0.94	85.4 (530.1 eV)	14.6
C_2H_6-200C	61.1	38.9	0.64	57.8 (641.8 eV)	42.2	1.34	85.4 (529.9 eV)	14.6
C_2H_6-300C	60.4	39.6	0.66	67.6 (641.6 eV)	32.4	2.09	82.1 (529.5 eV)	17.9
C_2H_6-400C	61.2	38.8	0.63	76.1 (641.1 eV)	23.9	3.18	74.8 (529.5 eV)	25.2
C_2H_6-O_2-400C	66.1	33.9	0.51	67.8 (641.4 eV)	32.2	2.11	91.1 (529.4 eV)	8.9

Supplementary Table 12. Synthesis parameters of $MnCoO_x$ catalysts.

Catalysts	Solution I		Solution II			
	$KMnO_4$, g	Solution, mL (H_2O)	$Co(NO_3)_2 \cdot 6H_2O$, g	Potassium Citrate, g	H_2O Solution, mL	Injection speed, mL/min
MnCoOx-0.1	1	1000	18.25	2.5	200	6.7
MnCoOx-0.2	1	1000	9.13	1.25	100	3.3
MnCoOx-0.5	1	1000	3.65	0.50	40	1.33
MnCoOx-1.0	1	1000	1.8250	0.250	20	0.67
MnCoOx-2.0	1	1000	0.9125	0.125	10	0.33

Supplementary References

1. Chen, H.; He, J., Facile synthesis of monodisperse manganese oxide nanostructures and their application in water treatment. *J. Phys. Chem. C* **2008,** *112* (45), 17540-17545.
2. Wang, W.; Kan, Y.; Yu, B.; Pan, Y.; Liew, K.; Song, L.; Hu, Y., Synthesis of MnO_2 nanoparticles with different morphologies and application for improving the fire safety of epoxy. *Compos. Part A: Appl. Sci. Manuf.* **2017,** *95*, 173-182.
3. Zhu, L.; Wang, J.; Rong, S.; Wang, H.; Zhang, P., Cerium modified birnessite-type MnO_2 for gaseous formaldehyde oxidation at low temperature. *Appl. Catal. B: Environ.* **2017,** *211*, 212-221.
4. Pu, Z.; Zhou, H.; Zheng, Y.; Huang, W.; Li, X., Enhanced methane combustion over Co_3O_4 catalysts prepared by a facile precipitation method: Effect of aging time. *Appl. Surf. Sci.* **2017,** *410*, 14-21.
5. Li, W.; Liu, D.; Feng, X.; Zhang, Z.; Jin, X.; Zhang, Y., High-Performance ultrathin Co_3O_4 nanosheet supported PdO/CeO_2 catalysts for methane combustion. *Adv. Energy Mater.* **2019,** *9* (18), 1803583.
6. Zheng, Y.; Wang, C.; Li, J.; Zhong, F.; Xiao, Y.; Jiang, L., Enhanced methane oxidation over Co_3O_4–In_2O_{3-x} composite oxide nanoparticles via controllable substitution of Co^{3+}/Co^{2+} by In^{3+} ions. *ACS Appl. Nano Mater.* **2020,** *3* (9), 9470-9479.
7. Zheng, Y.; Yu, Y.; Zhou, H.; Huang, W.; Pu, Z., Combustion of lean methane over Co_3O_4 catalysts prepared with different cobalt precursors. *RSC Adv.* **2020,** *10* (8), 4490-4498.
8. Zheng, Y.; Liu, Y.; Zhou, H.; Huang, W.; Pu, Z., Complete combustion of methane over Co_3O_4 catalysts: Influence of pH values. *J. Alloys Compd.* **2018,** *734*, 112-120.
9. Lin, J.; Chen, Y.; Liu, X.; Chen, X.; Zheng, Y.; Huang, F.; Xiao, Y.; Zheng, Y.; Jiang, L., Microstructural property regulation and performance in methane combustion reaction of ordered mesoporous alumina supported palladium-cobalt bimetallic catalysts. *Appl. Catal. B: Environ.* **2020,** *263*, 118269.
10. Pu, Z.; Liu, Y.; Zhou, H.; Huang, W.; Zheng, Y.; Li, X., Catalytic combustion of lean methane at low temperature over ZrO_2-modified Co_3O_4 catalysts. *Appl.Surf. Sci.* **2017,** *422*, 85-93.
11. Ercolino, G.; Stelmachowski, P.; Grzybek, G.; Kotarba, A.; Specchia, S., Optimization of Pd catalysts supported on Co_3O_4 for low-temperature lean combustion of residual methane. *Appl. Catal. B: Environ.* **2017,** *206*, 712-725.
12. Lim, T. H.; Park, S. B.; Kim, J. M.; Kim, D. H., Ordered mesoporous MCo_2O_4 (M=Cu, Zn and Ni) spinel catalysts with high catalytic performance for methane combustion. *J. Mol. Catal. A: Chem.* **2017,** *426*, 68-74.
13. Choya, A.; de Rivas, B.; González-Velasco, J. R.; Gutiérrez-Ortiz, J. I.; López-Fonseca, R., On the beneficial effect of MgO promoter on the performance of Co_3O_4/Al_2O_3 catalysts for combustion of dilute methane. *Appl. Catal. A: Gen.* **2019,** *582*, 117099.
14. Huang, Q.; Li, W.; Lin, Q.; Zheng, X.; Pan, H.; Pi, D.; Shao, C.; Hu, C.; Zhang, H., Catalytic performance of Pd–$NiCo_2O_4/SiO_2$ in lean methane combustion at low temperature. *J. Energy Inst.* **2018,** *91* (5), 733-742.
15. Dai, Y.; Kumar, V. P.; Zhu, C.; Wang, H.; Smith, K. J.; Wolf, M. O.; MacLachlan, M. J., Bowtie‐shaped $NiCo_2O_4$ catalysts for low‐temperature methane combustion. *Adv. Funct. Mater.* **2019,** *29* (8), 1807519.
16. Lee, Y. N.; Lago, R. M.; Fierro, J. L. G.; Cortés, V.; Sapiña, F.; Martínez, E., Surface properties and catalytic performance for ethane combustion of $La_{1-x}K_xMnO_{3+\delta}$ perovskites. *Appl. Catal. A: Gen.* **2001,** *207* (1-2), 17-24.
17. Tahir, S. F.; Koh, C. A., Catalytic oxidation of ethane over supported metal oxide catalysts. *Chemosphere* **1997,** *34* (8), 1787-1793
18. Solsona, B. E.; Garcia, T.; Jones, C.; Taylor, S. H.; Carley, A. F.; Hutchings, G. J., Supported gold catalysts for the total oxidation of alkanes and carbon monoxide. *Appl. Catal. A: Gen.* **2006,** *312*, 67-76.
19. Alifanti, M.; Bueno, G.; Parvulescu, V.; Parvulescu, V. I.; Cortés Corberán, V., Oxidation of ethane on high specific surface $SmCoO_3$ and $PrCoO_3$ perovskites. *Cataly. Today* **2009,** *143* (3), 309-314.
20. Zhang, S.; Liu, S.; Zhu, X.; Yang, Y.; Hu, W.; Zhao, H.; Qu, R.; Zheng, C.; Gao, X., Low temperature catalytic oxidation of propane over cobalt-cerium spinel oxides catalysts. *Appl. Surf. Sci.* **2019,** *479*, 1132-1140
21. Zhu, W.; Chen, X.; Li, C.; Liu, Z.; Liang, C., Manipulating morphology and surface engineering of spinel cobalt oxides to attain high catalytic performance for propane oxidation. *J. Catal.* **2021,** *396*, 179-191.
22. Luo, Y.; Zuo, J.; Feng, X.; Qian, Q.; Zheng, Y.; Lin, D.; Huang, B.; Chen, Q., Good interaction between well dispersed Pt and $LaCoO_3$ nanorods achieved rapid Co^{3+}/Co^{2+} redox cycle for total propane oxidation. *Chem. Eng. J.* **2019,** *357*, 395-403.
23. Ren, Z.; Wu, Z.; Song, W.; Xiao, W.; Guo, Y.; Ding, J.; Suib, S. L.; Gao, P.-X., Low temperature propane oxidation over Co_3O_4 based nano-array catalysts: Ni dopant effect, reaction mechanism and structural stability. *Appl. Catal. B: Environ.* **2016,** *180*, 150-160.
24. Chen, L.; Jia, J.; Ran, R.; Song, X., Nickel doping MnO_2 with abundant surface pits as highly efficient catalysts for propane deep oxidation. *Chem. Eng. J.* **2019,** *369*, 1129-1137.
25. Liao, W.-M.; Zhao, P.-P.; Cen, B.-H.; Jia, A.-P.; Lu, J.-Q.; Luo, M.-F., Co-Cr-O mixed oxides for low-temperature total oxidation of propane: Structural effects, kinetics, and spectroscopic investigation. *Chinese J. Catal.* **2020,** *41* (3), 442-453.

26. Zhang, W.; Díez-Ramírez, J.; Anguita, P.; Descorme, C.; Valverde, J. L.; Giroir-Fendler, A., Nanocrystalline Co_3O_4 catalysts for toluene and propane oxidation: Effect of the precipitation agent. *Appl. Catal. B: Environ.* **2020,** *273*, 118894.
27. Zhang, W.; Lassen, K.; Descorme, C.; Valverde, J. L.; Giroir-Fendler, A., Effect of the precipitation pH on the characteristics and performance of Co_3O_4 catalysts in the total oxidation of toluene and propane. *Appl. Catal. B: Environ.* **2021,** *282*, 119566.
28. Liu, S.; Wang, H.; Wang, S.; Dai, Y.; Liu, B.; Liu, Y.; Dang, F.; Smith, K. J.; Nie, X.; Hou, S.; Guo, X., Engineering morphology and Ni substitution of $Ni_xCo_{3-x}O_4$ spinel oxides to promote catalytic combustion of ethane: Elucidating the influence of oxygen defects. *ACS Catal.* **2023,** *13* (7), 4683-4699.
29. Fu, Q.; Wang, S.; Wang, T.; Xing, D.; Yue, X.; Wang, M.; Wang, S., Insights into the promotion mechanism of ceria-zirconia solid solution to ethane combustion over Pt-based catalysts. *J. Catal.* **2022,** *405*, 129-139.
30. Demoulin, O.; Le Clef, B.; Navez, M.; Ruiz, P., Combustion of methane, ethane and propane and of mixtures of methane with ethane or propane on Pd/γ-Al_2O_3 catalysts. *Appl. Catal. A: Gen.* **2008,** *344* (1-2), 1-9.
31. Kucherov, A. V.; Hubbard, C. P.; Kucherova, T. N.; Shelef, M., Stabilization of the ethane oxidation catalytic activity of Cu-ZSM-5. *Applied Catal. B: Environ.* **1996,** *7* (3-4), 285-298.
32. Jian, Y.; Yu, T.; Jiang, Z.; Yu, Y.; Douthwaite, M.; Liu, J.; Albilali, R.; He, C., In-depth understanding of the morphology effect of α-Fe_2O_3 on catalytic ethane destruction. *ACS Appl. Mater. Interfaces* **2019,** *11* (12), 11369-11383
